# Designing for Degradation: Transient Devices Enabled by (Nano)Cellulose

**DOI:** 10.1002/adma.202401560

**Published:** 2024-09-02

**Authors:** Lucas J. Andrew, Erlantz Lizundia, Mark J. MacLachlan

**Affiliations:** ^1^ Department of Chemistry University of British Columbia 2036 Main Mall Vancouver BC V6T 1Z1 Canada; ^2^ Life Cycle Thinking Group Department of Graphic Design and Engineering Projects Faculty of Engineering in Bilbao University of the Basque Country (UPV/EHU) Bilbao 48013 Spain; ^3^ BCMaterials Basque Center for Materials Applications and Nanostructures UPV/EHU Science Park Leioa 48940 Spain; ^4^ Stewart Blusson Quantum Matter Institute University of British Columbia 2355 East Mall Vancouver BC V6T 1Z4 Canada; ^5^ WPI Nano Life Science Institute Kanazawa University Kanazawa 920–1192 Japan; ^6^ UBC BioProducts Institute 2385 East Mall Vancouver BC V6T 1Z4 Canada

**Keywords:** biodegradable, circular economy, nanocellulose, sustainability, transiency

## Abstract

Transient technology involves materials and devices that undergo controlled degradation after a reliable operation period. This groundbreaking strategy offers significant advantages over conventional devices based on non‐renewable materials by limiting environmental exposure to potentially hazardous components after disposal, and by increasing material circularity. As the most abundant naturally occurring polymer on Earth, cellulose is an attractive material for this purpose. Besides, (nano)celluloses are inherently biodegradable and have competitive mechanical, optical, thermal, and ionic conductivity properties that can be exploited to develop sustainable devices and avoid the end‐of‐life issues associated with conventional systems. Despite its potential, few efforts have been made to review current advances in cellulose‐based transient technology. Therefore, this review catalogs the state‐of‐the‐art developments in transient devices enabled by cellulosic materials. To provide a wide perspective, the various degradation mechanisms involved in cellulosic transient devices are introduced. The advanced capabilities of transient cellulosic systems in sensing, photonics, energy storage, electronics, and biomedicine are also highlighted. Current bottlenecks toward successful implementation are discussed, with material circularity and environmental impact metrics at the center. It is believed that this review will serve as a valuable resource for the proliferation of cellulose‐based transient technology and its implementation into fully integrated, circular, and environmentally sustainable devices.

## Graphical abstract

Recent progress in transient devices enabled by (nano)cellulosic materials is reviewed. Transiency mechanisms, advantages of nanocelluloses, and a suite of applications are discussed. A circular thinking approach coupled with life cycle assessment is applied to critically revisit the potential, advantages, and challenges of nanocellulose‐enabled transient devices for future materials innovation.

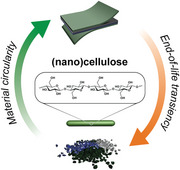

## 1 Introduction

Transient devices have risen to prominence in recent years due to escalating environmental challenges caused by material depletion and non‐degradable waste accumulation.^[^ ^1^, ^2^, ^3^ ^]^ The use of critical raw materials (CRMs) to achieve maximum performance is usually beneficial during device operation, but can cause serious adverse impacts on environmental and human health after disposal.^[^ ^1^, ^2^, ^3^ ^]^ The generation of electronic waste is also reaching unsustainable levels worldwide, with about 50 million metric tons now produced each year; this figure is projected to balloon to a staggering 74 million metric tons by 2030, representing a 100% increase in only 16 years.^[^ ^4^, ^5^ ^]^ This waste is not immediately visible to many of us – it is shipped away to the Global South for “recycling” through hard manual labor or dumping, and the negative environmental impacts due to pollution and the resultant human suffering are immense.^[^ ^4^ ^]^ Representing a paradigm shift in design principles, transient devices do not aim to last forever. Instead, they are fabricated in line with the 10^th^ Green Chemistry principle, *Design for Degradation*, ensuring that they can vanish harmlessly into their environment, or that their degradation products can be upcycled once their working lifetime has passed.^[^ ^6^ ^]^ While fundamentally any device can be considered “degradable” by e.g. thermal or mechanical treatment methods, a “transient” device incorporates degradability in its design from the ground up, optimizing for facile degradation and the generation of reusable or environmentally benign/beneficial degradation products. This approach has great potential to increase the circularity of manufacturing processes, but a balance must be struck between implementing transiency and retaining acceptable performance.^[^ ^1^, ^3^ ^]^

**Figure 1:**
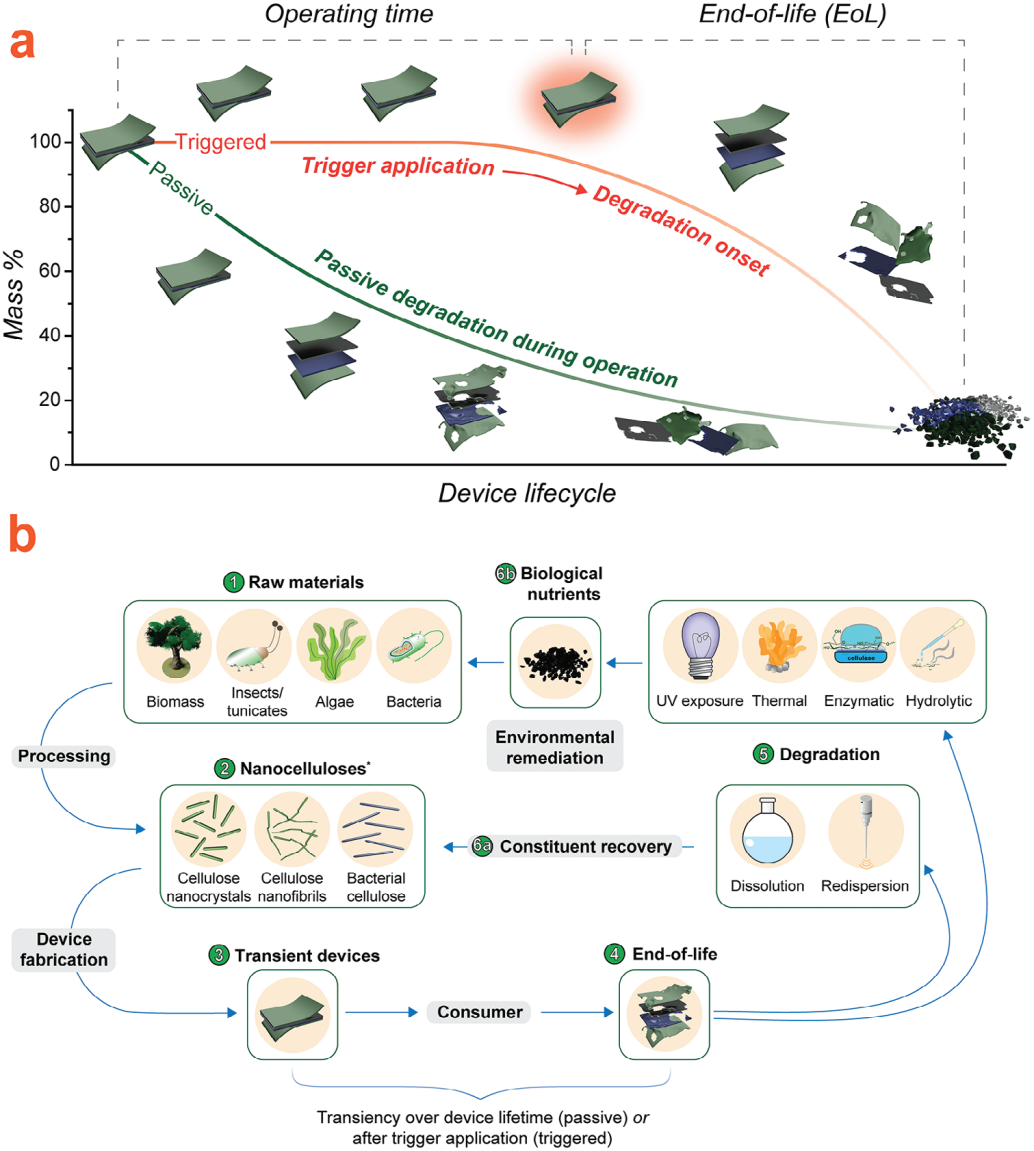
a) Representation of the transient device concept illustrating degradation with either passive or triggered transiency mechanisms. b) Life cycle of a (nano)cellulose‐based transient device, illustrating material circularity by degradation. *Only the most commonly used nanocelluloses are shown here for brevity.

Fundamentally, transiency can be divided into two general categories (**Figure** **1**a). “Passive” transiency manifests in continuous degradation over the operational lifetime without any additional input.^[^ ^2^ ^]^ In contrast, “triggered” transiency requires a stimulus to promote the onset of degradation. Intrinsically, a passive transient device experiences a loss in performance during its operational lifetime due to continuous degradation. This is not necessarily a downside, as “hands‐off” degradation can be extremely useful for certain applications. For example, absorbable sutures made of silk^[^ ^7^ ^]^ or collagen^[^ ^8^ ^]^ are ubiquitous in modern medical practice, reducing the need for invasive follow‐up surgical intervention.^[^ ^9^ ^]^ However, triggered transient devices can be designed with greatly increased operational flexibility.^[^ ^2^ ^]^ As such, in this review, we focus mainly on devices employing triggered transiency.

A wide range of materials have been employed for transient devices. In transient electronics, metals such as Mg, Zn, Fe, W, and Mo, all of which can be incorporated in solution, have been used.^[^ ^2^ ^]^ Semiconductor materials such as Si, Ge, or SiGe alloys can also dissolve under selected conditions.^[^ ^10^ ^]^ However, the most well‐known examples involve the use of (bio)degradable polymers such as poly(lactic‐*co*‐glycolic acid) (PLGA), polylactic acid (PLA), and poly(*ɛ*‐caprolactone) (PCL).^[^ ^2^, ^11^ ^]^ Unfortunately, the majority of these polymers are based on non‐renewable fossil resources. Thus, we believe that naturally available proteins such as silk fibroin, complex organic polymers such as lignin, or natural polysaccharides including chitin, chitosan, and cellulose are especially interesting.^[^ ^3^, ^12^ ^]^ These materials offer the potential for transiency, and their bio‐based nature may enable integration into a more circular cycle of resource usage.^[^ ^3^ ^]^

**Figure 2:**
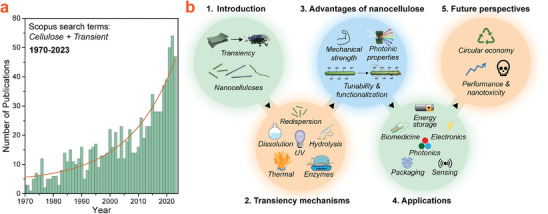
a) Exponential growth trend in the number of publications related to transient devices with cellulosic components. The data were collected using Scopus with “transient AND cellulose” as search terms (June 2024). Only original research articles were included in the count; reviews, perspectives, and conference proceedings were excluded. b) Scope of this review outlining the major themes discussed in each section.

Cellulose is the most abundant natural polymer on Earth. This fact, along with its intrinsic renewability, degradability, biocompatibility, and remarkable properties, makes it an excellent choice for the development of transient devices within a Circular Economy framework, as shown in Figure 1b. Consequently, while still in its infancy, research related to cellulose‐based transient devices is quickly gaining pace (**Figure** **2**a). To enhance its versatility, cellulose can be processed into various nanoscale forms such as cellulose nanocrystals (CNCs), cellulose nanofibrils (CNFs), and bacterial cellulose (BC). CNCs are generally prepared through acid‐catalyzed hydrolysis of lignocellulosic biomass such as wood pulp^[^ ^13^ ^]^ or food waste.^[^ ^14^ ^]^ This treatment selectively removes the amorphous regions of cellulose, leaving behind spindle‐shaped crystalline nanoparticles with varying width (5‐20 nm), length (100‐300 nm), and surface chemistry depending on the cellulose source and processing conditions.^[^ ^13^ ^]^ CNCs have a theoretical Young's modulus similar to Kevlar and can self‐assemble into lyotropic chiral nematic structures in water, resulting in interesting photonic properties.^[^ ^13^ ^]^ On the other hand, CNFs are prepared using mechanical or ultrasonic fibrillation, often coupled with TEMPO‐oxidation resulting in surface carboxylation.^[^ ^15^, ^16^ ^]^ As a result, CNFs have extended, more variable morphologies that contain both crystalline and amorphous regions, allowing for tunability of mechanical properties.^[^ ^17^ ^]^ BC, biosynthesized by bacteria, is highly crystalline with larger aspect ratios than most CNCs.^[^ ^18^ ^]^ Besides, algal cellulose (AC), isolated from natural algae, has similar physical characteristics to BC.^[^ ^18^ ^]^ In this review, we focus mostly on CNC and CNF‐based materials due to their ubiquity in the literature, while some representative examples of BC‐based materials are also included.

Figure 2b outlines the scope of this review, including major topics discussed in each section. While other reviews on transient materials have been published in the past, those works have generally been more limited in scope. For example, some reviews focus on a specific application such as transient electronics,^[^ ^2^ ^]^ food packaging,^[^ ^19^ ^]^ or biomedicine,^[^ ^20^ ^]^ wherein (nano)cellulosic materials are not the main focus. Even in reviews where (nano)cellulosic materials are central, the discussion is generally limited to a specific application such as energy storage,^[^ ^1^, ^21^, ^22^ ^]^ ionic conductors,^[^ ^23^ ^]^ or triboelectric nanogenerators.^[^ ^24^ ^]^ Finally, though reviews touching on a wide range of applications for transient (nano)cellulosic devices exist,^[^ ^3^ ^]^ a detailed description of degradation mechanisms and (nano)cellulose property comparisons is not given, and a quantitative discussion of material circularity and environmental impact is lacking. As such, in this review we expand on previous works to provide a holistic view of (nano)cellulosic materials for transient devices, centering the discussion on material circularity. We first describe degradation mechanisms for nanocellulose‐based transient devices. We then discuss the advantages of using nanocelluloses for transient device design, touching on their intrinsic properties as well as high tunability. Next, we detail various applications of (nano)cellulosic transient devices, showing examples in sensing, photonics, energy storage, electronics, food packaging, biomedicine, and wastewater treatment. Finally, future perspectives and challenges in terms of environmental sustainability, circularity, and industrialization are discussed. The importance of nanotoxicity assessment, especially for surface‐modified nanocelluloses, is also discussed since the diverse property changes afforded by modification can drastically affect human and environmental impacts. As a result, this review provides a holistic view of the rapidly burgeoning field of (nano)cellulose‐enabled transient devices, offering a comprehensive starting point for researchers looking to break into the field.

## 2 Transiency Mechanisms for (Nano)Cellulosic Materials

Transiency can be realized through a wide range of mechanisms. Previous reviews^[^ ^1^, ^2^, ^3^ ^]^ on transient materials have reported transiency mechanisms ranging from thermal and UV‐induced degradation to simple dissolution.^[^ ^2^, ^25^ ^]^ The choice of transiency mechanism is important: according to Fu et al., only dissolution‐based mechanisms provide a complete degradation pathway, while other degradation methods allow for varying degrees of partial transiency which may or may not be appropriate depending on the application.^[^ ^2^ ^]^

Although passive mechanisms can be appropriate for some scenarios, triggered degradation mechanisms allow for better tuning of device applications. Triggered mechanisms enable device operation without performance loss during its lifespan, with degradation only beginning once the trigger is applied. This trigger may be supplied by operator input or intrinsically built into the device. Importantly, the required trigger should be simple to apply to limit operator burden. Furthermore, a single transiency mechanism may not be sufficient to achieve the desired degradation; instead, multiple degradation pathways may be applied in tandem to achieve the optimal effect for a given material.

**Figure 3:**
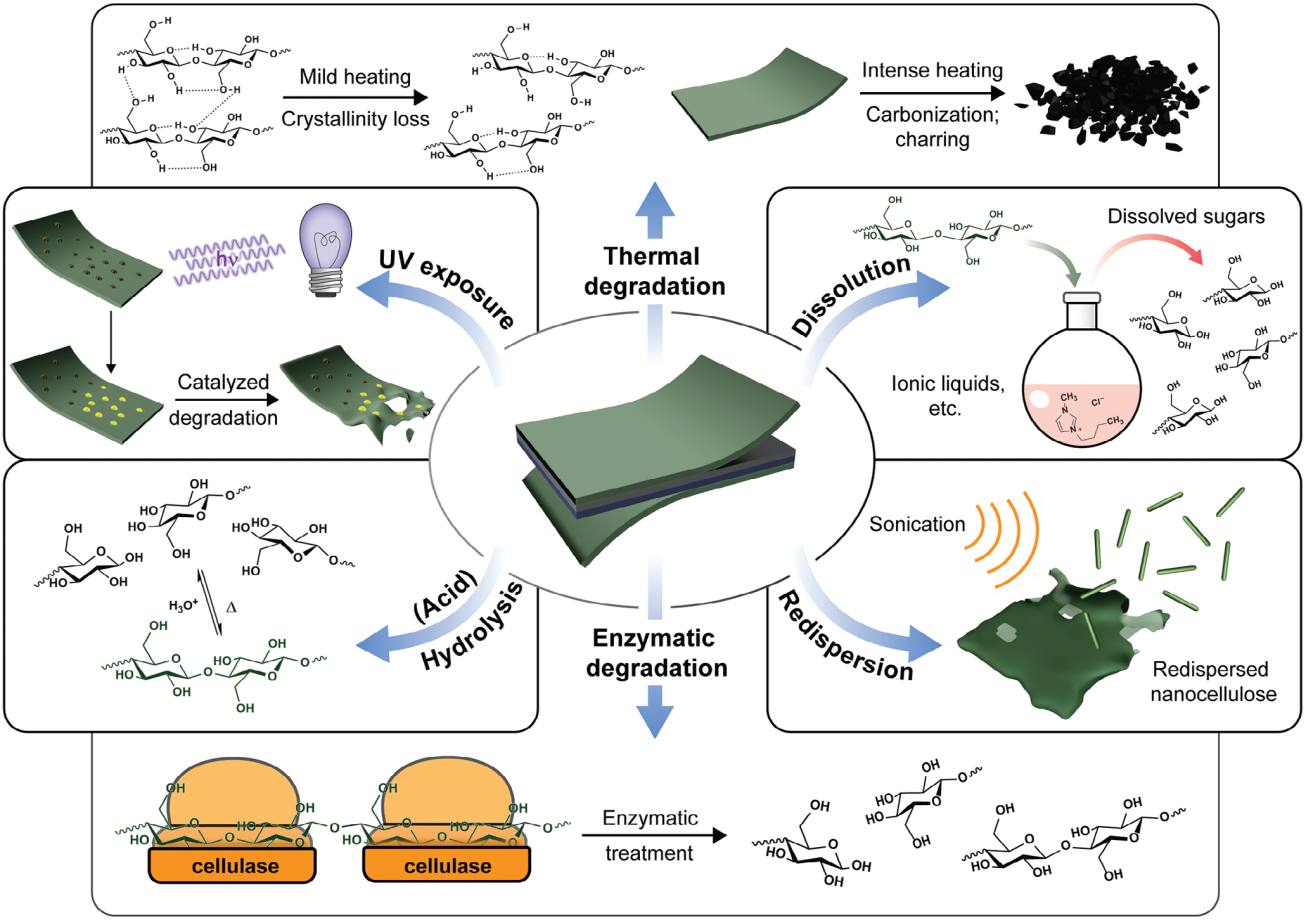
Overview of the most common transiency mechanisms exploited in (nano)cellulose‐based devices.

For nanocellulose‐based materials, transiency mechanisms associated with polymeric materials such as hydrolysis, thermal, and enzymatic^[^ ^25^ ^]^ degradation are accessible (**Figure** **3**). Each of these mechanisms can influence others, necessitating careful consideration of the interplay between different degradation pathways during materials design. This section explores these various transiency mechanisms in detail, including recent examples, advantages, and applications of each approach, as well as areas needing further development.

### 2.1 Dissolution of (Nano)Cellulose

Without additional pre‐processing, the dissolution of cellulose presents certain issues. The solubility of cellulose in water is very poor,^[^ ^26^ ^]^ a fact ascribed to strong hydrogen bonding between cellulose chains. One must also consider kinetics, which correctly predicts poor solubility due to the low conformational flexibility of cellulose chains, resulting in a weaker entropic driving force for dissolution.^[^ ^26^ ^]^ Other reports also suggest that the amphiphilic nature of cellulose chains is responsible for its crystallinity and packing (i.e. through inter‐chain hydrophobic interactions),^[^ ^26^, ^27^, ^28^ ^]^ as well as the observed tendency for cellulose to only dissolve in amphiphilic solvents.^[^ ^26^, ^29^ ^]^ As numerous detailed reviews^[^ ^26^, ^29^, ^30^, ^31^ ^]^ have already been published on the mechanisms of cellulose dissolution, here we provide only a basic overview to motivate discussions of targeted mechanisms and applications later on.

Since the advent of nitrocellulose‐based materials in the 1800s,^[^ ^32^ ^]^ a variety of techniques have been employed to enable solution processing of cellulose. For example, the viscose process, which involves the swelling of cellulose with aqueous NaOH followed by a reaction with CS_2_, is commonly used to produce the textile rayon.^[^ ^26^ ^]^ This same material can be produced by the now‐defunct cuprammonium process, which employs a solution of Cu(OH)_2_ in ammonia to functionalize vicinal hydroxyl groups on the cellulose chain, resulting in the disruption of inter‐chain hydrogen bonds.^[^ ^33^ ^]^ These methods are reliable and scalable for industrial use, but the processing conditions required preclude their use in transient devices except perhaps in a large‐scale recycling context.^[^ ^26^, ^30^ ^]^ At the lab scale, organic solvent/salt mixtures such as N,N‐dimethylacetamide (DMAc)/LiCl, or tetrabutylammonium fluoride/dimethyl sulfoxide offer milder processing conditions in exchange for scalability.^[^ ^26^, ^30^, ^31^ ^]^ In these systems, dissolution involves the accumulation of the anion along the cellulose chain, creating a negatively charged polymer that is stabilized by a macrocation (e.g. Li‐DMAc). Thus, the combination of osmotic pressure forcing solvent between the cellulose chains and charge repulsion enables dissolution.^[^ ^26^, ^30^, ^34^, ^35^ ^]^ As solvents such as DMAc have also been used as electrolytes or electrolyte‐stabilizing additives in Li‐ion batteries,^[^ ^36^, ^37^, ^38^ ^]^ intriguing possibilities may exist for the use combination of transient (nano)cellulosic components in such devices. Aqueous salt solutions such as ethylenediamine/KSCN and solutions of NaOH in very narrow concentration ranges have also been shown to dissolve cellulose, but their use is less widespread.^[^ ^30^, ^32^ ^]^

Ionic liquids (ILs) are also very useful in cellulose processing. They are highly thermally stable, have high solvation ability, and present a wide electrochemical stability window, making them ideal for a wide range of applications.^[^ ^39^ ^]^ The dissolution of cellulose in an IL was first shown in 1934 with N‐alkylpyridinium chloride,^[^ ^40^ ^]^ but was not widely studied until after the discovery in 2002 of ILs with high dissolution ability, such as [Bmim][Cl], [Emim][OAc], and [Amim][Cl].^[^ ^29^, ^41^ ^]^ The mechanism of cellulose dissolution by ILs is not entirely understood, but a number of trends have been observed: firstly, ILs with weakly H‐bonding anions have been shown to be ineffective solvents for cellulose,^[^ ^39^ ^]^ suggesting that disruption of cellulose H‐bonds is important for dissolution. Secondly, increasing symmetry or side chain length of cations negatively affects cellulose solubility due to increased viscosity. Finally, the solubility of cellulose in ILs with different cations generally decreases according to the following trend: imidazolium > pyridinium > ammonium, a phenomenon potentially related to cation polarizability.^[^ ^39^ ^]^ ILs are generally considered non‐derivatizing solvents (i.e. they do not modify or degrade the cellulose upon dissolution).^[^ ^39^ ^]^ While recent work has shown that dissolution in ILs can in fact induce a decrease in cellulose molecular weight (MW) after regeneration,^[^ ^42^ ^]^ the potential for material reuse with minimal reduction in material properties^[^ ^43^ ^]^ after degradation of a transient device is attractive for in a circularity context. However, although certain recently developed ILs have shown low cost and limited toxicity, conventional ILs are expensive and often toxic, limiting the practical application of large‐scale (nano)cellulose processing.^[^ ^44^, ^45^, ^46^ ^]^

Deep eutectic solvents (DESs) are also emerging as candidates for environmentally friendly processing of (nano)celluloses. First reported in 2001 by Abbott et al.^[^ ^47^ ^]^ like ILs, DESs consist of a mixture of acids and bases (either Lewis or Brønsted). However, DESs can contain multiple different species that form a homogenous eutectic mixture; i.e. a mixture with a melting point lower than that of the individual constituent components.^[^ ^48^ ^]^ Usually, these consist of a quaternary ammonium salt complexed with either a metal salt or hydrogen bond donating moiety, e.g. mixtures of choline chloride and urea as in Abbott et al.’s first report.^[^ ^47^ ^]^ Other examples of DESs to dissolve cellulose are mixtures of amino acids such as histidine and proline, and naturally‐derived acids such as malic acids and lactic acids.^[^ ^49^ ^]^ Like in ILs, dissolution of (nano)celluloses in DESs likely proceeds through disruption of H‐bonds between cellulose chains by H‐bonding groups on the DES component molecules.^[^ ^50^ ^]^ This ability to disrupt interchain H‐bonding depends on the available number of H‐bonding donor and acceptor sites on the DES constituents, and the proportion of the hydrogen bond donor material in the DES mixture.^[^ ^50^ ^]^ In spite of their low technology readiness levels, DESs are a promising alternative to ILs for the processing and dissolution of nanocellulose‐based transient devices owing to their sustainability, non‐toxicity, low cost, and potential scalability.^[^ ^51^, ^52^, ^53^ ^]^

**Figure 4:**
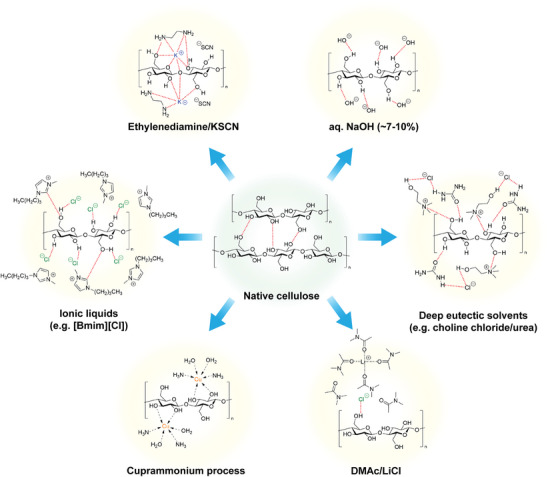
Cellulose dissolution mechanisms discussed in this section. Hydrogen bonds are shown as dotted red lines to illustrate the disruption of cellulose‐cellulose inter‐fiber hydrogen bonds as a prerequisite for dissolution.

Although there are some inherent challenges in applying dissolution‐based degradation in transient (nano)cellulosic devices, it remains a popular method due to its simplicity. Dissolution is the most straightforward strategy to implement in situations where it is possible to switch solvents from a non‐dissolving to a dissolving one after operation, or where passive degradation due to slow dissolution is acceptable for the performance of a material. Dissolution is also often aided by other complementary mechanisms, as will be discussed later. Environmental degradation, for instance, may involve dissolution, but only after partial breakdown by enzymes. Nevertheless, it is essential to comprehend the solubility and dissolution of cellulosic materials when designing transient devices, regardless of the primary transiency mechanism that is employed. A summary of the dissolution mechanisms discussed in this section is provided in **Figure** **4**. In any case, due to the relative ease of processing and facile design, dissolution‐based degradation can be a very useful strategy in transient device development, whether it is implemented on its own or with the assistance of pre‐treatments.

### 2.2 Redispersion of (Nano)Celluloses

Redispersion of nanocelluloses is an important consideration in logistics, as the transport of nanocelluloses in dried form is much more economically viable than transport in suspension.^[^ ^54^ ^]^ As a transiency mechanism, redispersion differs from dissolution in that full solubility is not required; instead, nanocelluloses need only form a reasonably stable suspension to facilitate device degradation. However, dehydrating cellulose can cause the formation of effectively irreversible inter‐fiber H‐bonds in a process known as hornification.^[^ ^54^ ^]^ These bonds typically form when the solid content reaches > 20 wt% and make redispersion from a dried state problematic.^[^ ^54^ ^]^ This effect is enhanced in nanocelluloses, where high surface area and increased accessible surface hydroxyl groups enhance increased aggregation.^[^ ^54^, ^55^ ^]^ Ultrasonication is the most commonly applied method for nanocellulose redispersion.^[^ ^56^, ^57^, ^58^, ^59^, ^60^ ^]^ Numerous studies have outlined best practices for dispersion by ultrasonication, including the ideal amount of energy delivery, the timing of ultrasonication pulses,^[^ ^61^ ^]^ and the effect of ultrasonicator probe position within the sample container.^[^ ^62^ ^]^ Intuitively, nanocelluloses with higher initial colloidal stability are more amenable to redispersion. For example, sulfuric acid is often used during CNC production, installing sulfate half‐ester groups on the surface. This imparts a negative surface charge (zeta potential ca. −40 mV), increasing colloidal stability.^[^ ^13^ ^]^ For transient nanocellulosic devices, it is essential to achieve a high degree of redispersibility. Therefore, research efforts often focus on the application of different drying methods or pre‐treatments to improve redispersibility of the nanocellulose.

**Figure 5:**
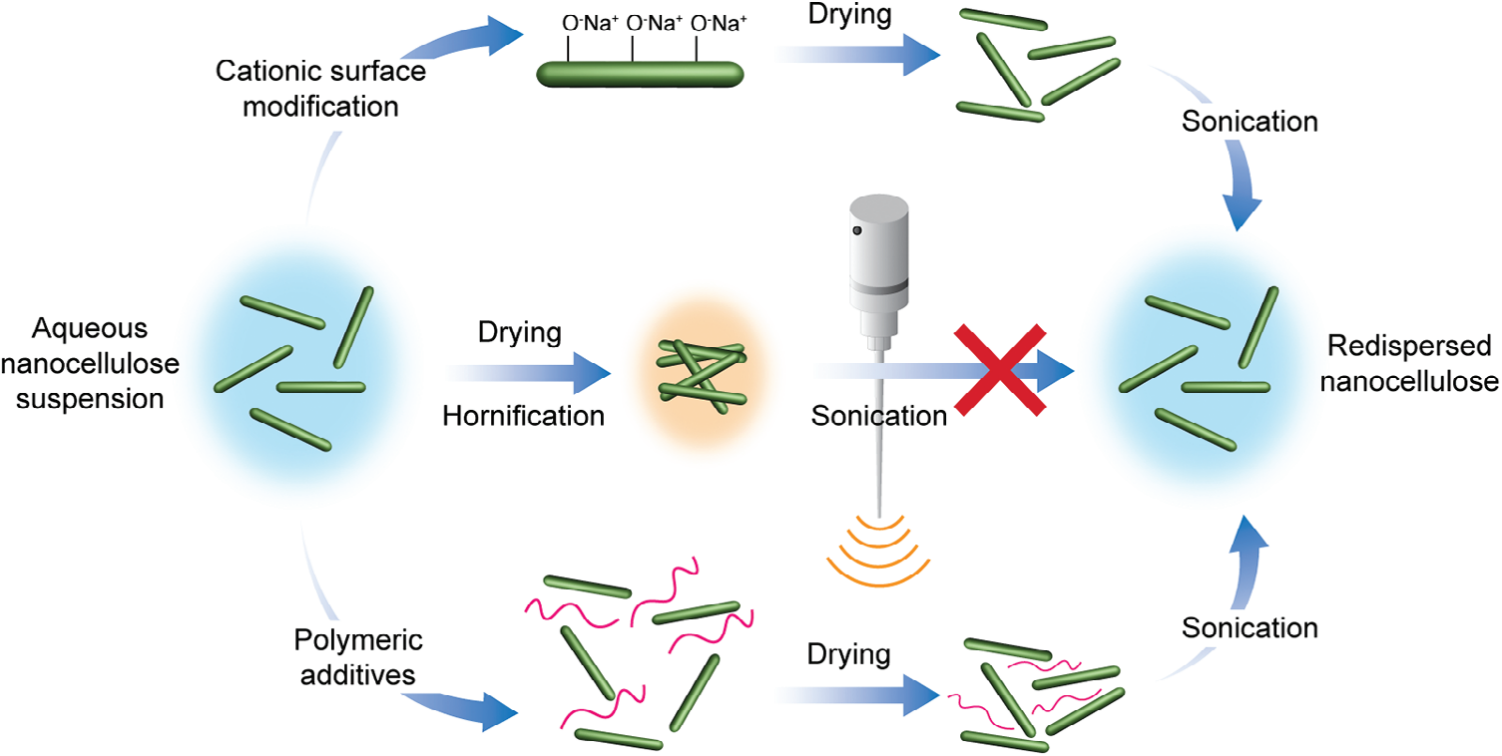
Challenges arising during redispersion of nanocelluloses due to hornification. Representative general strategies to overcome these challenges by surface modification are given.

There are several commonly applied methods to dry nanocelluloses. Oven‐drying and spray‐drying are the cheapest and most scalable but result in the formation of persistent aggregates. Freeze‐drying can also be applied; this method produces fewer aggregates but is more costly. Finally, supercritical‐CO_2_ drying is often used in lab‐scale studies to avoid aggregate formation. This method preserves the individual nanocellulose particles the most effectively but is expensive and time‐consuming.^[^ ^63^, ^64^, ^65^ ^]^ Since drying methods that can produce redispersible nanocelluloses are often less scalable, this hindrance to industrial development leads many researchers to turn to modification processes to create sufficiently redispersible nanocelluloses (**Figure** **5**).^[^ ^54^ ^]^

Improvements to nanocellulose redispersibility can be achieved by chemical surface modification, the inclusion of additives, increasing steric hindrance between components, and cationic modification with salts.^[^ ^54^ ^]^ Cationic modification is very common and involves the simple replacement of the surface hydroxyls with O^−^Na^+^ groups, increasing redispersibility through charge‐charge repulsion.^[^ ^57^ ^]^ Steric hindrance can be increased by polymer grafting, which in addition to increasing redispersibility may also allow the dispersion of nanocelluloses in organic solvents.^[^ ^66^, ^67^, ^68^ ^]^ The surface groups of nanocellulosic materials deserve special attention in regulating dispersion stability. While physically crosslinked materials are easily redispersed, chemical crosslinking or the introduction of stronger non‐covalent interactions can inhibit redispersibility. The inclusion of additives such as carboxymethyl cellulose (CMC) is also common and can increase redispersibility through steric effects,^[^ ^69^ ^]^ and more advanced approaches such as the inclusion of amine‐based CO_2_‐switchable additives have been explored to design materials with controllable material properties and dispersibility.^[^ ^70^, ^71^ ^]^ Combining these modifications with the more scalable drying methods mentioned earlier may facilitate the production of highly redispersible nanocelluloses at a lower cost. Additionally, using more redispersible nanocelluloses can decrease the energy input (e.g. for ultrasonication) required to realize transiency after end‐of‐life (EoL). While redispersion‐based transiency requires a careful initial design to enable redispersibility after drying, it can be a useful transiency mechanism due to its potential to enable circularity.

### 2.3 UV‐Induced Degradation of (Nano)Cellulose

Pristine cellulose does not degrade substantially under UV exposure. To fabricate UV‐responsive cellulosic materials, it is usually necessary to incorporate photocatalytic components. Semiconductor nanoparticles, especially TiO_2_,^[^ ^72^, ^73^, ^74^, ^75^, ^76^ ^]^ have been commonly used, as well as other materials such as cellulose‐based carbon dots,^[^ ^77^ ^]^ Ag@AgCl,^[^ ^78^ ^]^ or Cr_2_O_3_.^[^ ^79^ ^]^ However, often the goal of fabricating these cellulose‐based composites is not the degradation of the cellulose itself, but instead the degradation of dye pollutants under UV–vis light exposure.^[^ ^75^, ^76^, ^78^, ^79^ ^]^ In these systems, cellulose acts as a scaffold to enhance available surface area and facilitate dye‐photocatalyst interactions through enhanced dye adsorption. These studies warn that excessive UV exposure or excessive temperatures could cause degradation of the cellulose scaffold itself.^[^ ^76^ ^]^ Indeed, this would be undesirable for the applications mentioned above, but can potentially be harnessed for use in transient devices.

As an example, Yadav et al. showed that incorporating nano‐graphene oxide‐type carbon dots synthesized from cellulosic precursors can catalyze the UV‐induced deacetylation of cellulose acetate (CA), allowing for degradation of the composite in normal environmental conditions. They demonstrated that after 30 days of UV aging, the CA‐carbon dot films were degraded by 53% in air and 43% in simulated sea water, compared to 12% and 4% in films without carbon dots.^[^ ^77^ ^]^ While they did not attempt to use the cellulose degradation products for further reactions, other researchers have used similar methods to produce 5‐hydroxymethylfurfural or H_2_ gas. Speltini et al. demonstrated that suspending cellulose in water along with Pt‐doped TiO_2_ nanoparticles enabled the use of cellulose as a sacrificial agent for the production of H_2_ gas from water.^[^ ^74^ ^]^ Zhang et al. built upon this idea, fabricating cellulose‐coated Pt‐doped TiO_2_ nanoparticles; again, cellulose functioned as a sacrificial agent, allowing the researchers to convert up to ≈90% of the cellulose to both H_2_ and CO_2_ gas in an approximately 2:1 ratio.^[^ ^73^ ^]^

These examples demonstrate that UV‐induced degradation mechanisms have potential for developing nanocellulose‐based transient devices when combined with appropriate photocatalytic materials. This is due to their ability to degrade cellulose and render it into potentially useful materials after the lifespan of the device has been completed. This transiency mechanism could be intrinsically built into a device by incorporating dual‐function photocatalytic components that do not cause cellulose degradation during the service life but are able to degrade the device when exposed to a light source at their EoL.

### 2.4 Hydrolytic Methods for (Nano)Cellulose Production and Degradation

**Figure 6:**
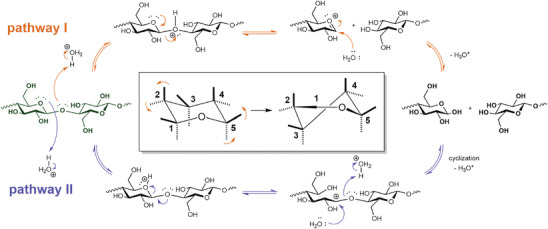
Possible mechanistic pathways for the acid‐catalyzed hydrolysis of cellulose. Center inset: conformational changes of the pyranose ring during the second step of pathway I. Reproduced from with permission.^[^ ^82^ ^]^ Copyright 2009, Chemistry Europe.

Chemical hydrolytic methods have often been used to produce nanocelluloses since the 1940s,^[^ ^80^, ^81^ ^]^ especially for CNCs.^[^ ^13^ ^]^ Usually, mineral acid (e.g. HCl, H_2_SO_4_)‐catalyzed hydrolysis of cellulose in water is used. The mechanism of this process is not completely understood, but clues can be gleaned from mechanistic studies on model disaccharides.^[^ ^82^ ^]^ **Figure** **6** shows two possible reaction pathways. Pathway I is expected to be favored given information from disaccharide analogs, but should be less energetically favorable for cellulose as the crystalline structure may hinder the rotation required to form the carbocation intermediate (Figure 6, Inset).^[^ ^82^ ^]^ Thus, pathway II is also a possibility for cellulose, but experimental evidence for this mechanism remains lacking. Mechanism aside, acid‐catalyzed hydrolysis of cellulose is not limited to mineral acids in aqueous conditions. Organic acids such as citric and malonic acid have also been used, and also impart surface functionality during hydrolysis.^[^ ^13^ ^]^ Hydrolysis in subcritical^[^ ^83^ ^]^ and supercritical^[^ ^84^, ^85^ ^]^ water conditions have also been studied, in addition to acid‐catalyzed hydrolysis in ILs.^[^ ^86^, ^87^ ^]^

Hydrolysis of cellulosic materials in milder, environmentally relevant conditions has also been investigated. In 2022, Yadav and Hakkarainen studied the dissolution of CA films in lake water, artificial, and natural seawater, saturated NaCl solution, tap water, and dilute NaOH solution.^[^ ^88^ ^]^ At ambient temperatures, the samples showed only ≈5% weight loss after one month in all conditions except for in dilute NaOH, where degradation increased to ≈10%, a result attributed to the accelerated rate of deacetylation under alkaline conditions. Degradation was accelerated at 60 °C, with ≈5–14% and ≈30% weight loss for non‐NaOH and NaOH conditions, respectively.^[^ ^88^ ^]^ Furthermore, a decrease in the number average molecular weight (M_n_) and molar‐mass dispersity of CA was also observed in the first few months of aging for all samples. Studies like these are valuable for highlighting the limitations of environmental dissolution conditions and the need for additional intervention if accelerated transiency pathways are required.

Overall, hydrolytic mechanisms are very useful in transient (nano)cellulosic devices. For instance, energy storage devices using acidic aqueous electrolytes could be used for passive transiency, enabling a gradual breakdown of the device. Hydrolysis is also simple to implement in industrial recycling applications where transient devices can be degraded in bulk, allowing for large‐scale materials recovery. Importantly, acidic hydrolysis is a simple, scalable, and potentially cost‐effective method for processing transient devices. However, it is not as versatile as other mechanisms that may permit complete degradation in the natural environment.

### 2.5 Enzyme‐Catalyzed Degradation of (Nano)Celluloses

Enzymatic degradation of cellulosic biomass has been studied to produce nanocelluloses since the 1950s,^[^ ^89^, ^90^ ^]^ and this approach can be exploited to develop transient devices. The cellulase‐catalyzed breakdown of cellulose proceeds in a stepwise fashion: first, adsorption of the enzyme at the surface to form an enzyme‐substrate complex, followed by hydrolysis of the cellulose into cellodextrins and cellobiose, and finally further degradation of these intermediate products into glucose.^[^ ^90^ ^]^ The rate of enzymatic degradation is largely governed by enzyme adsorption on the cellulose surface. As a result, enzyme type, concentration, and available cellulose surface area, and crystallinity are highly important.^[^ ^90^, ^91^ ^]^ Cellulases can be roughly divided into three main classes: endoglucanases, cellobiohydrolases, and *β*‐glucosidases,^[^ ^92^ ^]^ the latter two being of particular importance for nanocellulose degradation. Cellobiohydrolases are able to degrade crystalline celluloses,^[^ ^90^ ^]^ starting from the hydrophobic face of the cellulose crystal^[^ ^93^ ^]^ As a result, treatments that enhance the accessibility of this hydrophobic face, such as ammonia treatment to convert the cellulose to cellulose III_I_, are beneficial.^[^ ^94^ ^]^ On the other hand, *β*‐glucosidases mainly degrade cellobioses,^[^ ^90^, ^95^ ^]^ suggesting that a combination of these two enzymes may be useful for achieving transiency.

Literature showing the enzymatic degradation of nanocelluloses is abundant. For example, Hossain et al. fabricated superabsorbent polymer gels from TEMPO‐oxidized CNCs, with and without hexamethylenediamine crosslinks. Both the crosslinked and non‐crosslinked gels were degraded by cellulases under simulated compost conditions (35 °C, 80% humidity), with the non‐crosslinked and crosslinked gels degrading by 41 and 56 wt%, respectively, after 2 days.^[^ ^96^ ^]^ They proposed that the increased degradability of the crosslinked gels was due to the nitrogen content of the crosslinker.^[^ ^96^ ^]^ In contrast, Pahwa and Ahuja employed crosslinked CNC‐gellan scaffolds for the intravaginal delivery of the antifungal agent fluconazole, and found that degradation by lysozymes reached 90% for non‐crosslinked samples after 28 days, but reached only 40% for crosslinked samples.^[^ ^97^ ^]^ However, sodium trimetaphosphate was used as a crosslinker, suggesting that crosslinking with non‐nitrogen‐containing groups may slow enzymatic degradation.^[^ ^97^ ^]^ Indeed, surface modification of nanocelluloses affects the rate and extent of enzymatic degradation, as access to the nanocellulose surface can become problematic with high grafting density or when bulky molecules are attached.^[^ ^98^ ^]^ Control of enzymatic degradation rate can also be achieved through morphological tuning, as shown by Das et al., who 3D‐printed unmodified BC‐CNF hydrogels with different geometries and infill densities to modulate the time required to reach 100% degradation by cellulase.^[^ ^99^ ^]^ Clearly, with careful consideration of nanocellulose morphology, modification, and enzymatic conditions, an optimal degradation profile for each given application can be achieved. Enzymes can even be incorporated within the material so that degradation events are simultaneously catalyzed both at the surface and in the bulk, greatly increasing degradation rate.^[^ ^100^ ^]^

**Figure 7:**
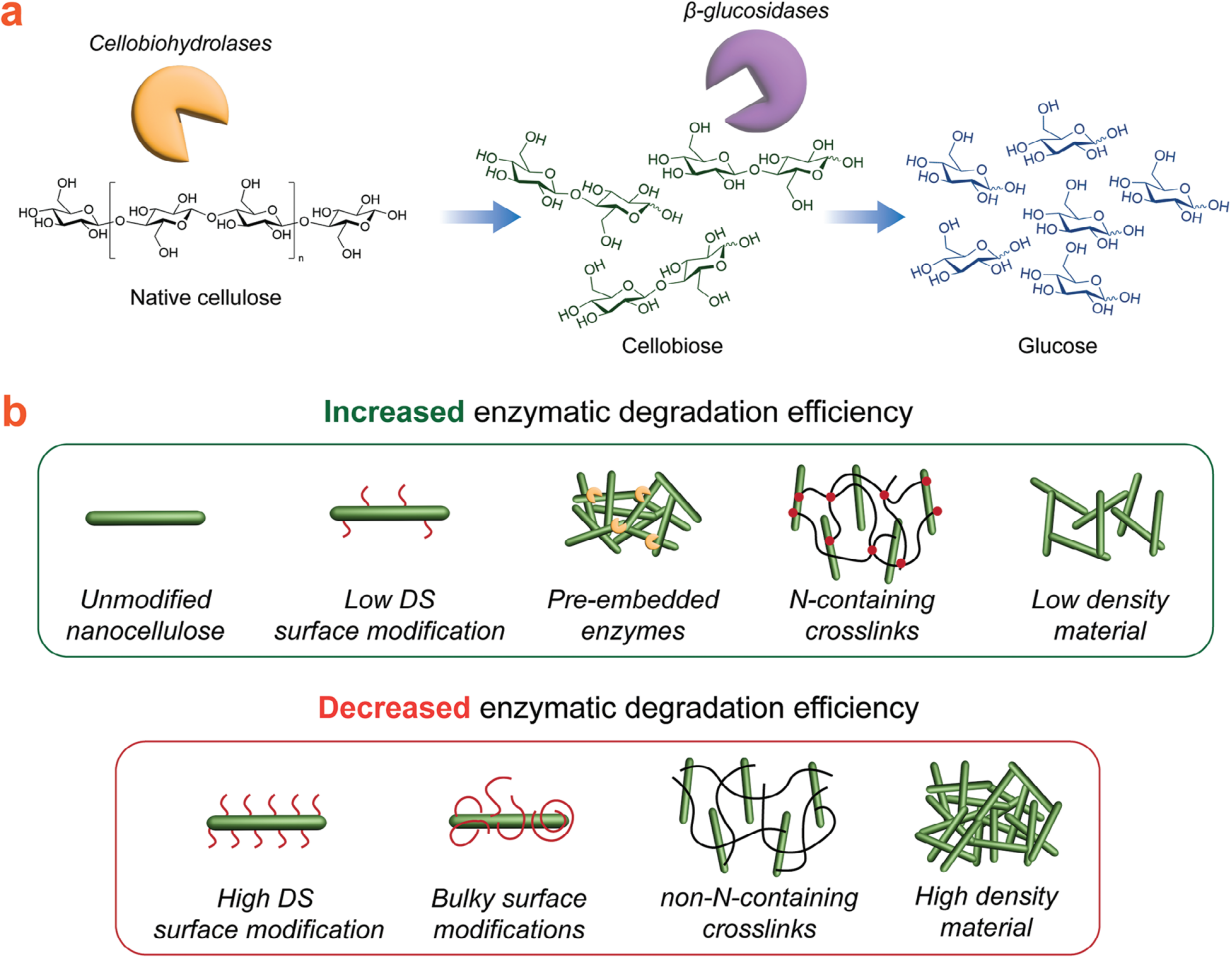
a) Simplified representation of the stepwise degradation of cellulose by a combination of cellobiohydrolase and β‐glucosidase enzymes. b) Graphical representation of tuning the efficiency of enzymatic degradation in nanocellulose‐based materials.

Enzymatic degradation can be useful in the future for transient device design as single‐use devices are usually discarded haphazardly into the environment after use. To this end, Erdal and Hakkarainen recently reviewed the degradation of various cellulosic materials in different natural environments.^[^ ^101^ ^]^ Cellulose derivatives such as CA, cellulose acetate propionate, CMC, cellulose xanthate, and cellulose octanoate were studied, revealing a concomitant effect of both degree of substitution (DS) and substituent chemistry. The degradation of substituted celluloses often requires a combination of enzymes, as initial removal of surface groups is necessary to allow cellulases access to the cellulose backbone itself.^[^ ^101^ ^]^ This effect was demonstrated by Leppänen et al., who found a clear negative correlation between both DS and substituent length and enzymatic hydrolysis rate in a laboratory environment.^[^ ^102^ ^]^ This correlation has also been shown in wastewater, as well as fresh‐ or seawater. However, the presence of other components in wastewater (e.g. Zn^2+^, Fe^3+^) may inhibit enzymatic activity,^[^ ^101^ ^]^ and degradation rates in fresh‐ and seawater can vary significantly depending on the ionic and microbial contents of the medium. High variability in soils is also observed depending on moisture and microbial content, among other factors. This high variability of degradation behavior due to environmental differences, the type of the enzymes, and type/modification of nanocellulose can make enzymatic degradation difficult to grasp. Therefore, to assist the reader, a summary of the materials and processes discussed in this section is included in **Table** **1**. A simplified representation of enzymatic degradation along with the effects of some tunable factors can also be found in **Figure** **7**.

**Table 1:** Comparison of enzymatic degradation conditions, degradation rate, specific highlights, and considerations for all references mentioned in this section.

Material	Enzymatic degradation conditions	Degradation rate	Highlighted results	Refs.
TEMPO‐CNCs crosslinked with hexamethylenediamine	Cellulase (*Trichoderma reesei*) (50/100 µL /7 mL H_2_O); Soil (60% moisture); 35 °C, 80% RH, 2–6 days	41 wt% in 2 days (non‐crosslinked); 56 wt% in 2 days (crosslinked)	N‐containing crosslinker promotes enzymatic degradation under simulated compost conditions	[96]
CNC‐gellan scaffolds containing fluconazole crosslinked with Na_3_P_3_O_9_	Lysozyme (2 × 10^6^ IU in simulated vaginal fluid, replaced 1x per 2 days); 37 °C, 28 days	90 wt% in 28 days (non‐crosslinked); 40 wt% in 28 days (crosslinked)	Non N‐containing crosslinker can delay degradation in simulated vaginal fluid (potential to tune treatment duration)	[97]
CNFs functionalized with hexyl/dodecyl/phenyl ethers/esters @ varying DS	Anaerobic wastewater sludge 28 °C, 1–424 days	100 wt%^a)^ in ≈80 days (hexyl ester, DS: 1.19); 70 wt%^a)^ in ≈80 days (hexyl ester, DS: 2.43); etc.	DS at nanocellulose surface negatively impacts enzymatic degradation	[98]
Films from various cellulose derivatives, e.g. CA, methyl cellulose	Cellulase, mannase, xylanase, β‐glucosidase mixture (31.6 FPU mL^−1^); 0.1 NaOAc buffer; 40 °C, 2 days	≈88 wt% (nanocellulose, DS: 0); ≈55 wt% (butylated hemicellulose, DS: ≈0.4); ≈5 wt% (methyl cellulose, DS: ≈1.7)	DS has a much higher negative impact on enzymatic degradation than bulkiness of substituent groups	[102]
3D‐printed TEMPO‐oxidized bacterial CNF hydrogels at varying infill densities	Cellulase (*Trichoderma reesei*) (150 µg mg^−1^ hydrogel); Cell culture medium; 37 °C, 5% CO_2_, 26 h	All samples degraded 100% in: 5 h (12.5%, 18% infill density); 10 h (25% infill density); 15 h (50% infill density); 26 h (100% infill density)	Nanocellulose degradability can be tuned through morphological adjustments	[99]
CA films with embedded lipases	Lipase (*Candida rugosa*) (3/5% in films, 0.1 mg mL^−1^ in solution); Cellulase (*Aspergillus niger*) (0.1mg mL^−1^ in solution); PBS, 37 °C, 40 days *or* Simulated compost, 58 °C, 60–180 days	PBS (40 days): 88 wt% (5% lipase); 73 wt% (3% lipase); 48 wt% (0% liapse); Simulated compost (60 days): 89 wt% (5% lipase); 60 wt% (0% lipase)	Embedding enzymes within (nano)cellulosic materials can accelerate degradation	[100]

^a)^ Relative to unfunctionalized CNF’.

### 2.6 Thermal Degradation of (Nano)Cellulose

**Figure 8:**
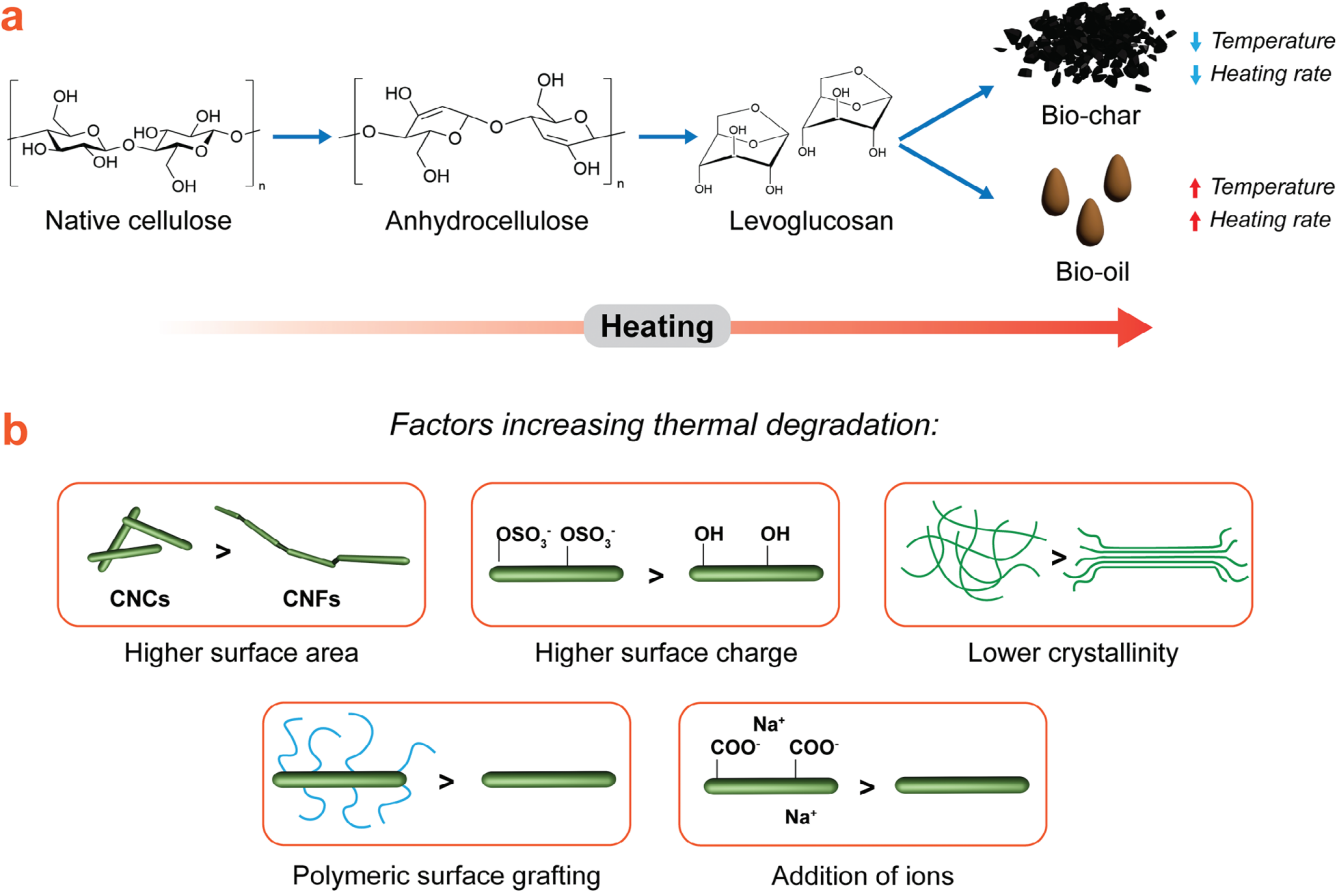
a) Simplified degradation pathway for thermal degradation (pyrolysis) of (nano)celluloses. b) Representative strategies to accelerate the thermal degradation of (nano)celluloses.

The thermal degradation of nanocellulose has been thoroughly reported recently.^[^ ^18^, ^103^, ^104^, ^105^, ^106^, ^107^ ^]^ Bulk cellulose reaches peak degradation ≈350 °C, corresponding to the high reactivity of cellulose *β*‐1‐4 glycosidic linkages at this temperature.^[^ ^18^ ^]^ Several mechanisms for the thermal degradation of cellulose are often considered, the most common being dehydration of the cellulose followed by charring of the resultant anhydrocellulose units,^[^ ^108^ ^]^ or depolymerization into levoglucosan, which is further degraded into tar/bio‐oil (**Figure** **8**a).^[^ ^109^ ^]^ Using slower heating rates and lower temperatures instead promotes the formation of bio‐char.^[^ ^108^ ^]^ Nanocelluloses generally have lower thermal stability than bulk cellulose. For CNCs, an increase in surface area and higher number of accessible reducing ends increases susceptibility to thermal degradation.^[^ ^18^, ^105^, ^106^ ^]^ Additionally, increased surface charge (usually due to sulfate half‐ester groups) is negatively correlated with thermal stability, as surface groups catalyze hydrolysis of the cellulose chains (Figure 8b).^[^ ^105^ ^]^ Other factors such as cellulose crystallinity are also considered.^[^ ^18^ ^]^ A generalized hierarchy of thermal stability can be proposed as follows based on previous works: CNCs < CNFs ≈ tunicate cellulose ≈ BC < AC.^[^ ^18^ ^]^ Understandably, most research focuses on increasing the thermal stability of nanocelluloses; however, for transient devices, the opposite effect may instead be desirable.

Additives can also influence the thermal stability of nanocelluloses. For example, metal ions or nanoparticles are often included in nanocellulose‐based composites to varying effect. While Na^+^‐stabilized CNCs show higher thermal stability,^[^ ^105^ ^]^ the addition of Na^+^ ions to TEMPO‐oxidized (carboxylated) CNFs was shown by Lichtenstein and Lavoine to decrease the thermal degradation onset temperature by 20 °C.^[^ ^109^ ^]^ In a similar vein, Goikuria et al. demonstrated an increase in CNC thermal stability by 75 °C upon incorporation of 10 wt% Fe_2_O_3_ or ZnO in composite films.^[^ ^110^ ^]^ Andrew et al. observed a marked decrease in thermal stability for CNC aerogels containing ≈14 wt% Fe_3_O_4_ nanoparticles, but this effect was reversed for aerogels containing a similar amount of Co_3_O_4_ nanoparticles.^[^ ^65^ ^]^ Considering nanocellulose‐polymer composites, Lizundia et al. showed that in polylactide (PLA)‐grafted CNCs, the onset of thermal degradation was shifted to lower temperatures compared to neat CNCs or composites formed by simple mixing.^[^ ^111^ ^]^ This further emphasizes the importance of inter‐chain packing in the thermal stability of nanocelluloses. This information can be used to design transient devices with adjustable thermal stability. For example, in devices that maintain low temperatures during normal operation, the nanocellulosic components could be adjusted for lower thermal stability to facilitate less energy‐intensive transiency after EoL.

While thermal degradation may at first seem like a relatively simple transiency mechanism, it is important to consider that the by‐products from this process can be further upcycled for advanced applications or be recycled for other uses. For example, the carbon obtained upon thermodegradation could be used as electrode materials for supercapacitors or batteries,^[^ ^112^ ^]^ or as bionutrients for environmental remediation,^[^ ^113^ ^]^ enabling the further production of renewable transient device precursors.

### 2.7 Summary of Transiency Mechanisms and Degradation Products for (Nano)Cellulosic Materials

Evidently, the realization of cellulose‐based transient devices incorporating a balance of transiency, performance, and cost‐effectiveness may require concurrent application of multiple transiency mechanisms. Each mechanism has its advantages and disadvantages. For example, while redispersion may enable good recovery of nanocellulosic material without the use of specialized solvents, ultrasonication‐based methods may require significant active processing. In some respects, enzymatic degradation may be the most suitable “standalone” method; for instance, one can envision an electronic device incorporating an electrolyte amenable to the activity of cellulases contained within. The requirement in many cases to combine degradation mechanisms to achieve a functional transient device is not inherently a weakness, however. On the contrary, this enables the designing of specific, triggered degradation mechanisms that have the potential to prolong a device's lifetime.

**Figure 9:**
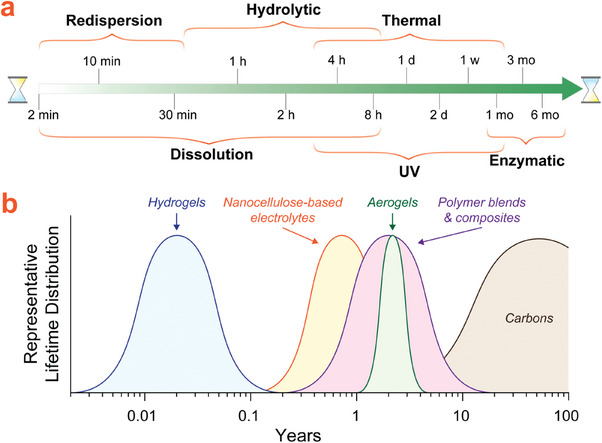
a) Estimated time ranges for achieving cellulose degradation using the transiency mechanisms discussed in this section. min = minutes; h = hours; d = days; w = weeks, mo = months. b) Estimated representative lifetime (from production until the end of useful life; i.e. the point at which degradation is so significant that the material cannot serve its original function) distributions of various types of nanocellulose‐derived transient materials.

When considering device lifetime, there are several critical points to address: the relative times required for each transiency mechanism to act, as well as how device lifespan is defined. For a passively transient device, which necessarily undergoes performance loss during operation, the device lifespan can be defined by the point where acceptable device performance is no longer possible due to significant degradation. On the other hand, transient devices with triggered degradation can show flexible lifespan definitions, as trigger application can be controlled by the user, and be applied after the device is no longer needed. Triggered transient devices with built‐in mechanisms regulated by an internal feedback loop can have their lifespan programmed during the manufacturing process. The time required for each transiency mechanism to act must also be considered, as passively transient devices should be designed to degrade slowly, while triggered transiency mechanisms can be designed to act much more quickly since degradation does not begin without input. Each transiency mechanism is therefore appropriate for a different application due to their varying degradation timescales. While these exact times are highly dependent on other variables in the device design such as surface area or degree of crystallinity, a general hierarchy of degradation times may be discerned based on basic, isolated testing conditions as shown in **Figure** **9**a. More specifically, acid hydrolysis,^[^ ^114^, ^115^ ^]^ dissolution,^[^ ^116^, ^117^, ^118^, ^119^ ^]^ and redispersion (sonication)^[^ ^120^, ^121^, ^122^ ^]^‐based transiency mechanisms can potentially act quickly once triggered, on a timescale of minutes to hours. In contrast, thermal (depending on the heating rate, desired degree of degradation, and desired end use for degradation byproducts),^[^ ^18^, ^105^, ^109^ ^]^ enzymatic,^[^ ^90^, ^96^, ^97^ ^]^ and UV‐induced degradation^[^ ^73^, ^77^ ^]^ may require longer processing times. In turn, different types of nanocellulose‐based materials have varying lifetimes, another factor to consider during transient device design (Figure 9b).

**Figure 10:**
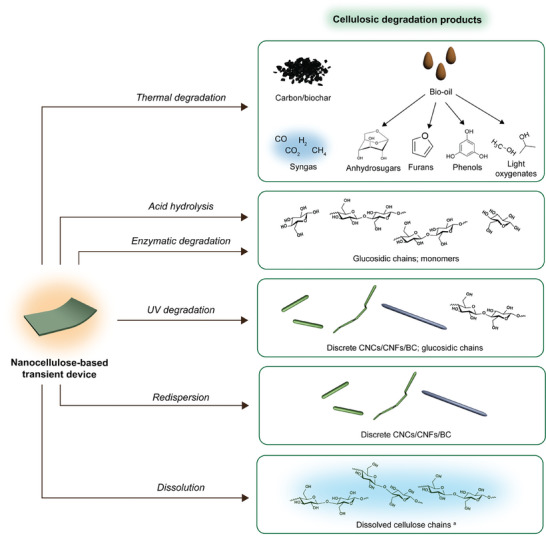
Summary of degradation products arising from the breakdown of (nano)cellulosic transient devices. ^a^ Dissolved cellulose chains may or may not be derivatized depending on the solvent system employed for dissolution; a non‐derivatized representation is given here.

Furthermore, the byproducts of nanocellulose degradation can vary greatly (**Figure** **10**). Considering the full life cycle of a device is crucial for the circular integration of transient materials and devices. It is important to design materials in a way that allows byproducts to serve as precursor materials for further transient devices; otherwise, byproducts can be used as nutrients for the surrounding environment, contributing to the natural growth of more bio‐based precursors. Furthermore, it is important to consider the toxicity of degradation byproducts when designing devices and choose appropriate transiency mechanisms to minimize or avoid forming harmful degradation products. For example, the thermal degradation of (nano)cellulose can produce bio‐oils containing toxic furans and phenolic compounds,^[^ ^123^ ^]^ so the formation of biochar over bio‐oils should be prioritized when designing for thermal transiency. Surface modification of nanocelluloses, as will be discussed in Section 3.4, is an extremely useful tool for transient device development, but may alter the toxicity profile of the resulting material in a negative way, necessitating detailed consideration of these aspects during device design.

## 3 Advantages of Nanocelluloses in Transient Device Design

**Figure 11:**
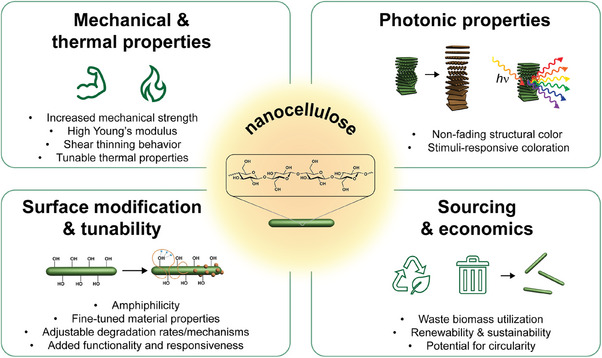
Overview of nanocellulose‐specific advantages in the design of transient devices.

Native cellulose is already an attractive material for the design of functional materials. Cellulose shows great promise when combined with additives such as activated carbon to improve electrical or thermal conductivity, with salts to enable ionic conductivity, or with polymeric materials to improve elasticity in composite elastomers. Its potential sustainability, desirable properties, and abundance permit its use for a wide range of applications. Thus, the question arises: what advantages do nanocelluloses have over bulk cellulose (and other common (bio)degradable polymers) for transient devices? Benefits of nanocelluloses include their mechanical strength, remarkable photonic properties, amphiphilicity, shear thinning character in aqueous suspensions (at concentrations of 1.5–5 wt%) enabling additive manufacturing by 2D or 3D printing, and their relative ease of functionalization compared to bulk cellulose to fit a wide range of potential uses (**Figure** **11**). In this section, the attractive properties that push the potential of nanocelluloses beyond that of other materials are discussed in detail, with specific consideration to the impact of these properties on transient device design.

### 3.1 Mechanical Properties of Nanocelluloses

**Figure 12:**
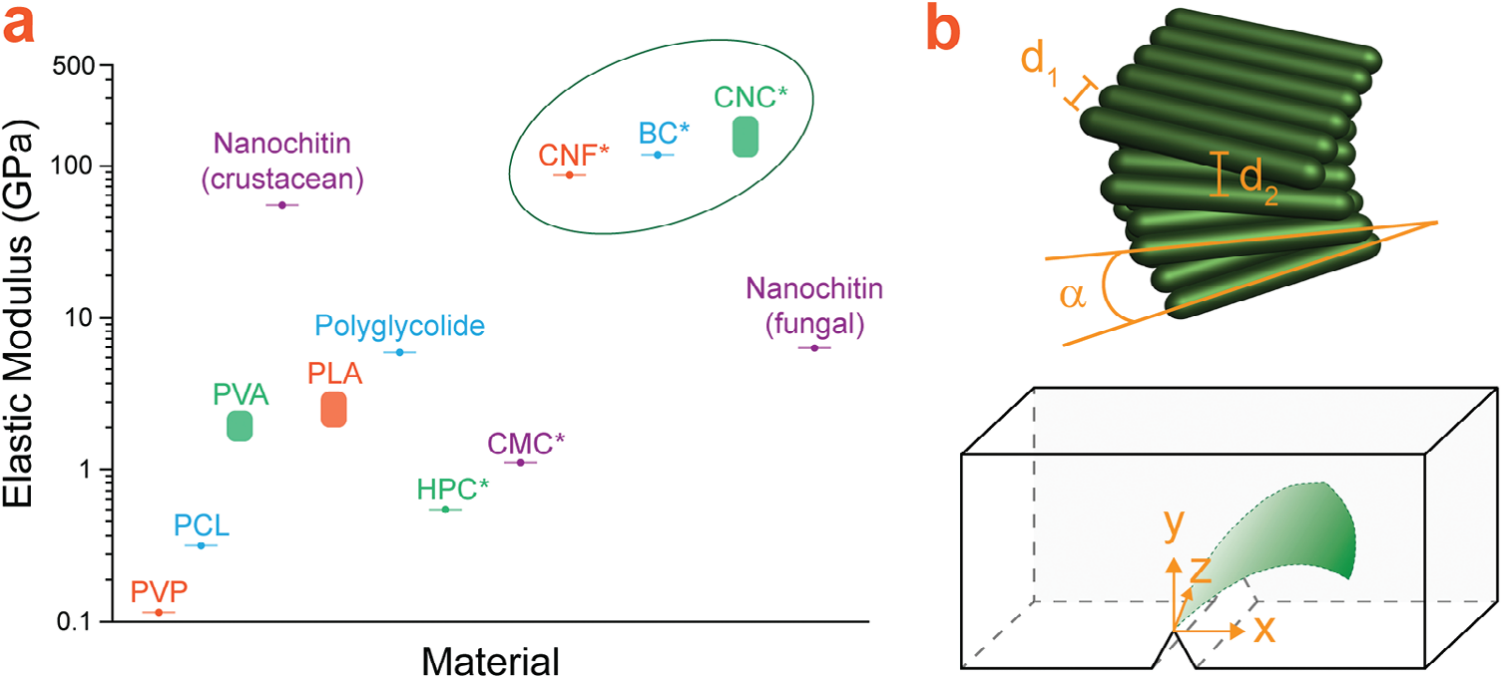
a) Comparison of the elastic moduli for several types of nanocellulose derivatives (circled for emphasis), as well as other degradable polymers commonly used in transient device design. Data are taken from references shown in Table 3. Where only a single modulus value was reported, the value is represented by a point in the graph; vertical bars represent the data range. *cellulose derivatives. b) Upper: Illustration of the Bouligand structure of self‐assembled CNCs, showing the interfiber distances (d_1_, d_2_), and the characteristic helical twisting angle (α). Lower: Crack deflection and twisting in the Bouligand structure leading to increased mechanical strength. The crack propagation direction is represented by *x*, the deflection height of the crack by *y*, and the width of the crack by *z*, parallel to the crack front. Reproduced with permission from.^[^ ^135^ ^]^ Copyright 2021, IOP Publishing.

Nanocellulosic materials have excellent mechanical properties compared to conventional degradable polymers applied in transient devices, as shown in **Figure** **12**a. With an increased storage modulus relative to native cellulose and theoretically Kevlar‐like axial Young's modulus (167.5 GPa), nanocelluloses are attractive as additives in various composite materials.^[^ ^13^ ^]^ Their surface chemistry can also be tuned^[^ ^124^ ^]^ to optimize compatibility with the other components of the composite.^[^ ^13^, ^125^ ^]^ For example, covalent crosslinking between CNCs and polyurethane has been exploited by Pei et al. to achieve effective dispersion, resulting in an eightfold increase in tensile strength with only 1 wt% of CNCs.^[^ ^126^ ^]^ Even on their own, nanocelluloses boast impressive mechanical properties that can be easily tuned during manufacturing.^[^ ^127^ ^]^ Using unmodified and TEMPO‐oxidized CNFs, Sehaqui et al. prepared nanopapers by three drying methods: liquid CO_2_ evaporation, supercritical CO_2_ drying, and *tert*‐butanol freeze‐drying.^[^ ^128^ ^]^ They found that using TEMPO‐oxidized CNFs produced nanopapers with modulus, tensile strength, and strain to failure of 1.4 GPa, 84 MPa, and 17%, respectively, comparable to commodity thermoplastics but with a much lower material density. For both unmodified and TEMPO‐oxidized CNFs, lower porosity was associated with higher modulus values, which the authors attributed to a higher degree of inter‐fiber interactions and agglomerated structure for samples freeze‐dried with *tert*‐butanol.^[^ ^128^ ^]^ Additionally, the mechanical properties of CNC‐based materials can be further enhanced owing to their intrinsic tendency to form films with helical Bouligand‐like structures,^[^ ^129^, ^130^, ^131^ ^]^ which provides increased mechanical strength and impact resistance through crack twisting/deflection and fiber bridging (Figure 12b).^[^ ^132^ ^]^ This effect has been used in materials such as non‐cellulosic ceramic films^[^ ^133^ ^]^ and CNC aerogels^[^ ^134^ ^]^ to offer improved overall mechanical performance^[^ ^133^ ^]^ and asymmetric mechanical and thermal properties.^[^ ^134^ ^]^

The Bouligand structure is difficult to exploit in composite materials, but nanocelluloses can still offer significant mechanical benefits as additives. They have been employed in electrically conductive composites, often to enable material flexibility. For example, Dang and Seppälä reported a conductive nanopaper based on CNFs and reduced graphene oxide (rGO) in which the mixing of these two components led to a 1.8‐fold increase in Young's modulus over neat CNF papers due to the efficient and homogenous mixing enabled by hydrogen bond formation between the two components.^[^ ^136^ ^]^ Akhlaghi et al. mixed BC into cement mortar, and found that the addition of only 0.5 wt% BC increased flexural strength by 104%. Furthermore, coating incorporated polypropylene (PP) fibers with BC allowed for improved integration into the cement matrix.^[^ ^137^ ^]^ Also using CNFs, Kuo et al. found that their incorporation into hybrid epoxy resins at 25 wt% increased tensile strength by 88% and modulus by 298%, and eliminated the need to include toxic bisphenol A in the epoxy formulation.^[^ ^138^ ^]^ Nanocelluloses can also be combined with other polysaccharides for improved mechanical performance, as shown by Dehnad et al. who employed CNCs as an additive in chitosan films to increase elastic modulus and elongation at break, while decreasing water solubility.^[^ ^139^ ^]^

The use of nanocelluloses as additives in composite materials to impart a mechanical advantage is promising. Taking advantage of their intrinsic strength and self‐toughening enabled by self‐assembly, nanocelluloses can be used to fine‐tune mechanical properties for a wide variety of desired applications. Furthermore, the range of mechanical properties that can be varied simply based on nanocellulose aspect ratio and geometry (and therefore source and processing conditions) allows for control over the mechanical characteristics of the resulting nanocellulose‐based materials.

### 3.2 Thermal Properties of (Nano)Celluloses

Cellulose is not known for being especially thermally stable, but its use in transient devices can enable a high degree of control over thermal degradation.^[^ ^18^ ^]^ Bulk cellulose begins to show the onset of thermal degradation at relatively low temperatures, and reaches peak degradation ≈350 °C as the glycosidic bonds become more reactive.^[^ ^18^ ^]^ The thermal stability of nanocelluloses is more complex, where factors such as the degree of surface functionalization and crystallinity can come into play.^[^ ^18^ ^]^ For example, CNCs and CNFs are often decorated with sulfate half‐ester groups, that shift the onset of thermal degradation to lower temperatures.^[^ ^18^, ^140^ ^]^ CNFs can also suffer further from decreased thermal stability due to their amorphous content, but this drawback can be offset by the use of milder processing conditions during production, resulting in a lower degree of surface functionalization compared to CNCs.^[^ ^18^, ^105^ ^]^ Therefore, CNFs are generally more thermally stable than CNCs, but the increased surface area and functionalization potential of CNCs allow for greater tunability of thermal properties.^[^ ^18^, ^105^, ^141^ ^]^ Despite their apparent thermal disadvantages, with careful optimization nanocelluloses can become useful for achieving highly tunable thermal properties, both on their own and as part of composite materials.

CNCs have been included in their native form in composites with polymers such as PVA^[^ ^142^ ^]^ and PLA^[^ ^143^ ^]^ where they have been shown to increase the onset temperature of thermal degradation and melting temperature, respectively. Modified CNCs have also been used in composites to tune thermal stability: Yin et al. demonstrated the combination of polystyrene (PS)‐grafted CNCs with poly(methyl methacrylate) to produce composites with delayed thermal degradation onset compared to unmodified CNCs.^[^ ^144^ ^]^ Of course, CNFs are also used as additives and have been shown to increase thermal stability in some cases, as with Zhang et al.’s demonstration of 15 wt% CNF/thermoplastic starch composites that showed an increase in onset and final thermal degradation temperatures of ≈15 °C compared to thermoplastic starch alone.^[^ ^145^ ^]^

(Nano)celluloses are also increasingly employed for thermal insulation, particularly as porous aerogels or foams.^[^ ^146^, ^147^, ^148^ ^]^ In these cases, thermal conductivity can be tuned simply by controlling the alignment of nanocellulose within the material. For example, Li et al. have demonstrated that the chiral nematic organization of CNCs can be exploited to develop cellulose‐only aerogels with anisotropic thermal conductivity. Due to the organization of CNC chiral nematic layers, these aerogels develop “channels” with orientation‐based differential thermal conductivity.^[^ ^134^ ^]^ In another work, Li et al. fabricated thermally insulating “nanowood” removing lignin and hemicellulose from natural wood to create a highly ordered network of cellulose fibers.^[^ ^149^ ^]^ Due to this anisotropic fiber arrangement, the nanowood displaces heat along the fiber plane direction, inhibiting heat dissipation perpendicular to the layers. This manifested in a thermal conductivity of 0.056 ± 0.004 W∙m^−1^∙K^−1^ in the axial direction, but only 0.032 ± 0.002 W∙m^−1^∙K^−1^ in the radial direction. Thermally insulating nanocellulose‐containing composites have also been explored. For example, Wicklein et al. demonstrated the fabrication of anisotropic foams comprised of CNFs, graphene oxide, and sepiolite nanorods through a directional freeze‐drying method.^[^ ^150^ ^]^ The resulting foams contain aligned macroporous channels, with smaller mesopores within the channel walls. In the axial direction, these materials exhibit a thermal conductivity of ≈0.17 W∙m^−1^∙K^−1^, but in the radial direction this value plummets to ≈0.015 W∙m^−1^∙K^−1^, due both to the anisotropic arrangement and poor inter‐channel connectivity, as well as phonon scattering afforded by the included graphene oxide.

The tunability of nanocellulose thermal properties makes them an interesting option for materials with specific compatibility and performance requirements. This can be exploited for devices employing thermal transiency, to ensure efficient, low‐energy transience and the production of useful carbonaceous degradation products for upcycling. Furthermore, their use in environmentally friendly and circular thermal insulation materials shows great promise, as thermal conductivity properties can be manipulated through tuning nanocrystal/nanofiber arrangements in bulk materials.

### 3.3 (Nano)Cellulose Photonic Properties and Structural Color

**Figure 13:**
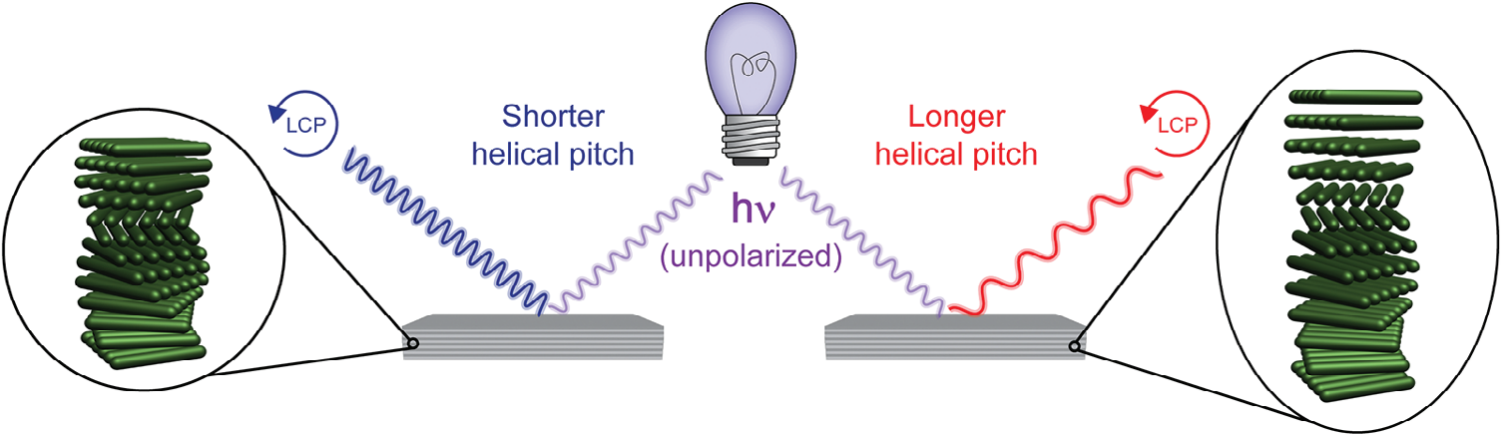
When a chiral nematic CNC‐containing substrate is irradiated with unpolarized light, left‐handed circular polarized light is reflected. The wavelength reflected depends on the helical pitch of the structure.

Aqueous suspensions of CNCs are known to form left‐handed chiral nematic (cholesteric) lyotropic liquid crystalline phases,^[^ ^13^ ^]^ and films of CNCs can retain this helical Bouligand‐like structure.^[^ ^129^, ^130^ ^]^ When the pitch of the helical structures is on the order of the wavelength of visible light, selective reflection of left‐handed circularly polarized light occurs, resulting in structural color (**Figure** **13**).^[^ ^13^, ^151^, ^152^, ^153^ ^]^ This behavior has been harnessed in pristine cellulosic materials,^[^ ^154^ ^]^ and used as a template to impart structural color to other materials such as silica.^[^ ^155^ ^]^ Interestingly, this behavior can also be preserved upon incorporation of CNCs into composite materials. Simple composites of CNCs with polymers such as hydroxypropyl cellulose (HPC),^[^ ^156^ ^]^ poly(ethylene glycol) (PEG),^[^ ^157^ ^]^ and polyvinylpyrrolidone^[^ ^158^ ^]^ have been extensively reported; in such materials the polymeric component generally serves to both enhance the mechanical properties^[^ ^156^, ^157^ ^]^ of the material and allow for tuning of CNC organization and resulting structural color.^[^ ^158^ ^]^

Functional composite materials that actively make use of the photonic properties of chiral nematic CNCs have also been realized. The affinity of CNCs to water has enabled the development of humidity‐responsive composites, in which the reversible swelling associated with water absorption causes an increase in helical pitch and subsequent bathochromic shift in structural color.^[^ ^157^, ^159^, ^160^ ^]^ Additives in such composites often include polymers such as PEG or PVA^[^ ^160^ ^]^, or plasticizers such as glucose or glycan^[^ ^159^ ^]^ which increase the capacity for water absorption. Thermally responsive photonic composites can also be fabricated; in these materials, thermal responsiveness is achieved through swelling or conformational change of the polymer component in response to heat, or phase transition of liquid crystals incorporated in the CNC matrix.^[^ ^161^ ^]^ In the former case, the mechanism is similar to that of humidity‐responsive materials in that the conformational change of the polymer induces a change in chiral nematic pitch of CNCs.^[^ ^162^, ^163^ ^]^ In the latter case, temperature‐induced phase changes of incorporated liquid crystals result in a shifted refractive index for the composite, altering the observed structural color.^[^ ^161^ ^]^

Mechanical deformation is the most common actuation mechanism for responsive photonic CNC‐based composites.^[^ ^161^ ^]^ CNCs can be captured in elastomeric materials, such as the acrylate/CNC composites reported by Boott et al.^[^ ^164^ ^]^ In these materials, stretching of the material perpendicular to the CNC chiral nematic axis shrinks the helical pitch, resulting in a hypsochromic shift of the structural color. He et al. reported a similar composite material, simply including glycerol as a plasticizer to produce flexible films that showed a hypsochromic color change upon compression, but the lack of an elastomeric network hindered reversibility.^[^ ^165^ ^]^ Many examples of similar materials have been shown in the literature, with development even extending to pressure‐ and heat‐sensitive shape‐memory polymers based on CNCs.^[^ ^166^, ^167^ ^]^

Clearly, the main advantage of using CNCs to achieve photonic composites from a transient materials design perspective is the simplicity – eliminating the requirement to include potentially toxic components or inorganic materials to achieve photonic responsiveness allows for a streamlined approach to designing the degradation pathway of CNC‐based photonic composites. Furthermore, CNC structural color does not degrade over time, providing enhanced durability over dye‐containing materials, which may experience a gradual color fade as the material ages.

### 3.4 Tunability of Cellulose Functionality through Surface Modification

**Figure 14:**
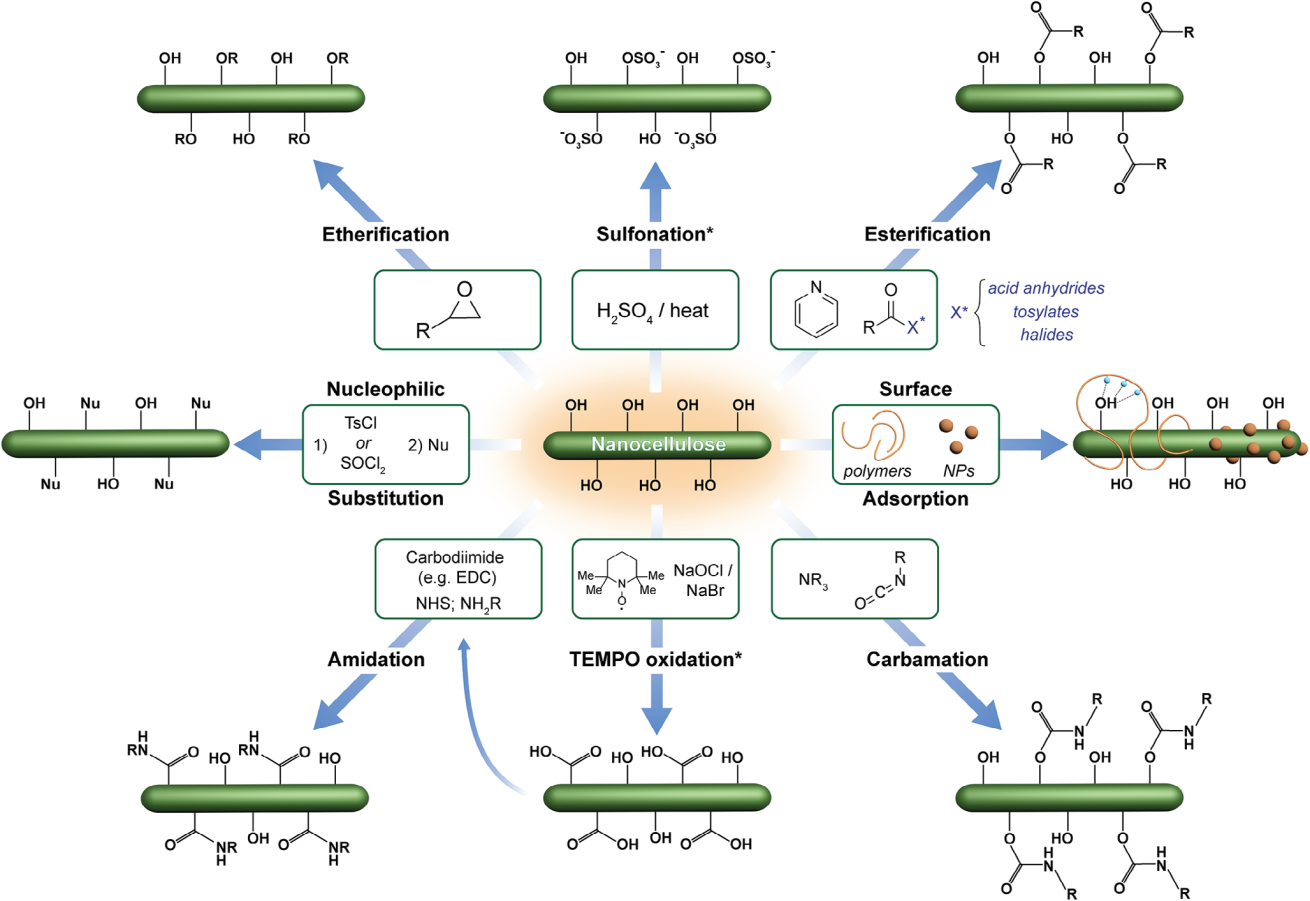
Nanocellulose surface modifications are discussed in this section. Nanocelluloses are shown only partially functionalized at the surface to reflect the incomplete modifications achieved by most techniques. For each modification technique, a simplified common example of processing conditions is shown; research into nanocellulose surface modification is diverse and many other possible conditions exist.

Perhaps one of the most important advantages of nanocellulose is the tunability of its surface chemistry. Some of the most typical surface modifications include carboxylation, esterification, carbamation, amidation, etherification/nucleophilic substitution, and even non‐covalent surface adsorption of macromolecules or inorganic nanoparticles (**Figure** **14**).^[^ ^168^ ^]^ Furthermore, small molecules, macromolecular moieties, and bioactive proteins/enzymes can be anchored onto nanocellulose surfaces utilizing grafting to, grafting from, and grafting through approaches.^[^ ^169^ ^]^ In this section, methods for nanocellulose surface modification are elaborated, and potential use cases and implications for the implementation of transiency for each method are discussed to highlight the versatility of nanocelluloses.

Aside from acid‐catalyzed hydrolysis, TEMPO oxidation is the most common method to produce CNCs, especially CNFs. TEMPO ((2,2,6,6‐tetramethylpiperidin‐1‐yl)oxidanyl) is a stable organic free radical that, when combined with NaOCl and NaBr, catalyzes the conversion of cellulose C6 hydroxyl groups to carboxyl groups.^[^ ^170^ ^]^ This technique is often combined with ultrasonication^[^ ^171^ ^]^ or mechanical fibrillation^[^ ^172^ ^]^ to aid in the conversion to nanocellulose. These techniques can increase yields,^[^ ^173^ ^]^ lower reaction times, and decrease the degree of polymerization (DP) of the resulting nanocellulose. For example, TEMPO‐oxidized nanocelluloses have been used to prepare water‐absorbing shape‐memory aerogel materials^[^ ^174^ ^]^ and lignin‐containing nanocelluloses with antioxidant and antibacterial properties.^[^ ^175^ ^]^

Esterification of nanocelluloses is another simple method for surface modification. Similar to TEMPO oxidation, it can be performed concurrently with the hydrolysis of bulk cellulose by using a mixture of organic acid (e.g. citric, malic, or malonic acid) and HCl as a co‐catalyst to achieve one‐step production of nanocelluloses decorated with diverse carboxylic acid moieties.^[^ ^176^ ^]^ Esterification is not limited to organic acids, however. By employing the exposed (usually C6) hydroxyl groups of CNCs as nucleophiles in transesterification reactions, a wide variety of functionalities can be added to the CNC surface through reactions with acyl chlorides or anhydrides.^[^ ^177^ ^]^ For example, He et al. reported the esterification of CNCs with acetic, caproic, and butyric anhydrides; with this modification, they were able to create CNCs that were more miscible with non‐polar polymer matrices to increase their usefulness in composite materials.^[^ ^178^ ^]^

Amidation of nanocelluloses generally begins with TEMPO‐oxidized, carboxylated CNCs or CNFs.^[^ ^168^ ^]^ From this starting point, conditions similar to peptide coupling reactions (e.g. combinations of *N*‐hydroxysuccinimide (NHS) and carbodiimides) can be used to activate the carboxyl groups through NHS‐ester formation, followed by a reaction with a primary amine to form the desired amide product.^[^ ^168^ ^]^ As an example, Krathumkhet et al. modified CNCs with octadecylamine to produce flexible films with oil‐repellent properties; they suggested that such materials could be used in anti‐fingerprint or anti‐fouling applications.^[^ ^179^ ^]^ On the other hand, carbamation is often employed through the reaction of nanocellulose surface hydroxyl groups with isocyanate‐containing molecules or polymers.^[^ ^168^ ^]^ Carneiro de Oliveira et al. used this technique to graft Diels–Alder capable functional groups onto CNCs through the use of toluene diisocyanate (TDI), potentially enabling future complex organic modifications to the CNC surface.^[^ ^180^ ^]^ Carbamation is also useful for increasing the dispersion of nanocelluloses as additives in polymer matrices, as shown by Olonisakin et al. who modified MCC with TDI to enable efficient dispersion into a PLA matrix through the formation of urethane crosslinks between adjacent cellulose chains.^[^ ^181^ ^]^

While the methods discussed thus far are the most common and accessible, other less frequently employed methods exist. For example, nucleophilic substitution and etherification‐type modifications operate similarly, both exploiting the nucleophilicity of the accessible surface hydroxyl groups on nanocelluloses.^[^ ^168^ ^]^ These techniques can be used to reverse the surface charge of nanocelluloses through the introduction of cationic (often ammonium) groups,^[^ ^168^, ^182^ ^]^ which in turn affords opportunities for the fabrication of complex nanocellulose conjugates, such as polymeric SiRNA complexes for drug delivery^[^ ^183^ ^]^ or the grafting of macrocycles such as cyclodextrins to enable the formation of complex CNC‐tethered poly(pseudo)rotaxanes.^[^ ^184^ ^]^ Extensive research has even been conducted using fluorophore‐grafted CNCs as probes for bioimaging,^[^ ^185^, ^186^, ^187^, ^188^ ^]^ an exciting application that exploits the potential biocompatibility of nanocelluloses.^[^ ^189^ ^]^

In contrast to covalent modification strategies, simple surface adsorption due to hydrogen bonding or other interactions may also occur and can be sufficient to significantly alter the material properties.^[^ ^158^ ^]^ For example, the incorporation of iron oxide (Fe_3_O_4_) nanoparticles on the surface of CNCs has been shown to enable magnetic control over their cholesteric self‐assembly,^[^ ^190^ ^]^ or use as a composite electrode material for the detection of medicines in wastewater.^[^ ^191^ ^]^ Decoration with Au nanoparticles enables the use of surface‐enhanced Raman spectroscopy (SERS)‐based analysis techniques, as shown by Rahman et al. where Au nanoparticle‐coated BC was used for the detection of 19 bacterial strains with high accuracy.^[^ ^192^ ^]^ Numerous other composite materials have been created using inorganic nanoparticles such as ZnO, Fe_2_O_3_, TiO_2_, and Ag for water purification applications, enabling the removal of harmful organic dyes or heavy metals.^[^ ^193^ ^]^

Along with affecting the chemical properties, surface modification can also play a role in transient degradation behavior, where the DS can significantly influence the degradation rate. For example, faster thermal degradation rates can be observed for higher DS nanocelluloses.^[^ ^88^ ^]^ In general, this occurs simply due to the increased number of degradation pathways available at the surface of the material. This behavior has been observed in CNCs grafted with aliphatic amines (C8‐C16)^[^ ^194^ ^]^ and biodegradable polymers such as PLA,^[^ ^195^ ^]^ in which the initial degradation of the grafted species induces a cascade of degradation in the bulk material at an earlier onset temperature than unmodified CNCs. Passive transiency mechanisms can also be affected by surface modification. For example, introduction of dialdehyde groups on the surface of cellulose via periodate oxidation has been shown to speed up the rate of passive dissolutive degradation at pH 7.^[^ ^196^ ^]^ Evidently, surface modification provides potential avenues to accelerate nanocellulose degradation in selected environmental conditions.

Academia and industry working with a traditional materials design philosophy in mind are more apt to pursue modifications that increase the resistance of nanocelluloses or nanocellulose‐based composites to degradation under working conditions. In this context, the use of phosphoric acid instead of sulfuric acid or TEMPO‐oxidation during CNC extraction can improve resistance to thermal degradation; phosphorylated CNCs have been shown to be significantly more resistant to thermal degradation than their sulfonated or carboxylated counterparts.^[^ ^195^ ^]^ When esterification is employed for surface modification, interesting trends begin to emerge. Agustin et al. demonstrated that modification of BC with some esters lacking α‐hydrogens (benzoyl and pivaloyl) reduced the rate of thermally‐induced degradation compared to α‐hydrogen‐containing esters (acetyl and myristoyl).^[^ ^197^ ^]^ While they did not generalize this effect to other materials, these results further reinforce the complexity of nanocellulose‐surface group interactions when considering degradation. In composite materials containing both nanocelluloses and other (bio)degradable polymers, thermal stability is often enhanced through the addition of “compatibilizers”, i.e. groups that facilitate improved interactions between the nanocellulose and polymer matrix (e.g. phthalic anhydride).^[^ ^195^, ^198^ ^]^ Finally, as discussed in Section 2.5, surface modifications can also have a drastic effect on the efficiency of enzymatic degradation of nanocelluloses. In fact, enzymatic hydrolysis has been shown to be hampered significantly by most surface modifications, presumably due to reduced fit quality of the specific cellulose‐binding domains of cellulases.^[^ ^101^, ^102^ ^]^ Glutaraldehyde crosslinking of methyl cellulose has been shown to slow down degradation processes in soil,^[^ ^199^ ^]^ while hexamethylenediamine crosslinking of TEMPO‐oxidized CNFs accelerates enzymatic degradation due to the nitrogen in the crosslinker boosting degradation events.^[^ ^96^ ^]^

As mentioned, literature reports on the degradation of nanocelluloses and nanocellulose‐containing composites mainly focus on increasing their stability. However, there are a multitude of research works describing stimuli‐responsive materials crafted from chemically‐modified nanocelluloses.^[^ ^200^ ^]^ While many of these have not been explored for the express purpose of tuning degradation behavior, one can imagine employing stimuli‐responsive surface modifications to precisely adjust transient device properties. For example, thermoresponsive nanocellulose‐based systems have been made by grafting polymers with tunable lower critical solution temperatures onto CNCs, resulting in composites that exhibit heat‐triggered gelation^[^ ^201^ ^]^ or stiffening.^[^ ^202^ ^]^ pH‐responsive systems have also been demonstrated, especially for drug delivery applications. Often taking the form of pH‐sensitive injectable hydrogels, composites containing cellulose derivatives such as CMC have been employed to create materials with pH‐triggered release of cancer drugs such as doxorubicin, that can also degrade in biological environments on a timescale of days to several months.^[^ ^203^, ^204^ ^]^ While in these reports the impact of surface modification on degradation often takes a back seat to the functionality of the material, the potential for tuning surface‐modified nanocellulose systems to achieve precise transient behavior is clear.

Overall, the tunability of nanocelluloses is impressive in comparison to bulk cellulose. To provide a bigger picture, representative examples of surface modification and their implications on degradation (if reported) are tabulated in **Table** **2**. Even having only scratched the surface here so far, it is evident that the possibilities for modification to suit almost any desired application place nanocelluloses in a very exciting and favorable position. The extensive processing required to realize these modifications may be prohibitive for some applications, but with the continuing development of more scalable processing methods, nanocelluloses are well‐situated to act as additives for specific transient functional materials. We also stress that the altered degradation products of modified nanocelluloses may require extensive characterization and screening for environmental and human toxicity. Transient devices must be designed to degrade harmlessly into the environment, so even if increased biodegradability is achieved using a given nanocellulose modification, the production of more toxic degradation products is antithetical to the design philosophy of transient materials.

**Table 2:** Summary of examples of (nano)cellulose surface modifications, their diverse suite of potential applications, and (if reported by the authors) the implication of each modification type on the material transiency.

Material & modification	Application	Impact of modification	Implications for transiency^a)^	Refs.
TEMPO‐oxidized CNFs	CNF aerogels (99.5% porosity) for high performance water absorption and shape memory	Increased water compatibility (similar to water‐soluble polymers)	–	[174]
TEMPO‐oxidized lignin‐containing nanocelluloses; Amidation with PEI^b)^	Antibacterial and antioxidant composites	Comparable antibacterial and antioxidant properties to free PEI, at lower concentration	–	[175]
CNCs; Esterification with C2, C4, or C6 aliphatic chains	Hydrophobic CNC composites with poly(butylene succinate)	Improved thermal stability (for C4 and C6 modifications); Improved dispersibility in hydrophobic polymer matrix (for C4 modification); Improved composite tensile modulus (for C4 modification)	–	[178]
CNCs; Esterification + Schiff base reaction with FITC^b)^ dye	Bioimaging & material reinforcement	>30% fluorescence QY as a bioimaging probe; Biocompatibility (mouse osteoblasts); Improved mechanical properties of PVA and PLA composites	–	[185]
BC; Esterification with acetyl, benzoyl, pivaloyl, and myristoyl chloride	Improving thermal resistance of nanocellulose	Thermal resistance highest when modifications lacking α‐hydrogens (benzoyl & pivaloyl)	Control over thermal depolymerization rate to slow degradation	[197]
CNC; Esterification with acetic anhydride	Improve phase blending in composite PBAT**/CNC composite sheets	Increased elastic modulus; Improved thermal stability of composite	Control over thermal degradation in nanocellulose‐containing composites	[198]
Methyl cellulose; Esterification (crosslinking) with glutaraldehyde	Modulation of thermal properties, moisture absorption, and biodegradation in methyl cellulose composites	Decreased moisture absorption with higher crosslinking; Maximized tensile modulus with moderate (5 wt%) crosslinker; Decreased biodegradability when crosslinked	Tunability of enzymatic degradation rate with crosslinking density, due to change in water transport rate through the composite	[199]
CMC; Esterification with carboxylated graphene quantum dots	Anticancer oral drug delivery with pH responsiveness	pH‐sensitive swelling (higher at pH 7.4 vs pH 2); Improved tensile strength, decreased flexibility; pH‐responsive release of doxorubicin; pH‐and quantum dot concentration‐dependent biodegradation rate	Diverse property tuning with simple variation of quantum dot concentration; a “sweet spot” of biodegradation, drug release, and mechanical properties can be found for any application	[203]
TEMPO‐oxidized CNFs; Amidation with octadecylamine	Non‐fluorinated oil‐repellent films for antifingerprint applications	Increased elongation at break, decreased tensile strength and modulus; Increased hydrophobicity; Oil repellency (at moderate degrees of modification)	–	[179]
TEMPO‐oxidized BC; Amidation with lectin; Adsorbed Au NPs	Selective bacterial detection through SERS	Lectin aids to form homogenous network; AuNPs enable position‐sensitive SERS measurement of 19 bacterial strains	–	[192]
TEMPO‐oxidized CNFs; Amidation (crosslinking) with hexamethyldiamine	Nanocellulose‐based superabsorbent polymer	Increased biodegradation in crosslinked materials due to N‐content of crosslinker	Potential to simultaneously improve mechanical properties and increase degradation rate	[96]
CNCs; Carbamation with maleimides	Diels‐Alder‐capable CNCs	Enablement of further complex organic modification on nanocellulose	–	[180]
MCC; Carbamation with PLA	Incorporation of MCC into PLA matrices through covalent bonds	Increased tensile strength and elongation at break compared to unmodified MCC	–	[181]
CNCs; Nucleophilic substitution with CHPTAC^b)^	Anticancer siRNA drug delivery nanocomposites	Reversal of CNC surface charge enables electrostatic complexation with polyanionic siRNA	–	[183]
CNCs; Nucleophilic substitution with epichlorohydrin/β‐cyclodextrin	Supramolecular hydrogels for anticancer drug delivery	β‐cyclodextrin/Pluronic rotaxane formation at CNC surface to produce thixotropic hydrogels	–	[184]
CNCs; Adsorbed Fe_3_O_4_ NPs	Screen‐printed electrodes for electrochemical sensing of venlafaxine (antidepressant)	Detection of venlafaxine concentration through electrooxidation by adsorbed Fe_3_O_4_ NPs	–	[191]
CNCs: Adsorbed Fe_3_O_4_ NPs	Composite aerogels for supercapacitor electrodes	Improved electrochemical performance; Magnetic responsiveness	Accelerated CNC degradation onset through earlier loss in crystallinity	[65]

^a)^ Left blank when not reported in the given reference;

^b)^ PEI: Polyethyleneimine; CHPTAC: 3‐chloro‐2‐hydroxypropyltrimethyl ammonium chloride; FITC: fluorescein isothiocyanate; PBAT: poly(butylene adipate‐*co*‐terephthalate).

### 3.5 Sourcing and Economics of Nanocellulosic Materials

(Nano)cellulosic materials show great potential for integration into the Circular Economy. As a bio‐derived resource, they can be environmentally, economically, and socially sustainable, but only if best practices for processing as well as raw material cultivation and use are followed. Overall, we are witnessing a desire to transition from petroleum‐based polymers to sustainable substitutes across industries,^[^ ^205^ ^]^ but there are several roadblocks to commercial viability and reduction of environmental impact that must be addressed. Thankfully, cellulose‐based materials can help to overcome these challenges through continued innovation and streamlining of processing techniques. We discuss nanocelluloses in the context of sustainability and the Circular Economy in more detail in Section 5.

Nanocelluloses can be derived from numerous feedstocks, such as plants, marine animals, bacteria, and invertebrates.^[^ ^205^ ^]^ This fact allows for potential flexibility in the chain of production; supply chains could be adjusted relatively quickly to make use of whatever feedstocks are most locally abundant at any given time. Importantly, this comes into play when considering the use of “waste” biomass and recycled materials to produce nanocelluloses. For example, Ozola et al. reported in 2019 that 58% of waste paper is recycled worldwide on average, with this value increasing to 70–75% in some countries.^[^ ^206^ ^]^ As the theoretical maximum recycling rate for paper is 78% (taking into account non‐recyclable paper such as coffee filters), there is still ample room for growth in this stream of nanocellulose feedstock.^[^ ^206^ ^]^ Ozola et al. also assessed the viability of recycling waste paper into nanocellulosic materials compared to other paper products, and found that since de‐inking is not necessary, processing into nanocellulosic materials can be cheaper than the current most common methods.^[^ ^206^ ^]^ There is significant room for growth in many agricultural sectors as well. For instance, waste from harvesting palm oil is normally burned, but could potentially be converted into higher‐value nanocellulose materials.^[^ ^207^ ^]^ More generally, Mujtaba et al. reported that although 181.5 billion tons of lignocellulosic agricultural waste are produced annually, only 8.2 billion tons are used, representing a huge missed opportunity for reprocessing.^[^ ^208^ ^]^

This ability to repurpose so‐called “waste” biomass to produce nanocelluloses meshes well with the concept of a Circular Economy. According to the Ellen MacArthur Foundation, in a Circular Economy materials are not simply thrown away; instead, they are either kept in circulation through reuse and refurbishing or cycled into feedstock materials to create new products.^[^ ^209^ ^]^ Nanocelluloses can act at both ends of this cycle, by being produced from waste biomass, and by being broken down (in the form of transient devices) and used as building blocks to create new materials. Of course, achieving this reality also requires more optimization of nanocellulose pre‐processing techniques and supply chains to reduce energy consumption for manufacturing, environmental impact, and the excessive use of process chemicals such as H_2_SO_4_. Advances in this field are ongoing. For example, the low‐cost, low‐energy production of CNFs through mechanical fibrillation alone has been demonstrated, affording the manufacture of CNFs at a total cost of only €1.50 kg^−1^ (compared to €200 kg^−1^ for traditional TEMPO‐oxidized CNFs).^[^ ^210^ ^]^ Environmentally friendly microbial pretreatments for lignocellulosic biomass have also been proposed,^[^ ^211^ ^]^ and methods for improving nanocellulose yields such as microwave‐assisted synthesis, ultrasonic treatment, and mechanochemical activation are in development.^[^ ^205^ ^]^ Additionally, the improvement of acid recovery methods after hydrolytic production of CNCs has led to decreasing costs and more streamlined industrial protocols.^[^ ^212^ ^]^ The economic feasibility and sustainability of a given manufacturing method depends on a large variety of factors such as biomass source, energy costs, or desired use cases for the final product, but even so continuous advances are being made to push (nano)cellulosic materials into mainstream use. Clearly, while the integration of (nano)celluloses into the Circular Economy is not quite complete, they have enormous potential for a more sustainable future. Their abundance, renewability, and continuously improving processing methods offer an optimistic outlook for the extended use of nanocellulose in transient devices.

### 3.6 Nanocelluloses Versus Other Common Biodegradable Polymers

To give a full picture of the benefits provided by nanocelluloses over other (bio)degradable polymers that are currently in use in transient devices, **Table** **3** compares the mechanical and thermal properties, along with water compatibility, possible degradation routes, and estimated carbon footprint. Nanocelluloses generally have higher elastic moduli than many other polymeric alternatives, making them suitable as strengthening additives. Their degradation temperatures rival those of polymers such as PLA or PVA, and the tunability of thermal properties with processing makes them very versatile. Nanocelluloses also carry the advantage of amphiphilicity, making them highly compatible with aqueous systems, and can be readily degraded in water as well at the end of their lifetime. Finally, while the carbon footprints of many nanocellulosic materials currently stand at a higher level than benchmark polymers found in transient devices, a great deal of this disparity can likely be explained by differences in manufacturing process maturity, a point which will be further elaborated in Section 5.

**Table 3:** Overview of the properties of cellulose and other polymeric materials found in transient devices. Note that provided values represent average data, as these properties may significantly change with molecular weight, modification degree, and other parameters.

Material	Mechanical properties	Thermal properties	Distinctive characteristics	Water compatibility	Degradation	Carbon footprint (kg CO_2_ equiv.∙kg^−1^)	Refs.
Cellulose nanocrystals	*E_axial_* = 110–220 GPa	*Td* ≈ 180 °C	Bio‐based Chiral‐nematic Negative charge	Amphiphilic	Dispersible in H_2_O	12.6–178	[213, 214]
Cellulose nanofibers	*E_axial_* = 86 GPa *E_nanopaper_* = 10–21 GPa	*Td* ≈ 250 °C	Bio‐based Negative or neutral charge	Amphiphilic	Dispersible in H_2_O	190–1160	[215, 216, 217]
Bacterial cellulose	*E_axial_* = 114 GPa *E_nanopaper_* = 18 GPa	*Td* ≈ 320 °C	Bio‐based Negative or neutral charge	Amphiphilic	Hard to disperse in H_2_O	16.7	[218, 219, 220]
Hydroxypropyl cellulose	*E* = 0.6 GPa *ɛ_b_* = 13%	*Td* ≈ 260 °C	Thickener Cholesteric liquid	Amphiphilic	Water soluble < 45 °C	3.7 ^a)^	[221, 222]
Carboxymethyl cellulose	*E* = 1.1 GPa *ɛ_b_* < 5%	*Td* ≈ 270 °C	Thickener Good film‐forming	Amphiphilic	Enhanced solubility at 25–40 °C	4.4–10.7	Ecoinvent 3.10, ReCiPe 2016 Midpoint (H) ^[^ ^223^ ^]^
Crustacean nanochitin	*E_axial_* = 60 GPa	*Td* ≈ 300 °C	Bio‐based Chiral nematic Positive charge	Amphiphilic	Dispersible in H_2_O	105–907	[214]
Fungal nanochitin	*E* = 6.9 GPa	*Td* ≈ 280 °C	Bio‐based Slight positive charge	Amphiphilic	Dispersible in H_2_O	18.5	[214, 224]
Polyglycolide	*E* = 6.5 GPa *ɛ_b_* < 10%	*Tg* = 35–40 °C *Tm* = 220–225 °C *Td* ≈ 300 °C	Thermoplastic Bio‐based or fossil‐based	WCA ≈ 50°	90 wt% loss, 50 d (hydrolytic)	N.R. (<10)	[225]
Polylactic acid	*E* = 2.0‐3.5 GPa *ɛ_b_* < 10%	*Tg* = 55–65 °C *Tm* = 170–220 °C *Td* ≈ 260 °C	Thermoplastic Bio‐based or fossil‐based	WCA ≈ 83°	15–25 wt% loss, 180 d (hydrolytic)	2.0–8.0 (petroleum) ≈ 0.5 ^b)^ (bio‐based)	[226]
PCL	*E* = 0.34 GPa *ɛ_b_* = 300–1000%	*Tg* = ‐60 °C *Tm* = 60 °C *Td* ≈ 350 °C	Thermoplastic Fossil‐based	WCA ≈ 88°	3 wt% loss, 770 d (hydrolytic)	N.R. (<10)	[225]
Polyvinyl alcohol	*E* = 1.7‐2.5 GPa *ɛ_b_* ≈ 170%	*Tg* ≈ 50 °C *Tm* = 190–230 °C *Td* ≈ 240 °C	Thermoplastic Fossil‐based	WCA ≈ 51°	Water soluble	2.5–3.5	[227, 228, 229]
Polyvinylpyrrolidone	*E* = 0.12 GPa *ɛ_b_* < 10%	*Tg* ≈ 170 °C *Td* ≈ 370 °C	Amorphous Fossil‐based	WCA ≈ 27°	Water soluble	7.4–35.8	[230, 231, 232, 233, 234]

For the cases where the carbon footprint for certain materials is not reported yet, we provide potential values.

^a)^ = considering methyl cellulose as a model cellulose derivative.^[^ ^235^ ^]^

^b)^ = accounts for biogenic carbon capture.

*E*: Young´s modulus; ɛ_b_: elongation at break; T_g_: glass transition temperature; T_m_: melting temperature; T_d_: thermodegradation temperature; WCA: water contact angle. N. R. not reported.

## 4 Applications of (Nano)Cellulose‐Based Transient Devices

Organic and inorganic materials have both been used to develop transient devices. While inorganic materials often provide enhanced electrical or electrochemical properties, organic materials are in general more mechanically flexible, easier to fabricate, and very importantly, display improved biodegradability/biocompatibility.^[^ ^12^, ^25^ ^]^ In this context, this section focuses on materials of natural origin, in particular (nano)cellulosic materials, which also present clear advantages in terms of sourcing and environmental impact. While many examples of (nano)cellulose‐based transient devices exist in the literature, this section highlights works in which cellulose demonstrates unique properties such as chiral self‐assembly, high water uptake, or enhanced ionic conductivity to enable devices with improved performance when compared to other materials. To enrich the discussion, we also compare examples of some other biobased polymers.

### 4.1 Transient Sensing Devices

In the Internet of Things (IoT) era, with over 50 billion electronic devices simultaneously connected, the short service‐life and single‐use character of consumer electronic devices raise serious environmental concerns.^[^ ^236^ ^]^ The sensing and monitoring of physical parameters such as temperature or humidity are increasingly relevant, especially in green smart‐packaging, agricultural sensing, or point‐of‐care testing applications. Thus, there have been several efforts in recent years to create transient sensing devices.

**Figure 15:**
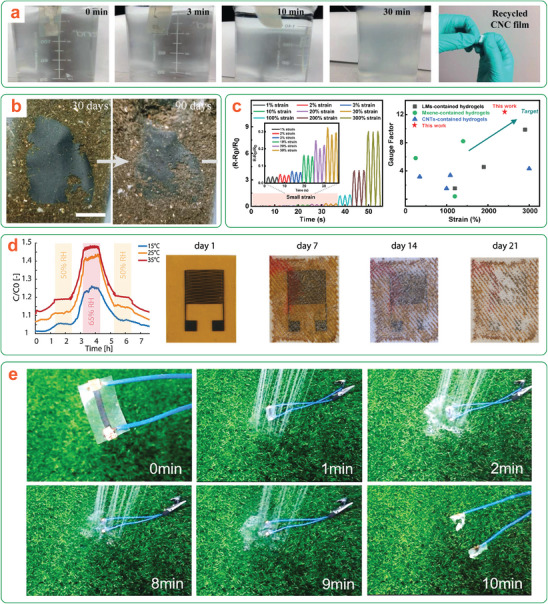
a) CNC/Ag strain sensor in water dissolves in 30 min, and the material is recovered for a new film formation. Reproduced with permission.^[^ ^238^ ^]^ Copyright 2016, Royal Society of Chemistry. b) CNF/MXene hierarchical film degradation when buried in soil (scale bar: 1 cm). Reproduced with permission.^[^ ^239^ ^]^ Copyright 2022, Elsevier. c) Time‐dependent PAA‐CNF‐LMNPs hydrogel relative resistance for different tensile strains. Ashby plot of strain and sensitivity for the state‐of‐the‐art conductive hydrogels. Reproduced with permission.^[^ ^241^ ^]^ Copyright 2022, Elsevier. d) Relative capacitance (C/C_0_) of the sensor as a function of relative humidity and temperature. Aerobic composting disintegration of the capacitive sensor over 21 days. Reproduced with permission.^[^ ^244^ ^]^ Copyright 2023, Wiley‐VCH. e) Sensor degradation under water flush over 10 min. Reproduced with permission.^[^ ^245^ ^]^ Copyright 2020, American Chemical Society.

Strain sensors exploit resistance changes of the material upon shape deformation. As an example, Liu et al. mixed cellulose fibers recovered from waste paper with graphite (55 wt%), and processed the mixture by conventional papermaking techniques to obtain a strain‐sensitive paper.^[^ ^237^ ^]^ A bending gauge factor of 27 was achieved, and the sensor constituents could be disintegrated after only 72 s of stirring and rubbing in water. In other work, a strain sensor with a gauge factor of 52.5 was fabricated on 200 µm thick CNC substrates using stencil lithography.^[^ ^238^ ^]^ A polydimethylsiloxane (PDMS) shadow mask was used to deposit a AgNP electrode onto the CNC substrate; the low surface roughness (3–5 nm) of the CNC film enabled a controlled coating of Ag. Although the brittleness of the CNC films limited the maximum strain range to 1.4%, the Ag electrodes completely peeled off after 10 min, and the entire cellulosic substrate dissolved after only 30 min in water (**Figure** **15**a). The supernatant of the solution could be used for subsequent CNC film fabrication, and the Ag could be recovered by ultracentrifugation, facilitating recycling. In another paper, Ti_3_AlC_2_ MXenes, a 2D transition metal carbide material, were incorporated into nanocellulosic structures to fabricate transient sensors for sitting posture recognition and continuous electrocardiogram monitoring.^[^ ^239^ ^]^ The hierarchical MXene/CNF structure obtained by vacuum filtration combined the mechanical elasticity of CNFs and conductivity of MXenes to offer a wide detection range of 0–950 kPa with a sensitivity of 34.6 kPa^−1^ for more than 3000 cycles. As shown in Figure 15b, the material degraded in soil, and could be transformed into easily recoverable rutile TiO_2_ nanoparticles (oxidation of MXene) and CO, CO_2_, and H_2_O (oxidation of CNFs) through flame ignition.

The detection of volatile organic compounds (VOCs) is critical in applications such as health diagnostics or environmental monitoring. In this context, TEMPO‐oxidized CNFs were assembled with 2D MoS_2_, producing vertically aligned channels for VOC detection.^[^ ^240^ ^]^ 100 ppm of ethanol, methanol, ammonia, or acetone induced a resistive response variation of 18.2‐84.4% (resistance increased due to the electron‐donating character of tested VOCs). Additionally, the materials, including the Au electrode stripes, were completely dissolved after 6 days in phosphate‐buffered saline (PBS) or NaHCO_3_ buffer solution at 75 °C without releasing toxic degradation products.

Sensors based on conductive gels that simultaneously offer mechanical resilience, electrical sensitivity, and rapid transiency remain a challenge. Ye et al. proposed a solution to this issue using hydrogel networks held together with only non‐covalent interactions, i.e. ionic interactions and hydrogen bonding.^[^ ^241^ ^]^ They reported a hydrogel composed of TEMPO‐oxidized CNFs, poly(acrylic acid) (PAA), and embedded 270–475 nm liquid metal nanoparticles (LMNPs) that offered an impressive balance between stretchability (2950%), conductivity (0.45 S·m^−1^) and strain sensitivity (gauge factor of 12.5, Figure 15c).^[^ ^241^ ^]^ The lack of covalent crosslinking enabled rapid transiency as demonstrated by its complete dissolution in water after 7 days. Similarly, transient gel‐like ionic conductors formed by CNC‐reinforced gelatin eutectogels (gels bearing deep eutectic solvents) have shown a dual‐response (resistive and optical) capability upon stretching.^[^ ^242^ ^]^ The ionic conductivity increased from 2.0 to 3.0 mS·cm^−1^ (0.5 wt% sulfated or carboxylated CNCs) upon stretching, while the spontaneous assembly of CNCs resulted in birefringent properties. Importantly, the dynamic, non‐covalent nature of the crosslinked network in the eutectogels enabled self‐healing upon heating at 50 °C, representing a simple approach toward sustainable recycling.

Another strategy involves fabricating chemically crosslinked hydrogels with dormant chemical moieties designed to react upon the application of an external stimulus. An example of this approach was reported by Saho et al., who exploited a Fenton‐like degradation process upon the incorporation of FeCl_3_ into a chemically crosslinked hydrogel comprised of TEMPO‐oxidized CNFs, polydopamine (PDA), and polyacrylamide (PAM).^[^ ^243^ ^]^ When immersed in water, the oxygen molecules penetrate the network to oxidize the catechol groups and release Fe^3+^. This is then reduced to Fe^2+^, further activating the residual S_2_O_4_^2−^ anions from the ammonium persulfate crosslinker. As a result, the epidermal sensor fully dissolved in 7 days, and completely degraded after 30 days. The coordination between CNF/PDA and Fe^3+^ ions together with extensive hydrogen bonding provided tensile strengths of 130 kPa, compressive strengths of 10 MPa, and remarkable elongation at break exceeding 2800%.

Aeby et al. fabricated transient relative humidity and temperature sensors (1 cm^2^) by screen printing electrically‐conducting inks onto 200 µm thick shellac films, a renewable and biodegradable resin used in hydrophobic coatings for wood.^[^ ^244^ ^]^ The inks were composed of graphite flakes, carbon black, and shellac in ethanol/pentanol, and showed the shear‐thinning behavior required for screen‐printing. As shown in Figure 15d, a humidity sensor was obtained using a capacitive transducer with interdigitated carbon electrodes. After coating with egg albumin, a relative capacitance change of 1.5 at 65% humidity and 35 °C was achieved. Furthermore, the sensor's dynamic response and recovery were enhanced by 20 times compared to paper‐based sensors. A temperature resistive detector offering a temperature coefficient of resistance of 5300 ppm·K^−1^ was also obtained upon processing the ink into a 100 mm long and 0.5 mm wide serpentine design. Importantly, both sensors could disintegrate by 84.5 wt% after 77 days under simulated aerobic composting conditions at 58 °C (ISO 20200, Figure 15d). A solution to circumvent the use of PDMS in mechanically flexible pressure sensors was proposed by Liu et al., who poured a solution of potato starch, glycerol, PVA, and water onto 60 µm thick polyimide film that was laser‐carbonized in a specific pattern. Peeling off the starch layer from the polyimide layer afforded the sensing film, with the patterned carbonized section transferred to the starch layer. Using this method, an impressive gauge factor of 134 and a response time of 130 ms was obtained.^[^ ^245^ ^]^ As shown in Figure 15e, the sensor demonstrated a complete disintegration into benign components after 10 min under running water.

More complex transient sensing devices bearing cellulose derivatives have also been developed. For example, Liu et al. demonstrated an environmentally degradable microfluidic device for sweat monitoring (rate, total volume, biomarkers) composed of a filter paper, a heat sealable cellophane layer (NatureFlex NVS film), and a biodegradable thermoplastic.^[^ ^246^ ^]^ Overall, sensing devices represent a prominent example of transient technology where designing for degradation facilitates low‐input operation, material reuse, and applications in situations where device retrieval is impossible or not economically feasible.

### 4.2 Transient Photonics

**Figure 16:**
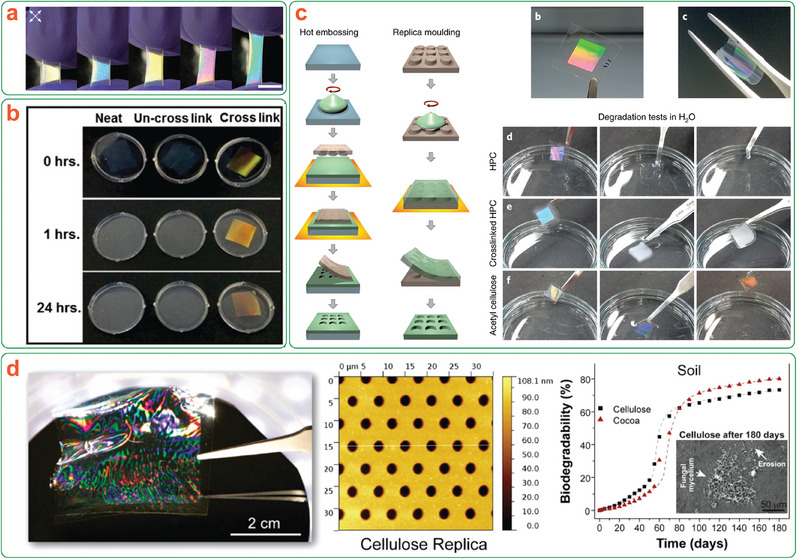
a) Chiral nematic CNC‐elastomer materials showing optical properties that vary upon stretching (photograph under crossed polarizers, arrows in image indicate polarization axes). Scale bar is 1 cm. Reproduced with permission.^[^ ^265^ ^]^ Copyright 2019, Nature‐Springer. b) Water stability of neat, non‐crosslinked, and glutaraldehyde crosslinked films over time. Reproduced with permission.^[^ ^267^ ^]^ Copyright 2020, American Chemical Society. c) Transient photonic HPC films based on 2D photonic crystal structures. Glass substrate, blue; HPC, green; PDMS mold, brown. The transiency of the photonic materials could be modified upon crosslinking. Reproduced with permission.^[^ ^222^ ^]^ Copyright 2018, Nature‐Springer. d) Photonic pure cellulose film: photograph of the 4 × 4 cm micropatterned film; AFM images showing the nanostructure of the film and soil biodegradation tests. Reproduced with modification and permission.^[^ ^268^ ^]^ Copyright 2020, American Chemical Society.

Transient photonics is an emerging area of research where materials with vivid colors change appearance over time or through the application of stimuli. While some examples of inorganic transient photonics have been shown using materials such as three‐layer Mg/MgO/Mg,^[^ ^247^ ^]^ these materials do not represent an adequate solution due to resource‐intensive extraction processes and the potentially harmful byproducts generated upon degradation. In this context, cellulose can be exploited to fabricate photonic materials that show an optical response and degrade without generating harmful byproducts. Notable contributions to the field of photonics have been made by exploiting the structural color that originates from the self‐assembly characteristics of CNCs. Early works date back to the early 2010s,^[^ ^155^, ^248^, ^249^ ^]^ with more recent notable contributions from the groups of MacLachlan,^[^ ^250^, ^251^, ^252^ ^]^ Vignolini,^[^ ^253^, ^254^ ^]^ and others.^[^ ^255^, ^256^, ^257^, ^258^, ^259^, ^260^, ^261^, ^262^, ^263^ ^]^ These chiral nematic CNC films present iridescent colors, wherein the reflected color can be modified by careful control of the chiral nematic pitch.^[^ ^264^ ^]^ As such, liquid absorption or the application of external mechanical forces can induce structural modifications, shifting the reflected light wavelength (**Figure** **16**a).^[^ ^265^ ^]^ Upon contact with water, CNC chiral nematic structures can re‐disperse. As shown in Figure 16b, these materials show physically transient properties that can be tuned through the use of a variety of crosslinkers such as acrylamides, methacrylates, or glutaraldehyde.^[^ ^266^, ^267^ ^]^

This phenomenon is not restricted to nanocellulose; other cellulosic materials with submicrometric periodic lattices can also display photonic properties. For example, HPC was molded by soft nanoimprinting lithography to obtain 2D photonic crystals.^[^ ^222^ ^]^ As shown in Figure 16c, a nanostructured PDMS mold enabled self‐standing HPC films with vivid colors that could be washed away in 30 s upon immersion in water, although the authors also demonstrated that crosslinking could enhance water resistance. Additionally, these plasmonic crystals showed an enhancement of Raman signal, showing intriguing potential as disposable SERS substrates. In another study, a replica molding approach was adopted to fabricate a high‐quality pure cellulose photonic material able to withstand humid environments while not compromising its inherent biodegradability.^[^ ^268^ ^]^ Cellulose was extracted from the cell walls of cocoa pod husks using a mixture of trifluoroacetic acid and trifluoroacetic anhydride, and was subsequently patterned with hexagonal and cubic lattices (Figure 16d). Biodegradability studies in soil according to ISO 17556 indicated >60% enzymatic biodegradation in 100 days. Importantly, the photonic films maintained their structure after immersion in distilled water for 3 months, showing a balance between biodegradability and reliable operation in aqueous environments. Overall, these cellulose‐based photonic materials do not leave behind harmful degradation products after their function has been accomplished, offering a new paradigm to replace current long‐lasting and potentially toxic dyes and colorants in cosmetics, artworks, textiles, or sensors.

### 4.3 Transient Energy Storage

**Figure 17:**
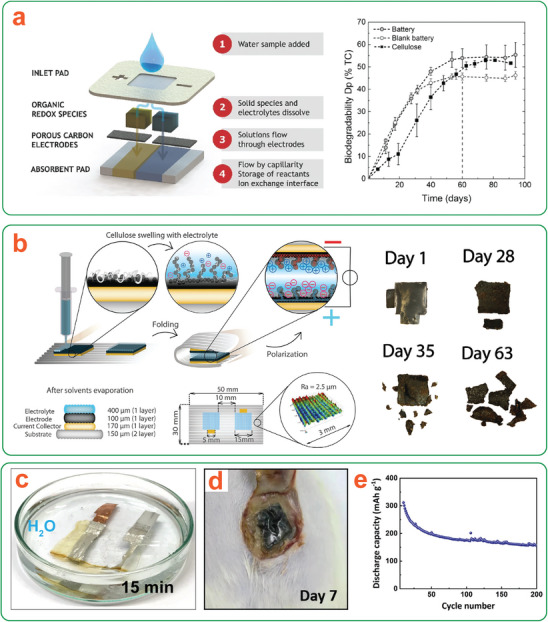
Transient energy storage devices enabled by cellulosic materials: a) Primary biodegradable battery composition and its biodegradation according to initial total carbon content (%TC). Reproduced with permission.^[^ ^270^ ^]^ Copyright 2017, Wiley‐VCH. b) Scheme showing the structure and functioning mechanism of the 3D printed supercapacitor containing both CNCs and CNFs. Reproduced with permission.^[^ ^272^ ^]^ Copyright 2023, Wiley‐VCH. c) A transient LIB (Li‐V_2_O_5_ system) having a PVA/CNC‐Li membrane soaked with the Bmim‐TFSI IL disintegrates in water in 15 min. Reproduced with permission.^[^ ^274^ ^]^ Copyright 2021, Wiley‐VCH. d) In vivo degradation and biocompatibility evaluation of a surgically implanted transient ZIB after 7 days after implanted subcutaneously in rats. Reproduced with permission.^[^ ^275^ ^]^ Copyright 2022, Wiley‐VCH. e) Cycling performance at 50 mA·g^−1^ of the compostable battery composed of CMC/agarose hydrogel, agarose encasing, PDA/carbon cathode, and zinc metal anode. Reproduced with permission.^[^ ^276^ ^]^ Copyright 2023, Wiley‐VCH.

Electrochemical energy storage (EES) devices play a key role in current decarbonization and electrification efforts.^[^ ^269^ ^]^ In this context, the development and implementation of electrochemically efficient, safe, and environmentally sustainable batteries is a top priority toward the 21^st^ century sustainable society. However, most current EES devices (e.g. Li‐ion batteries) use scarce, potentially toxic, and non‐degradable materials such as cobalt, nickel, or manganese. Thus, several research groups have directed their efforts to use cellulosic materials to develop electrochemically competitive batteries and supercapacitors. The first attempts to fabricate transient batteries using celluloses were focused on primary (single‐use) batteries. In 2017, Esquivel et al. used cellulose, carbon paper, beeswax, and quinone‐based chemistry (p‐benzoquinone/oxalic acid and hydroquinonesulfonic acid potassium salt/KOH half‐cells) to develop a battery that could be biotically degraded (54 ± 4% total carbon content) after 60 days under anaerobic conditions (**Figure** **17**a).^[^ ^270^ ^]^ A series of patterned cellulosic layers (from Ahlstrom) provided a co‐laminar flow by capillarity, mixing the two reactant streams and activating the battery upon the addition of a small sample of liquid (i.e., water, urine, saliva). Although the battery could power small electronic devices, it could only operate for 100 min. Using eco‐design concepts, the same group developed a flow battery with extended operating lifespan able to mimic the fluid transport in plants for precision agriculture applications.^[^ ^271^ ^]^ A 110 × 12 × 1.18 mm^3^ U‐shaped paper (Whatman filter paper) system pumps the reactants toward porous carbon electrodes thanks to a capillarity‐transpiration combination analogous to transpiration pull in plants. The complete battery pads (with reactants) showed a biodegradation of 90 ± 6% of total carbon content after 18 days under aerobic conditions (EN 13432). Importantly, germination indexes above 100% indicated the battery biodegradation by‐products were actively nurturing the surrounding environment as they were produced.

Interestingly, additive manufacturing technologies such as 3D printing can exploit the shear‐thinning behavior of nanocelluloses to fabricate energy storage devices with pre‐designed patterns. Aeby et al. used direct‐ink‐writing to extrude cellulosic gel inks in a layer‐by‐layer fashion to cover a CNF/CNC/glycerol substrate with a shellac/carbon current collector, a carbon/CNF/CNC electrode, and a CNC/glycerol/NaCl electrolyte (structure and ion conducting process in Figure 17b). The resulting electrochemical double layer capacitor offered a capacitance of 25.6 F·g^−1^ with an open circuit potential of 1.2 V, being able to degrade by 50 wt% within 9 weeks.^[^ ^272^ ^]^ Additional benefits from gel electrolytes, where the liquid components remain immobilized within a solid (often polysaccharide‐based) matrix include enhanced safety, the potential to extend working temperature ranges, and improved interfacial properties compared to liquid and all‐solid electrolytes.^[^ ^273^ ^]^

More recently, nanocellulosic materials have also been used to fabricate proof‐of‐concept secondary (rechargeable) transient batteries. In 2021, Mittal et al. exchanged the proton of the ‐OSO_3_H groups on the CNC surface with Li^+^ to fabricate a membrane material for secondary Li^+^‐ion battery applications.^[^ ^274^ ^]^ The inclusion of porous PVA/CNC membranes fabricated by a non‐solvent‐induced phase separation approach showed an improved ion transference and electrode‐electrolyte interface. As shown in Figure 17c, a proof‐of‐concept fully transient Li/V_2_O_5_ battery that could disintegrate in water in only 15 min at room temperature was constructed using an IL‐soaked (Bmim‐TFSI) separator, delivering 55 mAh·g^−1^ after 200 cycles at 100 mA·g^−1^ current rate. Secondary transient battery research has evolved from this first example toward more environmentally benign chemistries, i.e. zinc‐ion batteries (ZIBs), with aqueous electrolytes and non‐toxic cathode materials. For example, Zhou et al. designed a transient ZIB based on a cellulose aerogel‐gelatin solid electrolyte, a silk fibroin casing, a MnO_2_/rGO cathode, and a zinc‐particle anode.^[^ ^275^ ^]^ The cellulose aerogel offered a mechanically resilient porous framework, and the grafted gelatin facilitated ZnSO_4_ salt solubility and enhanced ionic conductivity. The assembled battery offered an increased specific capacity over the previous transient LIB design (212 mAh·g^−1^ at 61.6 mA·g^−1^) and could completely biodegrade after 30 days in buffered proteinase K solution at 37 °C. Besides, a good level of biocompatibility was achieved using both in vitro and in vivo tests (Figure 17d), demonstrating potential clinical applications. In 2023, Mittal et al. reported a ZIB able to disintegrate under composting conditions.^[^ ^276^ ^]^ The chemical crosslinking of CMC and agarose using glutaraldehyde afforded a mechanically adaptable porous structure with an outstanding ionic conductivity of 87.6 mS·cm^−1^ (2 M ZnSO_4_), rendering a long‐term cyclability of over 10 000 cycles at a current density of 1 A·g^−1^. When assembled with a biodegradable agarose encasing and a PDA‐based cathode, a specific capacity of 157 mAh·g^−1^ after 200 cycles at a current density of 50 mA·g^−1^ was achieved (Figure 17e). Importantly, the battery showed a weight loss of 49.9 ± 2.9 wt% after 63 days under standardized composting conditions (ISO 20200).

Other bio‐based materials have been also implemented for transient ZIBs. For instance, chitin nanofibrils isolated from fungi formed a hydrogel upon glutaraldehyde crosslinking that offered an ionic conductivity of 54.1 mS·cm^−1^, electrolyte uptake of 4330% (2 M ZnSO_4_) and Zn^2+^ transference number of 0.47.^[^ ^11^ ^]^ This gel, which can withstand dendrite puncture at areal current densities as high as 9.5 mA·cm^−2^, was implemented with polyester/carbon black current collectors and a Zn/𝛼‐MnO_2_ cell for a battery that decomposes in water at 70 °C.

By avoiding the use of conventional CRMs, transient electrochemical energy storage devices offer undeniable economic and environmental benefits. Additionally, the ability to repurpose “waste” biomass or reuse the degraded components of other (nano)cellulosic transient devices to create carbonaceous electrode materials keeps raw materials cycling within the loop, increasing cost effectiveness, reducing the need for raw material extraction/refining and easing EoL scenarios.

### 4.4 Transient Electronics

As mentioned in the Introduction, the increased use of electronic devices is resulting in an overwhelming amount of e‐waste. This environmentally and biologically toxic waste is hardly recycled (only 17.4% of global e‐waste is treated),^[^ ^277^ ^]^ so the destination of the lost e‐waste is of increasing concern. In this context, cellulosic materials have been explored to develop degradable electronics that do not leave behind harmful by‐products after use.

**Figure 18:**
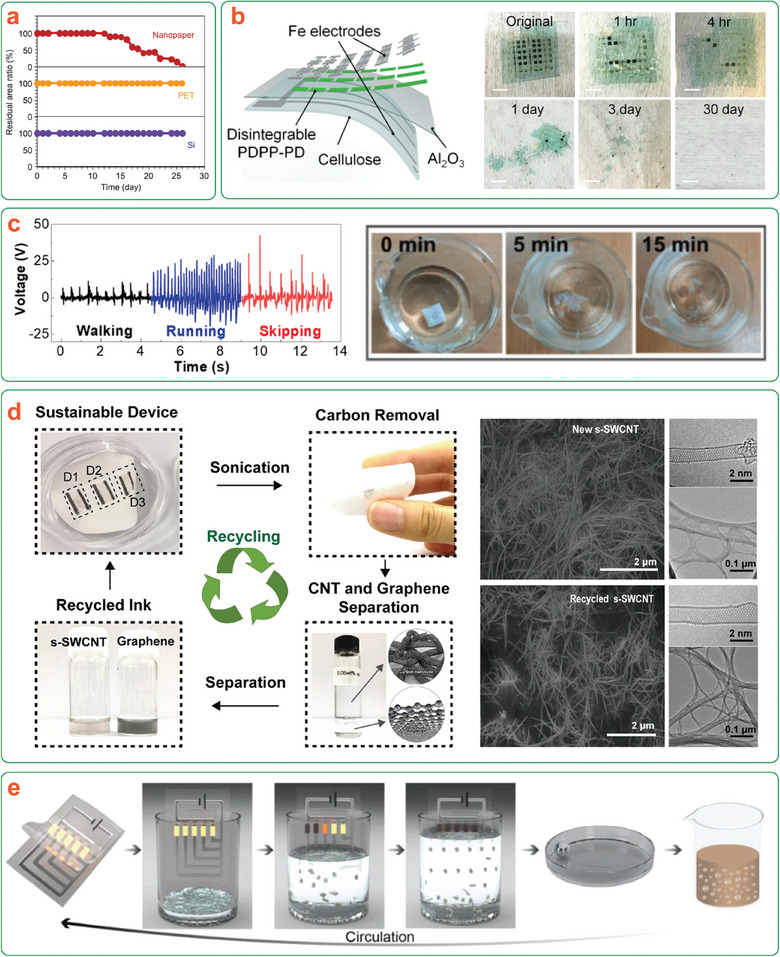
a) Biodegradability in natural soil (22 ± 2 °C; 92 ± 2% humidity) of the memory devices based on nanopaper (CNFs), PET, and Si substrates. Reproduced with permission.^[^ ^279^ ^]^ Copyright 2016, Nature‐Springer. b) Transient thin‐film transistor structure and its disintegration in pH 4.6 1 mg·mL^−1^ cellulose solution. Scale bars are 5 mm. Reproduced with permission.^[^ ^280^ ^]^ Copyright 2017, National Academy of Sciences. c) Voltage output of in response biomechanical motion and degradation of PDA‐coated BC in 80% w/v citric acid aqueous solution at 90 °C. Reproduced with permission.^[^ ^281^ ^]^ Copyright 2022, Elsevier. d) Schematic showing the carbon nanomaterial removal procedure and its morphology. Reproduced with permission.^[^ ^282^ ^]^ Copyright 2022, American Chemical Society. e) Transiency of electrically conducting circuits composed of lignin/room‐temperature liquid metals upon immersion in water at room temperature. Room‐temperature liquid metals are then recovered. Reproduced with permission.^[^ ^283^ ^]^ Copyright 2023, Wiley‐VCH.

As one of the first examples in transient electronics using cellulosic materials, Jung et al. demonstrated in 2015 that the design of several electronic systems essential to electronic device construction could all be realized using TEMPO‐oxidized CNF films as substrates.^[^ ^278^ ^]^ GaAs‐based heterojunction bipolar transistors, Schottky diodes, passive inductors and capacitors, and Si‐based CMOS digital devices were all fabricated onto freestanding CNF films (either 80 or 200 µm thick) coated with bisphenol A‐based epoxy (to facilitate its handling during device fabrication). The devices showed comparable performance to their conventional counterparts and could be partially degraded by the brown rot fungus *Postia placenta* after 60 days. Subsequent attempts for organic transient electronics have used cellulosic materials as substrates for active layers. In 2016, Celano et al. developed a transparent non‐volatile memory device using a ≈30 µm thick CNF substrate, a 100 nm thick indium tin oxide coating layer, an additional nanocellulose resistive switching layer, and 100 nm‐thick Ag top electrodes, reaching a remarkable 99.3 vol.% of CNFs in the device.^[^ ^279^ ^]^ The 2–3 orders of magnitude lower root‐mean‐square surface roughness of the nanopaper (7.4 nm) over regular paper or petroleum‐based plastic surfaces enabled a very thin coating of active materials, guaranteeing the continuity of electrical conduction. After removing the Ag from the device by sonication in water (its antibacterial effect suppresses fungal biodegradation), a complete degradation in natural soil was observed after 26 days, in striking contrast with the petroleum‐based PET and inorganic Si substrates (**Figure** **18**a). Further advances were reported in 2017 when transient thin‐film transistors (< 1 µm) were fabricated by coating a conjugated polymer, an Al_2_O_3_ dielectric layer, and iron gate and source‐drain electrodes onto an 800 nm thick hydrolyzed cellulose substrate.^[^ ^280^ ^]^ As shown in Figure 18b, the device began to degrade after 1 h in a pH 4.6 1 mg·mL^−1^ cellulose solution and completely degraded after 30 days.

A series of triboelectric nanogenerators (TENGs), where potentials are generated by the triboelectric effect through the use of materials with opposing tribo‐polarity, were developed using in situ growth of PDA, polypyrrole (PPy) or SiO_2_ on BC.^[^ ^281^ ^]^ PDA incorporation enabled electron release and enhanced the power density of pristine BC by seven times. When working as a single‐electrode positive tribolayer, the resulting device could generate 43 V in response to biomechanical motion (walking, running, skipping). Importantly, the materials completely disintegrated within 15 min when immersed in an 80% w/v aqueous citric acid solution at 90 °C, opening the path to environmentally sustainable and wearable biomechanical energy harvesters (Figure 18c). In another work, CNCs were incorporated into polyhydroxybutyrate (PHB) films by high‐pressure molding to obtain transient TENGs with an enhanced dielectric constant and a 5.7‐fold increase in the voltage output (≈8 V) over pristine polyester when in the presence of 5 wt% CNCs.^[^ ^284^ ^]^ CNCs enhanced material hydrophilicity, markedly accelerating hydrolytic chain scission events in PHB (pH 12 with NaOH; 25 °C) resulting in a 16 wt% loss after 176 days. Besides, Kim et al. recently developed transient organic light‐emitting diodes through a novel transiency mechanism based on a predetermined voltage input that triggered device combustion via Joule‐heating.^[^ ^285^ ^]^ Aside from common active materials for diode function (Ag, Al, MoO_3_, etc.), a 1 µm thick phase change material composed of microcrystalline wax was coated onto the nitrated paper substrate to trigger combustion reactions and attain complete transiency after 10 s when stimulated with a voltage input of 9 V.

Over the last few years, we have seen several approaches using cellulose to enable facile recapture of the environmentally and biologically damaging components of electronic devices. An example was reported by Williams et al., who fabricated thin‐film transistors using a CNC shear‐thinning conducting ink (dielectric material), carbon nanotubes as a semiconductor, and graphene as a conductor.^[^ ^286^ ^]^ Upon deposition by aerosol jet printing onto paper substrates, the thin‐film transistor could operate for >6 months after adding 0.15 mM NaCl with on/off‐current ratios of 10^4^, while tip horn sonication enabled >95% retrieval rates for CNTs and graphene (Figure 18d). A similar concept for the effective recycling of electronic devices (a thin‐film transistor, on/off ratio 176) was also shown by Thi et al.,^[^ ^282^ ^]^ who coated a cellulose filter paper using a CNC/NaCl slurry to obtain a highly dielectric material. The 0.2 µm pores of the filter paper facilitated the deposition of SWCNTs (semiconducting material) and graphene (back‐gate) by simple vacuum filtration. An aqueous two‐phase extraction with dextran, PEG, and 0.05 wt% sodium cholate surfactant allowed the recovery of >70 wt% of SWCNTs and >90 wt% of graphene after 5 recycling steps. The device degraded in the natural environment in ≈7 days, which is especially useful in scenarios where material recovery methods are not feasible. Other natural polymers have also proven efficient for electronics recycling. In one study, the polyphenol functional groups and self‐assembly properties of lignin were exploited to encapsulate room‐temperature liquid metals (eutectic gallium–indium) by probe sonication and develop highly conductive printed inks (6.8 × 10^4^–1.45 × 10^6^ S·m^−1^) that remained stable when bent or twisted.^[^ ^283^ ^]^ As depicted in Figure 18e, the circuits, when printed onto PVA, underwent deformation, cracking, and disintegration upon immersion in water at room temperature for <100 s. A subsequent 0.5 M NaOH treatment dissolved the lignin and released the liquid metals for their recovery (96.9%) and reuse.

Nanocelluloses, in combination with other naturally‐sourced polymers, show synergetic effects for transient electronics. In this context, ionic diodes based on agarose hydrogels doped with n‐type CNCs (glycidyl trimethylammonium chloride modification) and p‐type CNCs (sulfonation) have been constructed.^[^ ^287^ ^]^ The high charge density of CNCs played a pivotal role, where the anisotropic distribution of counterions at the interface between the two gels yielded a diode operating at current densities of 6 mA·cm^−2^, with a rectification ratio of 70. Although the transiency was not assessed, potential biodegradability was claimed. In another report, epoxidized groups in lignin sulfonate were crosslinked with surface hydroxyl groups of CNF to obtain 150 µm thick films with a remarkable ultimate tensile strength of 146 MPa and elastic modulus of 16.2 GPa.^[^ ^288^ ^]^ The low surface roughness of 4.7 nm (lower than the ≈10 nm of petroleum‐derived plastics) enabled the deposition of a Mg coil onto the films to fabricate radio frequency identification antennas that could undergo complete degradation in one rainy day.

The transiency and performance shown by celluloses have sparked the research of other renewable materials for electronic applications. Relevant examples include the fabrication of ionotronic skin for wearable electronics with a full degradation in 3 days in PBS (crosslinked carboxylated chitosan/sulfobetaine methacrylate),^[^ ^289^ ^]^ dual crosslinked chitin hydrogels fabricated by screen‐printing with complete biodegradation in soil after ≈120 days,^[^ ^290^ ^]^ or transparent speakers based on chitin/Ag nanowires that disintegrated in a chitinase solution after 8 days (23 °C).^[^ ^291^ ^]^ Transient electronics are a rapidly growing field of research, as they can facilitate further use of otherwise environmentally damaging devices in situations where retrieval is not possible, and can be designed to be upcycled and reused, limiting the production of e‐waste worldwide and lowering its environmental burden.

### 4.5 Transient Films and Coatings for Food Preservation

**Figure 19:**
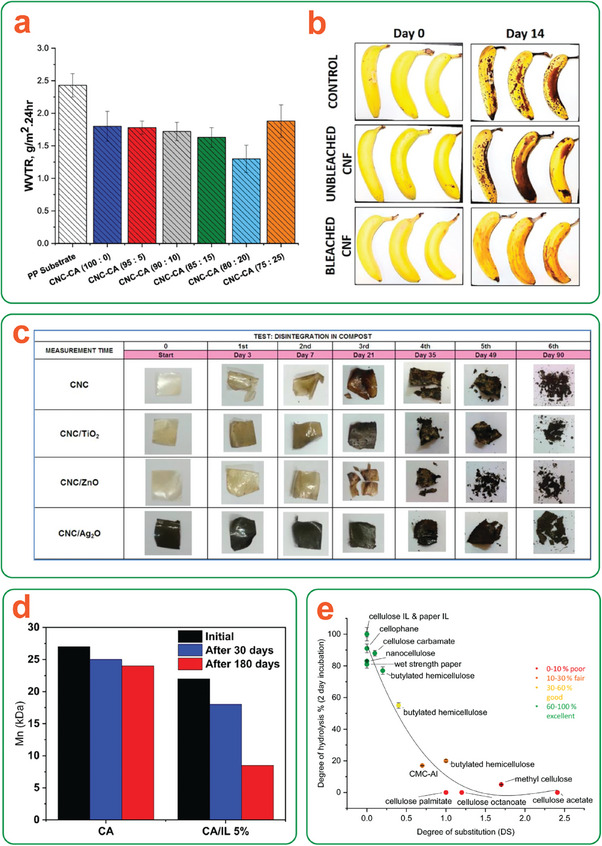
a) Water vapor transmission rate (WVTR) of CNC and CNC/ citric acid‐coated PP films. Reproduced with permission.^[^ ^293^ ^]^ Copyright 2020, American Chemical Society. b) Effect of bleached and non‐bleached CNF coatings on the visual appearance of bananas when stored at 21 °C and 60% relative humidity. Reproduced with permission.^[^ ^294^ ^]^ Copyright 2022, American Chemical Society. c) Visual observation of 15 × 15 mm CNC‐based freestanding films under aerobic composting. Reproduced with permission.^[^ ^295^ ^]^ Copyright 2018, American Chemical Society. d) M_n_ of CA and CA/immobilized lipase upon immersion in water at 37 °C. Reproduced with permission.^[^ ^100^ ^]^ Copyright 2023, American Chemical Society. e) Biodegradation of cellulose‐based films by enzymatic hydrolysis in a 2‐day incubation depending on the DS. Reproduced with permission.^[^ ^102^ ^]^ Copyright 2020, Springer‐Nature.

Given the environmental pollution caused by conventional petroleum‐derived packaging materials such as polyethylene (PE) or PS, (nano)celluloses have been widely used to develop (nano)composite materials and improve the functional properties of films aimed at food packaging applications.^[^ ^292^ ^]^ For example, the tortuosity provided by nanocelluloses can hinder gas diffusion through the packaging film, which in combination with the high polarity of the cellulose results in decreased gas permeability for oxygen and carbon dioxide, while offering a higher moisture barrier property (reduced water vapor transmission rates) over the non‐reinforced material (**Figure** **19**a).^[^ ^293^ ^]^ An interesting use of nanocelluloses for extending the shelf life of fruits and vegetables was reported by Amoroso et al.,^[^ ^294^ ^]^ who spray‐dried bleached CNF isolated from carrots (0.25–1.0 wt% in water to allow shear‐thinning and rapid viscosity recovery behavior) to obtain transparent coatings onto bananas that significantly lowered the enzymatic browning of the peel after 14 days (Figure 19b). Nanocelluloses can also serve as controlled‐release agents and stabilizers to provide antioxidant and antibacterial properties, and thus prolong the shelf life of packaged foods.^[^ ^292^ ^]^ Although the majority of these studies use the terms “biodegradable” or “edible”, such assumptions are not often proven and rely on the fact that the individual composite constituents are biobased, so transiency may be expected. In this context, the degradation of freestanding CNC films produced by evaporation under simulated aerobic composting conditions at 58 °C (ISO 20200) was investigated by Lizundia et al. They observed fragmentation after 21 days,^[^ ^295^ ^]^ and after 35 days the 30 µm thick CNC films had lost 60% of their mass. The rate of composting degradation of CNCs was notably reduced upon the incorporation of Ag_2_O nanoparticles because of a reduced attack by microorganisms (5 wt% loss after 90 days), suggesting a microbial degradation mechanism.

Dynamic covalent modification of cellulose is a clever approach to tune thermal and mechanical performance while ensuring biodegradability. In this context, Zhou et al. partially disassociated the hydrogen bonds in MCC and subsequently assembled the cellulose chains to form a dynamic covalent network that enhanced cellulose thermoprocessability, a nearly essential characteristic for the practical industrialization of packaging materials. Furthermore, the material offered a tensile strength of 67 MPa, and balanced its good moisture and solvent resistance with biodegradability (100% mass loss in 50 days of soil burial).^[^ ^296^ ^]^ Cellulose esters and ethers also display interesting properties for transient packaging. Zhang et al. demonstrated the potential of CMC films incorporating silver‐based metal–organic frameworks for greener packaging applications over commercial PE films. The presence of silver in the composite films endowed them with antimicrobial properties against bacteria and mold, improving fruit preservation. Additionally, the composite films were completely degraded after 45 days in soil at room temperature and 65–75% relative humidity.^[^ ^297^ ^]^ For esters, an example with potential for transient food preservation applications is cellulose diacetate, which showed a 70 wt% loss after 11 weeks by marine microbes.^[^ ^298^ ^]^ This degradation process can be accelerated by the inclusion of photocatalysts (TiO_2_) that trigger chain scission reactions under sunlight illumination, doubling the mass loss rate and yielding lower‐molecular‐weight products and CO_2_.^[^ ^299^ ^]^ Physically entrapped enzymes within cellulosic derivatives can also speed up biodegradation processes; i.e. degradation reactions are triggered when the materials meet the catalytic medium. This strategy was proven effective using CA and a lipase isolated from *Candida rugose*, where the M_n_ was reduced by half after 180 days immersed in water (Figure 19d). Finally, Figure 19e offers sound guidance for future transient cellulosic food packaging applications: DS is strongly negatively correlated to the susceptibility of cellulose to enzymatic hydrolysis.^[^ ^102^ ^]^

(Nano)cellulosic transient materials show a great application field in food packaging, where the goal is to enhance food shelf‐life while reducing non‐degradable plastic waste. The design of transient food packaging is relatively simple compared to other application fields, and the materials should be available at affordable cost considering current advances in (nano)cellulosic manufacturing processes.

### 4.6 Transient Materials for Biomedical Applications

Transient materials present undeniable advantages in biomedical applications as they can degrade into non‐toxic byproducts with no need for additional surgery for device retrieval. This strategy lowers potential risks, chronic inflammation, and economic costs associated with permanent devices.^[^ ^300^ ^]^ When in the human body, cellulose and its derivatives can undergo degradation processes through enzymatic breakdown, hydrolysis, oxidation, or photooxidation, facilitating sustainable healthcare system development. Besides, these materials have highly tunable morphology, mechanical properties, hydrophilicity, biocompatibility, and degradation time scales.

**Figure 20:**
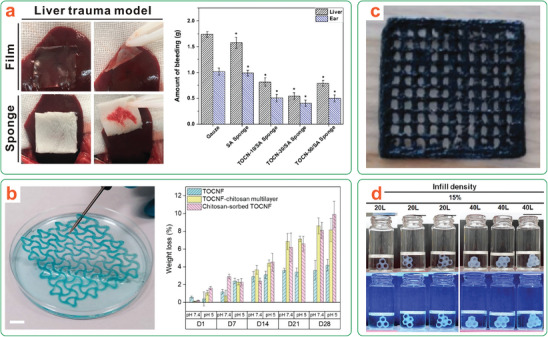
a) Hemostatic effect of neat alginate (SA) and its TEMPO‐oxidized cellulose nanocrystal (TOCN) composite materials. Reproduced with permission.^[^ ^302^ ^]^ Copyright 2017, American Chemical Society. b) Optical photograph of a 3D printed TEMPO‐oxidized CNF/chitosan mesh structure (scale bar is 1 cm) together with its weight loss at pH 7.4 and 5. Reproduced with permission.^[^ ^304^ ^]^ Copyright 2022, American Chemical Society. c) 25 × 25 mm printed scaffold in wet state with 5% PPy having a 1:2 weight ratio of pre‐PGS‐PPy:TOCNF. Reproduced with permission.^[^ ^305^ ^]^ Copyright 2020, Wiley‐VCH. d) Digital photographs of the 3D printed photoluminescent hydrogels under visible and UV light irradiation. Reproduced with permission.^[^ ^306^ ^]^ Copyright 2023, Elsevier.

Oxidized cellulose is a suitable material for surgical applications as it is bioresorbable, non‐toxic, and hemostatic. In fact, oxidized regenerated cellulose can be found commercially in the form of knitted fabric for wound healing that prevents postsurgical adhesions because of the formation of a physical barrier between adjacent tissues.^[^ ^301^ ^]^ In this context, Cheng et al. showed that TEMPO‐oxidized CNCs could be used as a reinforcing agent to enhance the hemostatic efficiency of Ca^2+^ crosslinked alginate materials (films and sponges).^[^ ^302^ ^]^ In particular, as shown in **Figure** **20**a, blood loss in the rabbit ear artery and liver trauma models was reduced in the presence of TEMPO‐oxidized CNCs due to the increased absorbance of wound exudates. Furthermore, the resulting composites could be completely biodegraded without an inflammatory response after three weeks of subcutaneous implantation in rabbits. Similarly, carboxylic functional groups can be introduced to regenerated cellulose by TEMPO‐oxidation to achieve a material with suitable mechanical properties and accelerated biodegradability for suture applications. In particular, the in vitro degradation in physiological saline at 37 °C increases from 8 to 36% after 28 days due to increased molecular chain spacing and reduction in molecular inter‐atomic forces.^[^ ^303^ ^]^ This material adheres to the tensile strength requirements for sutures, revealing potential prospects for clinical applications.

Nanocelluloses serve to enhance the functional properties of other biobased polymers whose mechanical properties are not fully fit for biomedical applications. In this vein, Ajdary et al. incorporated TEMPO‐oxidized CNFs into chitosan to fabricate layer‐by‐layer meshes. Negatively‐charged nanocelluloses interact with positively charged chitosan to render shear‐thinning behavior that allowed a good shape fidelity of 3D printed mesh structures, increased the mechanical modulus by two times and accelerated degradation at pH 7.4 (Figure 20b), making these materials promising candidates for implant administration.^[^ ^304^ ^]^

Nanocellulose‐based scaffolds (78 ± 2% porosity, Figure 20c) aimed for cardiac patches were constructed by direct ink writing of an aqueous ink containing TEMPO‐oxidized CNFs, poly(glycerol sebacate) (PGS), and PPy followed by freeze‐drying.^[^ ^305^ ^]^ PPy provided electrical conductivity (34.0 ± 2.7 mS·cm^−1^) to mimic the dynamic functions of electroresponsive cardiac tissues, while TEMPO‐oxidized CNFs mechanically reinforced the structure to reach a Young's modulus of 0.60 ± 0.16 MPa (mimicking the elastic modulus of human myocardium). Furthermore, the carboxylic groups on TEMPO‐oxidized CNFs enhanced the swelling of the patches to >200%. These cardiac patches proved useful for long‐term therapy as they stimulated cell growth and prevented burst release. In another work, TEMPO‐oxidized CNCs and carbon dots (CDs) were processed into pre‐designed shapes by digital light processing bioprinting for dynamic cell tracking. The gels were highly photoluminescent (Figure 20d), and remained effective up to 30 days after printing.^[^ ^306^ ^]^ Nanocellulose provided appropriate shear‐thinning characteristics to ensure a good shape fidelity and aided in matching the mechanical properties of the hydrogels with those of soft living tissues.

Furthermore, the biodegradability, non‐toxicity, hydrophilicity, and structure‐tuning ability of cellulose have been exploited to mimic the extracellular matrix and provide a suitable microenvironment for diverse tissue engineering applications. For example, biodegradable auxetic structures formed by BC with prescribed opening size and infill density have been developed for implantable meshes.^[^ ^307^ ^]^ Using mold‐guided growth of BC, the mechanical strength of the cellulose was tailored from 48 to 456 MPa. The auxetic character of BC helps to mimic the anatomical changes during movements and dynamic deformations in living tissue. A minimal 4 wt% loss after 28 days at pH 5 indicated the suitability of these materials for long‐term tissue support. In another work, oxidized BC‐reinforced chitosan scaffolds were used to efficiently regulate the nucleation and growth of hydroxyapatite, particularly relevant for bone regeneration.^[^ ^308^ ^]^ The presence of oxidized BC enhances scaffold flexibility, increases water retention, and offers a stable degradation rate over chitosan‐only scaffolds. In 2021, Wei et al. used oxidized BC conduits as a scaffold to host chitosan nanoparticles with encapsulated nerve growth factors to repair sciatic nerve defects in rats.^[^ ^309^ ^]^ The geometry afforded by mercerized BC tubular grafts was suitable for blood vessel replacement,^[^ ^310^ ^]^ while selectively oxidized BC with soybean protein isolate could repair urethral damage in rabbits (degradation speeds up after modification from ≈3 to ≈50% after 48 days in PBS at 37 °C).^[^ ^311^ ^]^ The use of transient (nano)cellulosic materials in biomedicine could improve the efficiency of medical processes by limiting repeated interventions after surgical procedures. As such, we expect that research in this field will continue to increase into the future, providing benefits for patients and medical professionals alike.

### 4.7 Transient Materials for Wastewater Treatment

With the increasing contamination of worldwide drinkable water sources by dyes from industrial effluents, microplastics, per‐ and polyfluoroalkyl substances, heavy metals, oils, or other solvents, and emerging contaminants from pharmaceuticals, the need for efficient wastewater treatment technologies has never been as pressing. In this context, the 2030 Agenda for Sustainable Development has identified water preservation and treatment as a top priority (SDG 6). Owing to their hydrophilicity, ease of chemical functionalization, and ability to host active materials, cellulose and its derivatives have a long history of use in wastewater treatment, and several recent reviews have been published focusing on this topic.^[^ ^312^, ^313^, ^314^, ^315^, ^316^ ^]^ The main strategies for wastewater treatment involving cellulosic materials include absorption (involving a number of possible mechanisms: electrostatic interactions, H‐bonding, ion‐exchange, hydrophobic interactions, π–π interactions),^[^ ^317^ ^]^ as a support material for (photo)catalysis,^[^ ^318^ ^]^ filtration,^[^ ^319^ ^]^ coagulation‐flocculation,^[^ ^320^ ^]^ or assisting in sedimentation of pollutants.^[^ ^321^ ^]^

**Figure 21:**
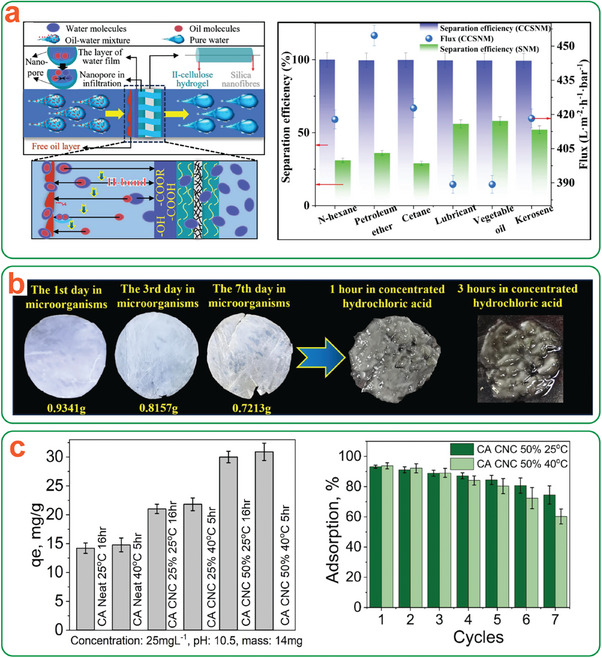
a) Schematic showing design and mechanism of action for the SiO_2_‐cellulose composite membrane used for oil/water separation, and the membrane performance as a filter (blue bars: composite membrane; green bars: SiO_2_ membrane only). Reproduced with permission.^[^ ^322^ ^]^ Copyright 2024, Elsevier. b) Degradation of the SiO_2_‐cellulose composite membrane first by microbes (degradation of cellulose) followed by acid treatment (degradation of SiO_2_). Reproduced with permission.^[^ ^322^ ^]^ Copyright 2024, Elsevier. c) Methylene blue absorption performance of electrospun CA/CNC nanofibers at different concentrations of CNCs, and cycling performance of nanofibers containing 50% CNCs. Reproduced with permission.^[^ ^323^ ^]^ Copyright 2024, Elsevier.

However, there is a dearth of literature describing (nano)cellulose‐based wastewater treatment materials designed specifically for transiency. The majority of works claim potential biodegradability to support the use of (nano)cellulose, but few provide detailed insights on this aspect of material design. Undoubtedly, however, there is great potential for the design of transient wastewater treatment materials using (nano)cellulose. A recent example highlighting this potential was provided by Pan et al.¸ who used regenerated cellulose to make a transient silica nanofiber/cellulose nanocomposite for applications in oil–water emulsion separation.^[^ ^322^ ^]^ A nanofibrous silica network was obtained by electrospinning, which provided a porous structure for the infiltration of dissolved cellulose (NaOH/urea/H_2_O at 7/12/81 wt%). Regeneration in anhydrous ethanol yielded 10 nm pores on the surface of the material. The hydrophilic functional groups of the modified cellulosic membrane (‐COOR, ‐COOH, ‐OH) were capable of bonding water molecules to form an H‐bonded water layer that rejected oil. When tested for the filtration of *n*‐hexane, petroleum ether, cetane, lubricant oil, vegetable oil, and kerosene, separation efficiencies of >99% were obtained, and the flux rate ranged from 395 to 460 L∙m^−2^∙h^−1^∙bar^−1^, significantly outperforming average oil rejection and flux rates of 93% and 63 L∙m^−2^∙h^−1^∙bar^−1^ for nanofiltration membranes (**Figure** **21**a). As seen in Figure 21b, the cellulosic phase completely degrades under microbial conditions (activated sludge) in 7 days, then the SiO_2_ membrane can be degraded with concentrated HCl.

Although not specifically touted as transient or degradable in many reports, the use of (nano)cellulosic materials for (hydro)gels and membranes that can be dissolved and regenerated after their use has great potential. As an example of this type of application, Khatri et al. fabricated electrospun composites based on CA and CNCs for the removal of methylene blue dye, a common industrial effluent.^[^ ^323^ ^]^ Using a 2:1 mixture of acetone:DMAc to spin CA‐based nanofibers containing 0, 25, or 50% CNCs, they found that composites containing 50% CNCs exhibited an almost twofold increase in methylene blue absorption compared to neat CA fibers (30 mg∙g^−1^ vs 14.1 mg∙g^−1^ at pH 10.5, 25 mg∙L^−1^ dye, 25 °C). The authors attributed this result to the increased surface area/porosity, higher hydrophilicity, and more negative surface charge of CNC‐containing composites. The material could be regenerated through the removal of methylene blue under mildly acidic conditions (0.05 M HCl, 25 °C, 1 h) followed by soaking in ethanol for 24 h, resulting in a ≈80% performance retention after five cycles (Figure 21c). While the transiency of the material was not tested, the possibility for regeneration over multiple cycles followed by eventual degradation fits very well within the transient devices design paradigm and offers an optimistic outlook for the development of future materials.

### 4.8 Other Uses for Transient Devices

**Figure 22:**
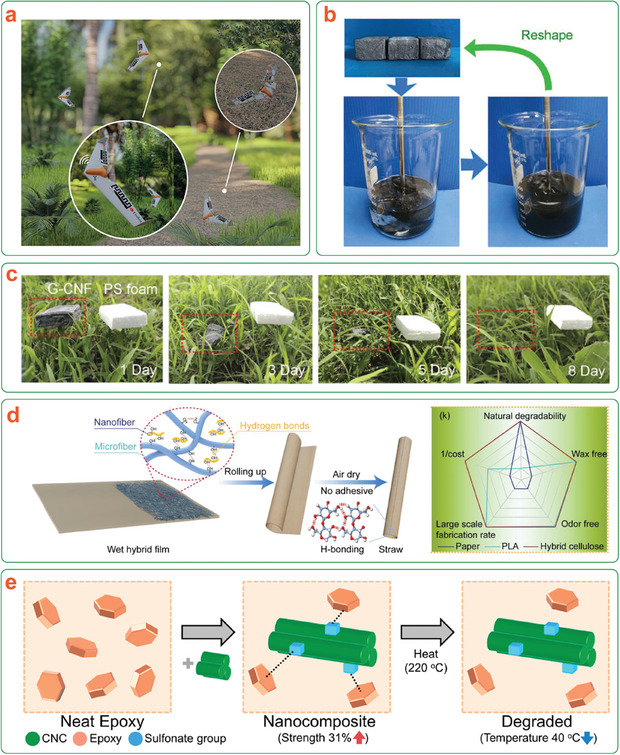
a) Transient drone based on gelatin crosslinked MFC cryogels. Reproduced with permission.^[^ ^324^ ^]^ Copyright 2023, Wiley‐VCH. b) Recyclability demonstration for the CNF/graphite aerogel. c) Degradation behavior of the CNF/graphite aerogel under ambient conditions (black sample). The white foam material is a commercial polystyrene foam. Reproduced with permission.^[^ ^326^ ^]^ Copyright 2021, Wiley‐VCH. d) Scheme depicting the rolling‐up process for a straw composed of cellulose micro and nanofibers. The sealing is achieved upon strong hydrogen bonding among straw constituents with no need for adhesives. A radar plot compares the cellulose hybrid straw with commercial PLA and paper straws. Reproduced with permission.^[^ ^327^ ^]^ Copyright 2020, Wiley‐VCH. e) Thermally induced degradable adhesives enabled by CNCs within an epoxy matrix. Reproduced with permission.^[^ ^328^ ^]^ Copyright 2020, American Chemical Society.

In addition to the above‐mentioned examples, transient technology is being explored in other interesting applications. A notable example is the development of transient aerial robots aimed at reducing the adverse environmental impact issues originating from potential drone malfunction and subsequent loss in the environment. Gelatin‐crosslinked micro‐fibrillated cellulose (MFC) cryogels were fabricated by pouring an aqueous MFC/gelatin suspension into a mold, followed by freeze‐drying.^[^ ^324^ ^]^ This fabrication enabled the formation of strongly intertwined structures by the triple‐helical segments of gelatin, resulting in highly porous structures with a mechanical strength of 0.5 MPa and a modulus of 12 MPa. Such a highly porous structure with a high stiffness‐to‐weight ratio enables full aerial drone functionality (**Figure** **22**a). Rapid biodegradation was observed as indicated by complete weight loss after 40 days under simulated aerobic composting conditions at 58 °C (ISO 20200), although the electronics still had to be collected to avoid e‐waste contamination. Similarly, transient gliders have also been developed using potato starch with a humidity‐responsive bi‐layer actuator based on gelatin/TEMPO‐oxidized CNFs and shellac (≈70 wt% loss in 40 days).^[^ ^325^ ^]^

Additionally, the reversible bonding mechanisms in nanocelluloses could offer an environmentally sustainable solution to plastic pollution. For instance, Liu et al. 3D printed a high‐porosity graphite‐CNF composite aerogel that could be easily recycled through re‐dispersion in water in a few seconds with stirring (Figure 22b).^[^ ^326^ ^]^ The reversible hydrogen bonding mechanism in the graphite‐CNF composite was also exploited to obtain an aerogel that completely disappeared after 8 days in the natural ambient environment (Figure 22c). Here, the cellulose fraction is naturally decomposed into glucose via enzyme‐assisted hydrolysis, while the graphite goes back into the carbon cycle. This hydrogen bonding approach has also been exploited to obtain degradable straws using a combination of CNF and cellulose microfiber from sugarcane bagasse with no additional binders.^[^ ^327^ ^]^ A good hydrostability (tenfold increase over commercial paper straws) without compromising the degradability (disintegration into fragments after 4 months in a natural environment) was achieved, outperforming the properties shown by popular degradable straws thanks to their tensile strengths of ≈70 MPa and strain at break of 12.7%. Inspired by the reinforcement principle in natural wood, this design was further evolved by integrating 26 wt% lignin into micro–nanocellulose fibers (a lignin content similar to natural wood).^[^ ^329^ ^]^ After hydrogen bond formation through rolling, the wet films were baked in an oven at 150 °C to infiltrate the lignin and provide a polyphenolic binder behavior, rendering straws that began degradation after 100 days in the environment (Figure 22d).

**Figure 23:**
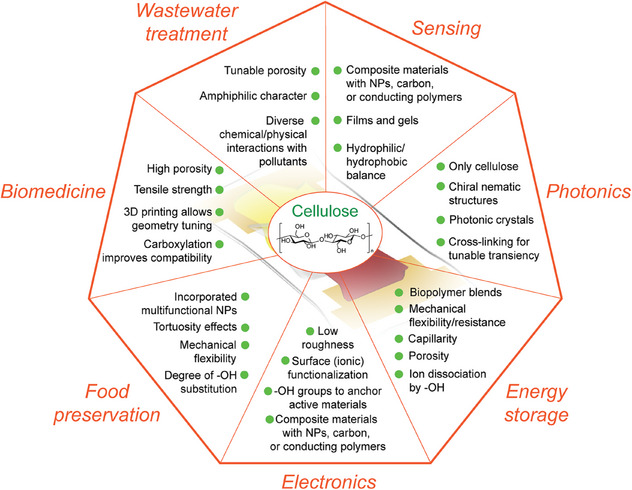
Overview of favorable features of cellulose that have inspired the design of transient devices, organized by the relevance of each feature for the selected categories of applications discussed in this review.

Various efforts have been made to facilitate recycling approaches and redirect materials (e.g. adhesives) back into the production cycle instead of discarding them. This rather new strategy could enhance material circularity rates and should attract further attention in the near future. Conventional adhesives are based on highly crosslinked epoxy structures, which impede the disassembly (and subsequent reusability and recyclability) of joined parts. In contrast, the use of thermally degradable adhesives with much lower degradation temperatures than the joined components offers a facile method to retrieve high‐value materials. In this context, Kang et al. exploited the well‐recognized poor thermal stability of sulfonate groups on CNCs to induce accelerated thermodegradation events in epoxy resins and facilitate the cohesive failure of the adhesive material at >200 °C (Figure 22e).^[^ ^328^ ^]^ At the same time, CNCs mechanically reinforced the material, increasing the shear strength by 31% when working under ambient temperatures. In this context, Chen et al. recently reported a novel strategy to utilize CNFs to obtain photo‐detachable adhesive materials. They fabricated a PAA/CNF/Fe^3+^ supramolecular network with dopamine adhesive handles, resulting in remarkable mechanical and adhesive properties. When irradiated with UV light, the Fe^3+^ ions are reduced to Fe^2+^, resulting in the dissociation of coordination complexes, changing the adhesion energy by 93%. Very interestingly, this approach proved to be universal for biomacromolecules and petrochemical polymers, including gelatin, chitosan, alginate, starch, PAM, and PVA.^[^ ^330^ ^]^ Cellulose and its derivatives, either in the form of nanocelluloses, cellulose ethers, or cellulose esters, have advantageous properties of special interest for transient device fabrication. In this vein, **Figure** **23** briefly summarizes the main benefits of cellulosic materials in the development of competitive multifunctional transient devices, organized by application area. To facilitate navigation through the discussed work, **Table** **4** maps the used materials, specific end‐user applications, the property to highlight, and the corresponding reference for each entry.

**Table 4:** Comparison of the transient applications enabled by cellulose and nanocellulose.

Application		Material	Property to highlight	Mechanism^a)^	Refs.
*Sensing*		Cellulose fibers from waste papers; graphite	Film: Formed by papermaking techniques. Gauge factors of 27 and easy disintegration in water.	T (H_2_O immersion)	[237]
		CNC & Ag	Film: London dispersion forces enable patterning accuracy. Gauge factor of 52. CNC transiency facilitates recycling.	T (H_2_O immersion)	[238]
		CNFs & MXene	Film: Pressure sensor. Complete degradation in soil after 240 days. Oxidized to TiO_2_ nanoparticles upon burning.	T (burning, in soil)	[239]
		TEMPO‐oxidized CNFs; 2D MoS_2_	Film: Cellulose provides an elastic matrix, complete dissolution in 6 days in PBS at 75 °C.	T (PBS, heat)	[240]
		TEMPO‐oxidized CNFs, PAA, LMNPs	Physical gel: High stretchability, conductivity, strain sensitivity, and transiency in 7 days.	T (H_2_O immersion)	[241]
		CNCs (sulfonated, carboxylated), deep eutectic solvent	Physical gel: Dual‐response in terms of resistivity and optical upon stretching, recycling at 50 °C due to dynamic bonding.	T (heating)	[242]
		TEMPO‐oxidized CNFs; PDA; PAM	Chemical gel: incorporated FeCl_3_ triggers Fenton‐like process to ensure quick degradation in water.	T (H_2_O immersion)	[243]
		Shellac & graphite flakes / carbon‐black	Shellac enables shear‐thinning inks for printing. Hydrophobic character provides a faster response over cellulosic substrates.	T (aerobic composting)	[244]
		Potato starch; carbonized polyimide	Flexible film: Gauge factor of 134. Complete disintegration in 10 min under running water.	T (water flush)	[245]
		Cellophane; filter paper; biodegradable thermoplastic	Sweat monitoring device: Material and fabrication processes are compatible with biodegradation both in soil and enzymatically.	T (composting)	[246]
*Photonics*		CNC films	Chiral nematic structures: Mechanical stimuli afford transient color.	T (stretching)	[265]
		Crosslinked CNC films	Chiral nematic structures: Crosslinking shifts color and changes water‐dissolution resistance.	T (H_2_O immersion)	[267]
		HPC in H_2_O (<20%)	Photonic crystal: Freestanding or by direct nanoimprint of HPC onto desired substrates. Very rapid transiency in water.	T (H_2_O immersion)	[222]
		Pure cellulose in trifluoroacetic acid/trifluoroacetic anhydride	Photonic crystal: Spectral transparency; glass‐like refractive index. 60% weight loss in soil degradation in < 100 days.	T (soil & seawater)	[268]
*Energy*		Cellulose membranes (Ahlstrom)	Primary battery: Biodegradation faster than pure cellulose; single‐use; quinone‐based redox chemistry.	P + T (biotic anaerobic)	[270]
		1.18 mm thick Whatman filter paper	Primary battery: Capillarity‐transpiration process; biodegradation in 18 days producing environmental nutrients.	P + T (biotic aerobic)	[271]
		TEMPO‐oxidized CNFs and CNCs	Supercapacitor: CNFs and CNCs are used to form shear‐thinning gels. Able to design complex shapes and geometries.	T (aerobic composting)	[272]
		Lithiated CNCs into a PVA membrane	Secondary battery: Increased porosity, ionic conductivity, Li^+^ transport number, no dendrite formation.	T (H_2_O immersion)	[274]
		Cellulose aerogel‐gelatin solid electrolyte	Secondary battery: High specific capacity, biocompatibility were under in vitro and in vivo tests.	T (buffered proteinase K solution)	[275]
		CMC and agarose hydrogel, glutaraldehyde crosslinking	Secondary battery: Long‐term stability afforded by –OH groups, mechanical adaptability, high ionic conductivity.	T (aerobic composting)	[276]
		Fungal chitin nanofibril hydrogel, glutaraldehyde crosslinking	Secondary battery: Amorphous character/interconnected 3D structure/oxygen functional groups facilitate ion transport. Transient battery disperses in water at 70 °C.	T (H_2_O immersion, heating)	[11]
*Electronics*		TEMPO‐oxidized CNFs coated with bisphenol A‐based epoxy	Bipolar transistors, Schottky diodes, passive inductors/capacitors, CMOS digital devices: Comparable performance to conventional counterparts. Partial degradation by brown rot fungus.	T (fungal biodegradation)	[278]
		CNF substrate + nanocellulose as resistive switching layer	Non‐volatile memory device: A 7.4 nm surface roughness enables thin active materials to reach 99.3 vol% CNFs in the device. Complete degradation in natural soil in 26 days.	T (natural soil burying)	[279]
		Hydrolyzed cellulose substrate	Thin‐film transistors: Extensive fabrication time; complete degradation in 30 days (pH 4.6, 1 mg·mL^−1^ cellulase solution).	T (pH 4.6 aqueous buffer)	[280]
		BC	Triboelectric nanogenerators: PDA enhances power density to generate 43 V. Complete disintegration within 15 min at 90 °C.	T (acidic solution, heating)	[281]
		CNCs	Triboelectric nanogenerators: CNCs enhance the dielectric constant and accelerate hydrolytic chain scission of PHB.	T (H_2_O, pH 12)	[284]
		Nitrated cellulose paper	Organic light‐emitting diode: Phase change material triggers combustion reactions for complete transiency in 10 s.	T (flame or 200 °C heating)	[285]
		CNC shear‐thinning ink	Recycling: Sonication for >95% retrieval of harmful components (CNTs, graphene).	T (ultrasonication)	[286]
		Cellulose filter paper; CNCs	Recycling: Aqueous two‐phase extraction recovers >70 wt% of SWCNTs and >90 wt% of graphene after 5 cycles.	T (tip sonication)	[282]
		Lignin	Recycling: 96.9% recovery rate of room‐temperature liquid metal active components.	T (0.5 M NaOH)	[283]
		n‐type CNCs, p‐type CNCs	Ionic diodes: Rectification ratio of 70; potential transiency.	Claimed biodegradable	[287]
		CNFs	Radio frequency identification antennas: Lignin crosslinked for 4.7 nm roughness; complete degradation in a day.	T (rain)	[288]
*Films and coatings for food preservation*		CNC coatings	Hindered gas diffusion: Tortuosity enhances barrier properties.	Claimed biodegradable	[293]
		CNF coatings	Protection against rot: Reduce the enzymatic browning of banana peels.	T (composting)	[294]
		Freestanding CNC films	Antimicrobial composite: 60 wt% loss after 35 days in simulated aerobic composting. Nanoparticles (Ag_2_O) reduce susceptibility to degradation.	T (composting)	[295]
		Cellulosic bioplastic	Processability: Covalent adaptable network reconstruction design principle	T (soil burial)	[296]
		CMC/MOFs	Antimicrobial composite: Complete degradation in 45 days in soil (25 °C, 65–75% RH). MOFs endow antimicrobial and fruit preservation properties.	T (buried in soil)	[297]
		Cellulose derivatives in general	Degradation tunability: ‐OH substitution reduces the susceptibility of cellulose to enzymatic hydrolysis exponentially.	T (enzymatic degradation, composting)	[102]
		Cellulose diacetate	Photocatalytic degradation: TiO_2_ incorporated to speed up transiency upon illumination.	T (sunlight exposure)	[299]
		CA	Built‐in enzyme activity: Physically entrapped enzymes speed‐up biodegradation.	T (enzymatic degradation, composting)	[100]
					
*Biomedicine*		Oxidized regenerated cellulose	Textiles: Commercially available knitted fabric.	P (degradation in vivo)	[301]
		TEMPO‐oxidized CNCs	Blood clotting: Enhances hemostatic efficiency in alginate films/sponges. Complete degradation in 3 weeks (subcutaneous implantation).	P (degradation in vivo)	[302]
		TEMPO‐regenerated cellulose	Dissolvable sutures: Faster in vitro degradation (increased molecular chain spacing + lower molecular inter‐atomic force).	T (0.9 wt% NaCl)	[303]
		TEMPO‐oxidized CNFs	High shape fidelity: Chitosan reinforced for 3D printing. x2 mechanical modulus and accelerated weight loss at pH 7.4.	T (phosphate buffer, pH 7.4)	[304]
		TEMPO‐oxidized CNFs	Cardiac tissues: Electrically conducting scaffolds (PPy, PGS) mimicking cardiac tissues. Slow degradation for long‐term.	T (MES @ pH 6; PBS @ pH 7.4)	[305]
		TEMPO‐oxidized CNCs	Low elastic modulus: Embedded and carbon dots for dynamic cell tracking; mechanical properties mimic soft living tissues.	T (PBS and trypsin)	[306]
		BC	Tunable mechanical properties: Mold‐guided growth for auxetic structures with 48–456 MPa; long‐term tissue support uses.	T (aqueous, pH 7.5 and 5)	[307]
		Oxidized BC	Bone regeneration: BC reinforces chitosan scaffolds, boosts hydroxyapatite growth, and improves flexibility/water retention.	T (PBS with lysozyme)	[308]
		Oxidized BC	Nerve repair: Scaffold function for sciatic nerve repair.	T (PBS solution)	[309]
		Mercerized BC	Blood vessel replacement: Adequate geometry provided upon mercerization.	P (degradation in vivo)	[310]
		Oxidized BC	Increased transiency rate: Speeds up the hydrolytic degradation of soybean protein (≈3 to ≈50% after 48 days in PBS).	T (PBS solution)	[311]
	*Wastewater treatment*	Regenerated cellulose/SiO_2_	Reusable dye removal: Removal rates of 99‐99.9%, retains 80% of removal efficiency after 5 cycles.	T (activated sludge)	[322]
	*Aerial robots*	Gelatin crosslinked MFC	Extremely lightweight material: Aerogels with high stiffness‐to‐weight ratio for drones.	T (aerobic composting)	[324]
		Gelatin crosslinked MFC	Humidity response: Humidity responsive bi‐layer structure for gliders.	T (aerobic composting)	[325]
*Other uses*	*Adhesives*	CNCs with sulfonate groups	Accelerated degradation: Facilitates thermoset adhesive thermodegradation for disassembly and reuse/recycling.	T (temperature increase)	[328]
		TEMPO‐oxidized CNFs and graphite	Recyclability: Reversible hydrogen bonding between components.	T (rain)	[326]
		TEMPO‐oxidized CNFs, dopamine, and PAA	Reversibility: Adhesion energy varied by 93%	T (UV light)	[330]
	*White pollution prevention*	Cellulose micro‐ and nanofibers	Physical crosslinks: Strong hydrogen bonding balances hydrostability / mechanical properties (keeping degradability).	P (environmental disposal)	[327]
		Cellulose micro‐ and nanofibers, lignin	Natural binder: Strong hydrogen bonding and lignin infiltration provide enhanced mechanical properties.	P (environmental disposal)	[329]

^a)^ “Passive (P)” or “Triggered (T)” transiency

MFC: micro‐fibrillated cellulose; CNC: cellulose nanocrystal; TEMPO‐CNF: TEMPO‐oxidized cellulose nanofibrils. PBS: phosphate‐buffered saline; MOFs: metal organic frameworks; PDA: polydopamine; PHB: polyhydroxybutyrate.

## 5 Future Perspectives: Environmental Sustainability, Performance, and Industrialization

Considerable efforts are being directed worldwide to find new solutions to environmental issues related to pollution, the depletion of non‐renewable resources, and global warming. In this context, transient devices enabled by renewable materials have clear benefits over their conventional (non‐renewable, non‐degradable) counterparts that directly contribute to resource depletion and environmental pollution.^[^ ^25^, ^331^ ^]^ The exploitation of materials originating from renewable carbon feedstock offers a feasible solution to circumvent the generation of large quantities of greenhouse gases. In fact, the IPCC Sixth Assessment Report shows that 70.5% of anthropogenic greenhouse gas emissions (41.6 Gt CO_2_ for the year 2019) originate from fossil resources.^[^ ^332^ ^]^ Thus far, this review has highlighted the trade‐offs between the often mutually exclusive characteristics such as environmental sustainability, degradability, cost, mechanical/thermal stability, and electrochemical performance, and how these aspects can be balanced.^[^ ^333^ ^]^ In this section, we summarize the main environmental benefits of transient devices according to the 12 fundamental principles of Green Chemistry,^[^ ^334^, ^335^ ^]^ followed by a life cycle‐focused discussion to provide a holistic viewpoint on the potential of transient devices in a Circular Economy. To conclude, the performance benefits, limitations, and roadblocks or the implementation of biobased materials, as well as their potential nanotoxicity are explored. This section emphasizes the advantages and challenges that must be addressed in the near future for the successful widespread adoption of transient devices into the global economy.

### 5.1 Environmental Sustainability

#### 5.1.1 Green Chemistry Principles

The renewable materials explored in this review present clear benefits for environmental sustainability. However, to gain a wider picture it is also important to consider Green Chemistry principles in the assessment of a material's environmental impact. For example, biocolloids (e.g. nanocellulose, nanochitin) are often isolated using harsh chemical reactions that generate an excessive amount of secondary toxic substances.^[^ ^336^, ^337^ ^]^ Therefore, novel pathways that are more resource‐efficient and avoid corrosive and toxic acids such as H_2_SO_4_ or HCl should be developed, considering that under a “business as usual” scenario, the production of 1 kg of CNCs by conventional methods generates up to ≈2000 kg of wastewater.^[^ ^213^, ^214^ ^]^ The development of novel pathways would limit waste generation (1^st^ principle), improve atom efficiency (2^nd^ principle), reduce hazards during synthesis (3^rd^ principle), and limit solvent use (5^th^ principle). Accordingly, shifting the isolation of nanocellulose or nanochitin from acid hydrolysis to mechanically‐ or enzymatically‐assisted methods is encouraged.^[^ ^338^, ^339^ ^]^ ILs and deep eutectic solvents should also be further exploited to develop transient biomass‐derived devices (e.g. in the form of ionogels), allowing for a reduction in the toxicity and CO_2_ footprint originating from conventional solvents.^[^ ^340^ ^]^ Atom economy for biomass isolation could be improved by targeting high‐yield processes and cellulosic sources such as cotton from poplar genotypes (containing up to 52 wt% of cellulose).^[^ ^341^ ^]^ Fortunately, the byproducts of biomass isolation can be considered safe as they usually consist solely of soluble polysaccharides, proteins, lipids, and minerals.

Most biobased materials adhere to the 4^th^ Green Chemistry principle (*Design Benign Chemicals*), showing reduced toxicity in comparison with their conventional inorganic or petroleum‐based analogs. However, when downsized to the nanoscale, biocolloids have the potential to react with biological targets, so special care should be taken when considering biomedical applications in particular. The potential toxicity of nanocelluloses is elaborated further in Section 5.3.

As biobased material isolation is often conducted under relatively mild temperatures (45 °C for CNCs, 90–104 °C for chitin nanocrystals, 85 °C for fungal chitin nanofibers)^[^ ^214^ ^]^ and ambient pressures (6^th^ principle), energy requirements are moderate; of course, advancements in this field are ongoing. The 7^th^ Green Chemistry principle (*Use of Renewable Feedstocks*) is fulfilled intrinsically by biobased materials, but technical challenges can emerge in developing processes that allow for biomass conversion from locally available resources in a carbon‐neutral or ‐negative manner. Additionally, the use of derivatizing processes can be minimized during biomass valorization (8^th^ principle), avoiding additional reagent use and waste.

An improvement in the sustainability of biocolloids could be realized through the shift from stoichiometric to catalytic reagents (9^th^ principle). Enzymatic processes are already widely employed for this purpose, offering high selectivity and efficiency. Biobased materials and transient devices are perhaps the most compatible with the 10^th^ Green Chemistry principle (*Design for Degradation*), as they can break down into innocuous degradation products after their lifetime is eclipsed. Finally, the 11^th^ (*Real‐Time Analysis for Pollution Prevention)* and 12^th^ (*Inherently Benign Chemistry for Accident Prevention*) principles relate to specific on‐site processes that make biobased materials safer for the health of workers and the environment. As an example, aqueous reactions at mild temperatures and ambient pressures are clearly valuable for the prevention of pollution and accidents when compared to conventional materials fabrication.

#### 5.1.2 Life Cycle Considerations

Green Chemistry principles can offer a useful framework to conceptualize the design of novel environmentally sustainable transient materials. However, future design must also adopt a life cycle mindset, wherein devices or component materials are repeatedly circulated at the highest value. According to the ISO 14001 international standard for environmental management systems, a product's life cycle is defined as the *“consecutive and interlinked stages of a product (or service) system, from raw material acquisition or generation from natural resources to final disposal.”* Therefore, the nature of raw materials, the fabrication, the use phase, and the EoL phases should be considered during the design phase.

Renewable raw materials such as lignocellulosic biomass, algae, or insects can be obtained locally. This is especially relevant in the sustainable energy landscape, where natural graphite (77% of global production by China), cobalt (70% of global production by the Democratic Republic of the Congo), and other critical raw materials are often subject to supply chain bottleneck issues.^[^ ^342^, ^343^ ^]^ Oil, the main feedstock for fossil‐derived materials such as polyvinylidene fluoride (PVDF) (actuator applications) or PP/PE (battery separators), can be a source of environmental contamination, and its use presents safety hazards during production.

The fabrication phase benefits from the inherent characteristics of biobased materials, using water‐based solvents under ambient conditions. Biocompatible ILs such as the [Bmim][TFSI] can be also incorporated to include additional functionalities.^[^ ^274^ ^]^ This contrasts with the processing of petroleum‐based polymers, which often involves flammable and toxic^[^ ^344^ ^]^ solvents such as dimethylformamide, dimethylacetamide, or N‐methyl‐2‐pyrrolidone.^[^ ^345^ ^]^

The use phase could potentially be negatively affected by the lower thermo‐mechanical stability of biobased materials when compared with inorganic or petroleum‐derived materials. In this sense, attempts to improve the high‐temperature stability, the mechanical resistance, or durability of transient devices should be carried out to extend their operating lifespan. A pertinent example of this is the fabrication of self‐healing gels that regenerate at room temperature without external stimuli.^[^ ^346^ ^]^ Alternatively, this lower stability could be taken advantage of to facilitate controlled transiency and material reuse.

After transient devices have accomplished their function, they can be disposed of under controlled conditions at their EoL so their constituent materials can re‐enter the biological cycle via composting or anaerobic digestion,^[^ ^25^ ^]^ This avoids the accumulation of non‐degradable material in the environment while closing material loops, a cornerstone of the Circular Economy mindset. Devices that cannot be recovered due to their specific application or operational environment (e.g. remote sensing in precision agriculture),^[^ ^347^ ^]^ can even be designed such that their degradation products provide nutrients to the surrounding environment after the device is retired.

In all, nanocelluloses obtained from diverse sources can be integrated into transient devices, which then undergo degradation events to close material loops. Redispersion and dissolution afford nanocelluloses that can be reincorporated into new devices, while enzymatic degradation, acid hydrolysis, UV‐exposure or thermal degradation repurpose embedded cellulosic biogenic carbon into biological nutrients for the surrounding environment. This results in a continuous cycle of renewable carbon between the technosphere (transient devices), biosphere (in the soil, biomass), and the atmosphere (released biogenic CO_2_, later captured by biomass).

#### 5.1.3 Environmental Impact Metrics

Impact metrics that quantify environmental effects can guide the future ecodesign of transient devices by pointing toward lower‐impact solutions. To this end, standardized environmental impact quantification methodologies such as life cycle assessment (LCA), standardized by ISO 14040/44, have gained considerable attention for the validation and prospective improvement of environmentally sustainable solutions. Importantly, not only CO_2_ emissions but also additional environmental afflictions such as acidification, water consumption, toxicity, or eutrophication can be quantified.^[^ ^348^, ^349^ ^]^ The information obtained can therefore serve as a base for further improvement following eco‐design strategies. Despite its usefulness, we found no reports on LCA applied for transient devices.^[^ ^350^ ^]^

To guide the design of sustainable transient devices, we summarize in **Table** **5** the global warming potential (quantified in CO_2_ equivalents per kilogram of material) of the most relevant biobased materials with the potential to be applied in transient devices. To aid in understanding, the materials have been grouped according to their origin. We note that these numbers should be considered with care due to a lack of harmonization when performing such analyses. Different system boundaries (*cradle‐to‐gate, cradle‐to‐cradle*…), process scalability (lab‐scale vs bench‐scale), and impact assessment methods (ReCiPe 2016, TRACI, CML, etc.) that categorize the results into diverse impact categories are often applied. Additionally, some studies allocate a credit of 3.66 kg·CO_2_ equiv. per kilo of carbon due to the biogenic CO_2_ uptake from biomass growth.^[^ ^351^ ^]^ With values ranging from 0.1 to 11.0 kg·CO_2_ equiv.·per kilo, certain biobased polymers such as methyl cellulose, CMC, calcium alginate, chitin, or Kraft/Organosolv lignin show comparable CO_2_ footprints to petroleum‐derived polymers, which benefit from fully optimized industrial production due to their long history. Importantly, biocolloids such as CNCs or CNFs show a two‐to‐three orders of magnitude increase in global warming potential (GWP), reaching values of 12.6 to 1160 kg∙CO_2_ equiv.∙kg^−1^ (dry basis) for nanocelluloses. These results are explained by a combination of factors including low production capacities (thus increasing the energy consumption share), poor production yields, or heavy use of reagents such as HCl, H_2_SO_4_, or H_2_O_2_ with large embodied carbon footprints that are not recirculated.^[^ ^214^ ^]^ However, this larger footprint can be balanced in some ways: pretreatments such as TEMPO‐oxidation can be applied to reduce energy requirements for CNF production and limit the resulting CO_2_ emissions by ≈50%,^[^ ^216^ ^]^ while process upscaling to reach production capacities of 300 tons∙year^−1^ can lower the GWP values of CNCs to values as low as 12.6 CO_2_ equiv. per kg.^[^ ^213^ ^]^ Interestingly, nanocellulose production using bottom‐up approaches (e.g. bacterial nanocellulose) can also offer significant environmental benefits, reducing the CO_2_ footprint to 16.7 kg CO_2_ per kg.^[^ ^220^ ^]^ Additional improvements in efficiency can be made during the use phase, considering for example that biocolloids can form (hydro)gels at very low weight fractions (e.g. 0.3 wt% for TEMPO‐oxidized CNFs),^[^ ^352^ ^]^ reducing the overall CO_2_ footprint per kilo of material by ≈200 times over its parent material.

**Table 5:** Global warming potential, terrestrial acidification, fossil resource scarcity, marine eutrophication, and water use per kilogram of material. Due to the different impact assessment methodologies in the literature, certain impact categories are not reported*.

Material	Global warming potential (kg·CO_2_ equiv.∙kg^−1^)	Terrestrial acidification (kg∙SO_2_ equiv. kg^−1^)	Fossil resource scarcity (kg∙oil equiv. ∙kg^−1^)	Marine eutrophication (kg∙N equiv. ∙kg^−1^)	Water use (m^3^∙kg^−1^)	Refs.
Polypropylene granulate	3.65	0.008	–	9.44 × 10^−5^	0.02	Ecoinvent 3.10, ReCiPe 2016 Midpoint (H)
Polystyrene (PS)	3.95	0.009	–	2.47 × 10^−5^	0.05	
Polyvinylidene fluoride (PVDF)	16.53	0.046	–	3.00 × 10^−3^	0.12	
PLA	2.0–8.0 (petroleum) ≈ 0.5 ^a)^ (bio‐based)	0.013	–	1.80 × 10^−3^	0.13	^[^ ^226^ ^]^, Ecoinvent 3.10, ReCiPe 2016 Midpoint (H)
Methyl cellulose (E461)	3.69	–	–	–	–	[235]
CMC (E466)	4.4–10.7	–	–	–	–	Ecoinvent 3.10, ReCiPe 2016 Midpoint (H)
CNCs	12.6–1160	0.855	92.05	5.29 × 10^−3^	3.85	[214]
CNCs (up‐scaled)	12.6–16.6	–	–	–	≈130–900	[213]
CNFs	190–1160	6.44 (for 807 kg CO_2_ GWP)	402.80 (for 807 kg CO_2_ GWP)	0.05 (for 807 kg CO_2_ GWP)	28.2 (for 807 kg CO_2_ GWP)	[216, 217]
BC	16.7	0.043	6.56	4.00 × 10^−3^	0.47	[220]
Kraft lignin	0.1–4.0	–	–	–	–	[356]
Organosolv lignin	1.54–2.14	0.040	–	–	–	[357]
Lignin nanoparticles	36.6–199	–	–	–	≈1.51–4.35	[358]
Sodium alginate (E401)	21.30	–	–	–	–	[359]
Carrageenan (E407)	2.4 (India)	–	–	–	–	[360]
Calcium alginate	2.12–10.10	0.043–0.055	3.02–5.45	3.10–3.26 × 10^−3^	0.18	[361]
Chitin nanocrystals (ChNCs, crustacean)	105–907	0.387–3.604	28.09–248.71	12.18–87.30 × 10^−3^	1.73–21.44	[214]
Chitosan	59.22	0.197	11.31	3.14 × 10^−3^	34.45	[362]
Chitin nanofibrils (ChNFs) from fungi	18.5	0.066	4.91	1.51 × 10^−3^	1.01	[214]
Pectin	9.69	0.05	–	0.00 (freshwater)	9.54	[363]
Gelatin	2.34	–	–	–	–	[364]

^a)^ accounting for biogenic carbon capture.

Aside from the GWP, Table 5 demonstrates that the impact of cellulosic and other biobased materials in areas such as terrestrial acidification, marine eutrophication, and especially in water use can be a significant drawback. This can be explained by the heavy usage of water or acidic reagents in current biomass processing methods. In this context, it should be noted that the use of woody biomass waste streams for cellulose extraction purposes could provide additional environmental benefits over other cellulosic feedstock such as cotton, which requires 2.78 m^3^ of water per kg for processing.^[^ ^353^ ^]^ Besides, future studies should consider additional environmental metrics not only focused on impacts but also on the efficiency of resource use. Of particular relevance is the circularity rate, roughly defined as the share of used material resources originating from recycled waste streams (note that several methodologies exist to calculate this parameter).^[^ ^354^ ^]^ In this context, biobased materials are well‐equipped to enhance the global circular material use rate, which is unfortunately in decline, dropping from 9.1% in 2018 to 7.2% in 2023.^[^ ^355^ ^]^

### 5.2 Performance Considerations in Transient Device Design

Many of the transient devices reported so far show poorer performance than their non‐renewable/non‐biodegradable competitors. These differences originate from the very beginning of the design process, as a range of materials fulfilling the stringent requirements for transiency is limited. Indeed, organic cathodes for batteries usually show reduced operating voltages and specific capacitances compared to most inorganic cathodes.^[^ ^365^ ^]^ Similarly, the conductivities of materials susceptible to biodegradation processes (i.e. conducting polymers grown on biobased materials) remain below the state‐of‐the‐art results obtained for composite materials bearing non‐degradable carbonaceous (graphene) or inorganic nanofillers (silver nanowires). In spite of these drawbacks, remarkable examples can be found in which transient devices show improved performance over their non‐degradable analogs. For example, CNC‐based photonic materials show reliable color characteristics over time. As opposed to pigments or dyes, which can fade over time due to irreversible molecular chain scission events, the color of CNC systems originates from their stable nanoarchitecture.^[^ ^366^ ^]^ Therefore, nanocellulosic photonic materials present improved color reliability over time, as well as the ability to degrade at EoL.

Durability under operating conditions (especially cyclic mechanical stresses or high temperatures) is a source of concern in transient devices. However, several examples of biomass‐derived materials have demonstrated an extended operating lifespan, especially in the energy storage field. For instance, a crosslinked agarose/CMC in an aqueous 2 M ZnSO_4_ solution has shown an impressive long‐term stability of 10 000 cycles at a current density of 1 A·g^−1^ in a ZIB, outperforming most of its non‐degradable competitors.^[^ ^276^ ^]^ Biobased materials have also proven efficient in lengthening the operation time of devices by alleviating mechanical stresses during battery cycling by buffering volume expansion upon Li^+^ insertion/extraction,^[^ ^367^ ^]^ or inhibiting the growth of dendritic metal, preventing premature short‐circuiting in batteries.^[^ ^274^ ^]^ These works suggest a bright future for transient devices with extended operating lifespans.

Ensuring a rapid device degradation once the external trigger has been applied is important to open new possibilities in applications such as anti‐counterfeiting labels. Certain biobased materials such as CA display slow degradation kinetics (degradation times extending up to 3 years), making them potentially transient, but only over impractical periods of time.^[^ ^88^ ^]^ As discussed in Section 3.4, crosslinking biopolymer gels through labile or reversible chemical bonds, or through the incorporation of low MW additives, could be exploited to trigger the disintegration of the materials when subjected to UV irradiation, heat, moisture, or pH changes.^[^ ^368^ ^]^

### 5.3 Biocompatibility and Nanotoxicity

Transient devices are particularly suitable for in vivo biomedical uses (implant administration,^[^ ^304^ ^]^ cardiac pacemakers,^[^ ^369^ ^]^ electrocardiogram signal detectors),^[^ ^370^ ^]^ where they can monitor or treat specific diseases and then be naturally reabsorbed by the body, circumventing the second surgery intervention required for retrieval of conventional non‐degradable devices.^[^ ^371^ ^]^ In this context, biocompatibility and nanotoxicity should be considered with special care. Nanocelluloses have been widely reported as nontoxic and biocompatible. However, we advise caution in applying this label too broadly. A precedent exists in the literature for the toxicity of high aspect ratio nanomaterials such as carbon nanotubes (CNTs), where particle aerosolization (an effect also occurring with nanocelluloses during industrial production) can cause acute toxic effects.^[^ ^372^ ^]^ As nanocelluloses have a similarly high aspect ratio, it may not be surprising to observe increased toxicity when compared to bulk cellulose.^[^ ^372^ ^]^

In general, the most pressing concerns for nanocelluloses are the potential to cause inflammation in tissues, as well as routes to carcinogenicity. To examine these effects, a number of tests have been extensively performed. In vitro studies can be used to quantify cytotoxic, genotoxic, and immunotoxic effects, which are related to changes in cell viability, damage to DNA, and changes in immune cell counts/behavior, respectively.^[^ ^372^ ^]^ In vivo studies can accomplish all of the above, in addition to providing information on tissue damage, and whether or not effects induced by the introduction of nanocelluloses spread systemically to the rest of the body through the vascular or lymphatic systems. Critically, studies must be carefully designed to represent the use case of the final material, as toxicity and cell uptake are very dependent on the cells (in vitro) or organism (in vivo) that is used.^[^ ^373^ ^]^ For example, while the uptake of nanocelluloses into mammalian cells is generally quite low (except for macrophages), bacterial cell uptake can show completely different results.^[^ ^374^ ^]^ In bacterial in vitro tests, CNCs with an aspect ratio of ≈18 have been shown to lack any inhibitory effect for the growth of four common environmental bacteria: *K. pneumonia, S. aureus, E. coli*, and *S. epidermidis*.^[^ ^375^ ^]^ Other in vitro studies employing mammalian cells have also reported the lack of toxicity against Caco‐2 colon carcinoma cells (1000 µg∙mL^−1^ dose),^[^ ^376^ ^]^ macrophages (700 µg∙mL^−1^ dose, 24 h), and keratinocytes (500 µg∙mL^−1^ dose, 24 h).^[^ ^377^ ^]^ On the in vivo side, DeLoid et al. have shown that feeding rats a diet of either CNFs or CNFs + fat for 5 weeks resulted in no significant increase in reactive oxygen species production, no changes in blood or serum biomarkers, and no pathological changes as confirmed through histological analysis.^[^ ^378^ ^]^ In fact, for rats fed a diet of CNFs + fat, very low levels of weight loss were observed compared to rats fed a diet of only fat, suggesting that CNFs could play a role in hindering fat absorption, opening possibilities for safer weight loss treatments. While in vivo studies such as this do exist, most studies employ some kind of bacteria to verify the lack of toxicity for each given material. The underpinning theme to many of these studies is the extraction of nanocelluloses from a novel biomass source, followed by characterization and toxicity profiling with a variety of bacterial species. It remains to be seen whether this approach is acceptable for actually testing the biocompatibility of nanocelluloses in the specific environment in which they are to be used, leading to the wider trend of nanocelluloses being treated as biocompatible by default, when the more rigorous approach would be to design specialized biocompatibility assays properly suited for actual use cases.

As an example of a specialized assay, Li et al. investigated the dose, length, and aspect ratio‐dependent toxicity of CNCs and CNFs on Kupffer cell phagocytes, liver cells, and Hepa 1–6 tumor cells.^[^ ^373^ ^]^ Among a range of CNCs with aspect ratios of ≈9–27 and CNFs with aspect ratios of 83 and 172, they found that while all CNCs induced varying levels of toxic effects, where the “medium”‐sized CNCs (aspect ratio ≈13) were the most severe. The toxicity was attributed to the generation of reactive oxygen species in liver cells, triggering caspase‐induced apoptotic cell death.^[^ ^373^ ^]^

The examples discussed thus far have considered only native, relatively unmodified nanocelluloses. Predictably, surface modifications can also greatly influence the interactions of nanocelluloses with cells and tissues.^[^ ^189^ ^]^ As an example, CNFs modified with cetyltrimethylammonium bromide (CTAB) or polyethyleneimine have both been shown to reduce fibroblast viability and proliferation compared to unmodified CNFs in vitro.^[^ ^379^ ^]^ These studies highlight the need to carefully consider the impact of surface modification for transient devices designed for in vivo operation. Along with the toxicity of surface‐grafted groups themselves, charge density and grafted surface groups can greatly influence the degree of cellular uptake, potentially amplifying otherwise mild negative effects.^[^ ^374^ ^]^ We direct the reader to recent reviews by Tortorella et al.^[^ ^380^ ^]^ and Mujtaba et al.,^[^ ^381^ ^]^ as well as several recent research articles^[^ ^373^, ^382^, ^383^, ^384^ ^]^ for more information on the impact of nanocellulose modifications on toxicity. To conclude, the status of nanocelluloses as a bio‐derived material may sometimes result in false assumptions about their inertness in biological systems. Thankfully, most evidence points to minimal toxicity. However, we stress the importance of conducting tests using a model appropriate for the end use of the device, as toxicity responses can depend on the morphology of the nanocelluloses, surface modifications, and the environment and cells to which they are exposed.

### 5.4 Challenges Toward Industrialization

Despite the optimistic outlook, the large‐scale use of nanocelluloses in transient devices presents several challenges that must be addressed. For example, the proliferation of nanocellulose‐based materials is highly constrained by economics. Until recently, existing laboratory‐scale nanocellulose processing methods have been cost‐prohibitive for industrialization. However, operational expenses have been notably lowered upon process up‐scaling, with the exponential growth of nanocellulose production capacities in recent years. Currently, the main players in nanocellulose production are located in Canada (CelluForce Inc., Anomera Inc., FPInnovations, Blue Goose Biorefineries Inc., Alberta–Pacific Forest Industries Inc., Cellulose Lab Inc., Kruger Inc.), United States (USDA Forest Products Lab, GranBio, University of Maine), Northern Europe (Sappi, CelluComp, Melodea, Fibenol OÜ, Navitas, Borregaard), and Eastern Asia (Nippon Paper, Oji Paper, Chuetsu Pulp and Paper, Sugino Machine, Seiko PMC, Tianjin Rumi Ltd., Tianjin Haojia Cellulose Co. Ltd., Hangzhou Yuehua Technology Co.).^[^ ^213^, ^385^ ^]^ As of 2022, global production capacities reached ≈700 tonnes for CNCs (dry basis), ≈1850 tonnes for CNFs (dry basis), and even more for microfibrilated cellulose (≈21 400 tonnes).^[^ ^385^ ^]^ As a result, CNCs and CNFs are currently available at costs ranging from ≈1.5 to 50 USD·g^−1^ depending on the source, isolation procedure, quality, and surface modification. Interestingly, nanofibrillated cellulose can be purchased at a much lower cost of 20 USD·kg^−1^ thanks to the implementation of several semi‐industrial plants.^[^ ^386^ ^]^ Nevertheless, these costs remain above the price of the benchmark materials. As a result, the use of nanocellulose is primarily justified only in specific cases where it offers a tangible advantage over conventionally used materials such as PET foils (useful in flexible electronics, <1.0 USD·kg^−1^),^[^ ^387^ ^]^ or petroleum‐derived battery separators (<0.6 USD·m^−2^).^[^ ^388^ ^]^ Representative examples where the implementation of nanocelluloses is justified in terms of cost include sensors for biomedical uses, anti‐counterfeiting labels for enhanced security, primary batteries for point‐of‐care diagnostic devices or agricultural monitoring, or adhesives to facilitate part disassembly and subsequent recycling. As a practical example, a recent study has reported a reasonable cost of 121 USD∙kg^−1^ for lignin‐nanocellulose flexible substrates for electronic applications.^[^ ^288^ ^]^ Therefore, it is our hope that progress being made in nanocellulose production, with a projected compound annual growth rate of 20.1% in 2023–2030 (reaching USD 1520 million),^[^ ^389^ ^]^ will result in enhanced competitiveness and even lower production costs.

The lack of rapid and scalable processing routes for nanocellulosic products is also a source of concern as it hinders industrialization. In this context, solvent‐casting routes are appropriate for fundamental scientific studies but need to be replaced by additive manufacturing, roll‐to‐roll processing, or melt‐extrusion (as for conventional plastics) to progress in the practical implementation of transient cellulosic devices. Potential consumer safety issues resulting from the tiny size of cellulosic particles (inhalation risk, cellular internalization) also need to be considered. Despite these limitations, we are confident that the near future will witness an accelerated adoption of cellulosic materials, particularly considering the clear performance and environmental benefits of nanocelluloses under specific applications, global regulatory policies aiming to reduce fossil‐carbon consumption, and the increased awareness on environmental sustainability and renewability.

The proliferation of transient devices would also ideally require adherence to standardized verification methods. Some standards already exist, such as ISO 14855, which states that in order to meet standards for biodegradability, a material must achieve ≥ 90% mass loss in less than 6 months (under aerobic biodegradation conditions with activated sludge).^[^ ^390^ ^]^ To avoid greenwashing by promising the use of “bio‐based” materials in products, EN 16785,^[^ ^391^, ^392^ ^]^ EN 16640,^[^ ^393^ ^]^ or ISO 16620^[^ ^394^, ^395^ ^]^ are useful. These can be used to determine quantitatively the bio‐based carbon content of materials through radiocarbon and elemental analysis. We believe that strict enforcement of such standards is necessary for the development of circular transient devices. Academic researchers should also consider performing biodegradation tests using existing published standards, as shown by Mittal et al., who performed laboratory‐scale aerobic composting of a transient Zn‐ion battery by ISO 20200 methods,^[^ ^276^, ^396^ ^]^ or Reyes et al. who used the same method to demonstrate degradation of poly(lactic acid)‐ and polyhydroxybutyrate‐coated cellulose aerogels.^[^ ^397^ ^]^ Adherence to these standards could be promoted by academic publishers to encourage increased reproducibility and accelerate transient materials research progress. Finally, standardization of testing methods for the environmental toxicology of nanomaterials, including (nano)cellulose, is highly important – projects such as HARMLESS EU are aiming to push such initiatives forward by developing an integrated approach to nanomaterials impact management through verified case studies.^[^ ^398^, ^399^, ^400^ ^]^ Overall, we would like to stress the great relevance of standardization as the rate of transient materials development increases around the globe.

## 6 Conclusion

We expect that in the near future, we will witness the widespread proliferation in the design and use of transient devices as the economy shifts toward circular and sustainable patterns. While still in its relative infancy, the development of transient materials with a sharp focus on the 10^th^ Green Chemistry principle, “Design for Degradation”, is integral for keeping technological development on a sustainable course. In this quest, material selection is paramount – materials should be sustainably derived, renewable, and possess a wide range of opportunities for modification and tunability – all properties that (nano)cellulosic materials can offer. To this end, this work has critically reviewed the current state‐of‐the‐art transient technology enabled by (nano)cellulosic materials, with special emphasis on transiency mechanisms, applications and sustainability considerations. Additionally, to provide a wider perspective on the place of (nano)cellulosic materials in the current design landscape, the contributions of other renewable materials, such as chitin, have been summarized and future development pathways provided. The potential environmental benefits of transient devices are highlighted, considering a life‐cycle thinking perspective. Performance‐related challenges still remain, and these highlight not only the advantages of (nano)cellulose‐based transient materials but also the bottlenecks that must be addressed. The pressing need to transition toward higher circularity rates requires careful consideration of the trade‐offs between material renewability, carbon footprint, biodegradability, non‐toxicity, avoiding harmful degradation product generation (especially when using modified nanocelluloses), and performance. With careful, evidence‐driven design, a future that is green and waste‐free, where materials are continuously circulated within the bio‐cycle and the techno‐cycle, can be realized, and current systems relying on fossil‐based materials in the sensing, actuator or energy storage fields can be replaced by transient devices, thus preserving the earth for future generations.

## Conflict of Interest

The authors declare no conflict of interest.

## Author Contributions

L.J.A. and E.L. wrote the first draft of the manuscript and prepared the figures. L.J.A. and E.L. collaborated on all edits of the final version of this manuscript. M.J.M assisted with planning review structure, reviewing, and edits.
